# Supplementary material for: C-reactive protein is a broad-spectrum capsule-binding receptor for hepatic capture of blood-borne bacteria
Source: EMBO J. 2025 Nov 10;44(24):7364–94. doi: 10.1038/s44318-025-00623-w (PMC12705745; doi:10.1038/s44318-025-00623-w)
Supplement: Supplementary file 1 — Appendix [file 44318_2025_623_MOESM1_ESM.pdf]

Appendix

C-Reactive Protein Is A Broad-Spectrum Capsule-Binding Receptor For Hepatic Capture of Blood-Borne Bacteria

D.Y. Chen *et al.*
Corresponding authors: Jianxun Qi, jxqi@im.ac.cn; George Fu Gao, gaof@im.ac.cn; Jing-Ren Zhang, zhanglab@tsinghua.edu.cn

**Contents**	**Page number**
Appendix Figure S1	1
Appendix Figure S2	2
Appendix Figure S3	3
Appendix Figure S4	4
Appendix Figure S5	5
Appendix Figure S6	6
Appendix Figure S7	7
Appendix Figure S8	8
Appendix Figure S9	9
Appendix Figure S10	10
Appendix Figure S11	11
Appendix Table S1	12
Appendix Table S2	23
Appendix Table S3	25
Appendix Table S4	30
Appendix Table S5	31
Appendix Table S6	33
Appendix Table S7	34

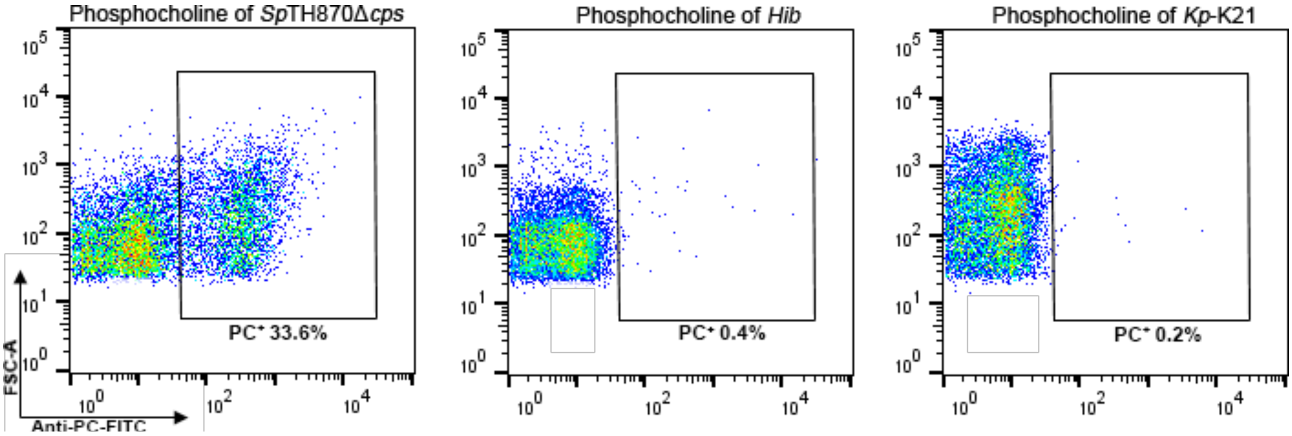

Appendix Figure S1. Detection of PC expression by flow cytometry.

S. pneumoniae TH870Δ*cps*, *H. influenzae* type b (*Hib*), and *K. pneumoniae* K21 were stained with anti-PC antibody. PC-positive (PC^+) populations were identified by flow cytometry, with gated regions indicating the percentage of PC^+ cells in each sample. This PC detection is part of the same experiment as Fig. 1G.

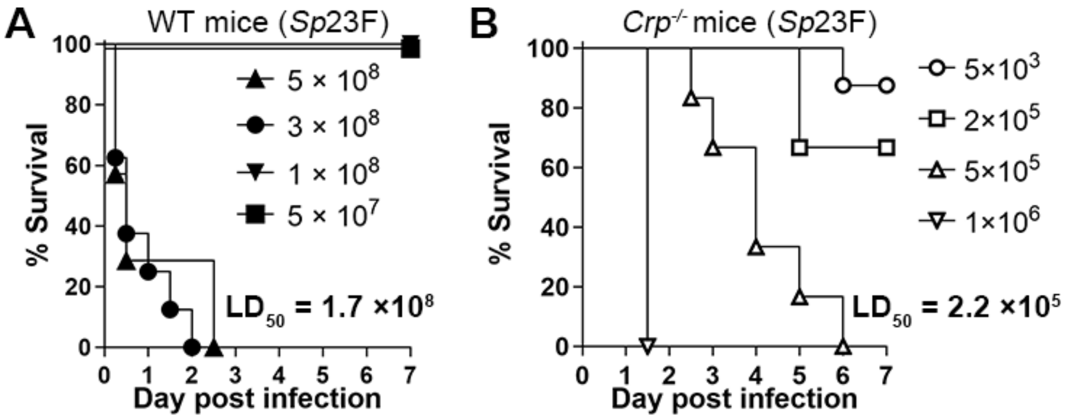

Appendix Figure S2. Critical role of CRP in *Sp*23F infection.

(**A**) The infection dose limit of *Sp*23F infection in WT mice. WT mice were intravenously (i.v.) infected with different doses of *Sp*23F. n = 3-8.

(**B**) The protection limit of CRP against *Sp*23F. *Crp*$^{-/-}$ mice were infected i.v. with different doses of *Sp*23F. n = 3-8.

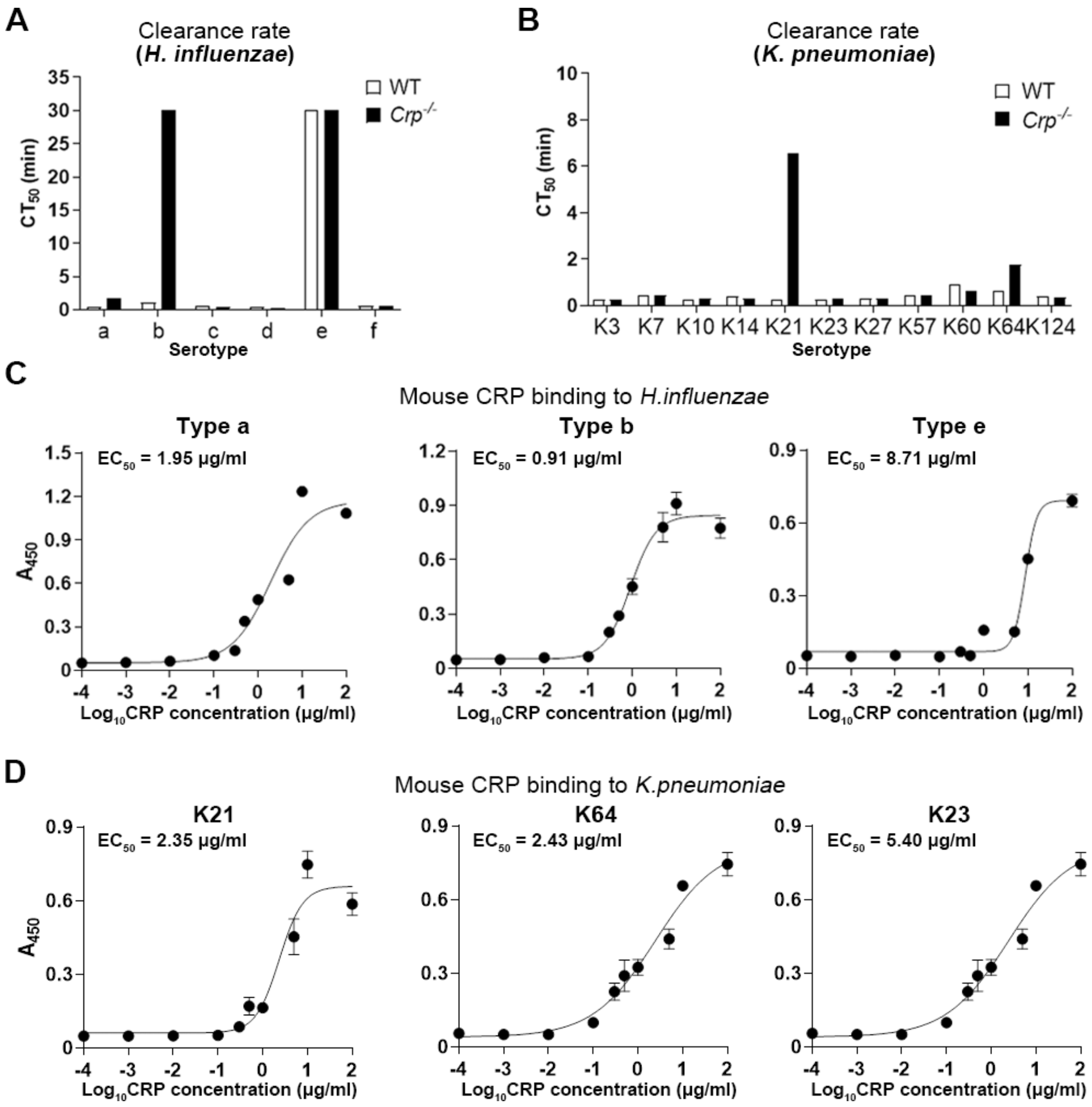

Appendix Figure S3. The contribution of CRP to the clearance of multiple Gram-negative pathogens.

(A) The role of CRP in the clearance of *H. influenzae* from bloodstream. WT and *Crp*$^{-/-}$ mice were i.v. infected with 10^7 CFU. CT_{50} were calculated as in Fig. EV3A. n = 1-3.

(**B)** The role of CRP in the clearance of *K. pneumoniae* from bloodstream. WT and *Crp*$^{-/-}$ mice were i.v. infected with 5×10^6 CFU*,* CT_{50} were assessed and presented as in (A). n = 1.

(**C**) r-mCRP binding to serotype a, b, and e of *H. influenzae* was assessed as in Fig. 1D. n = 3.

(**D**) r-mCRP binding to K21, K64, and K23 of *K. pneumoniae* was assessed as in Fig. 1D. n = 3.

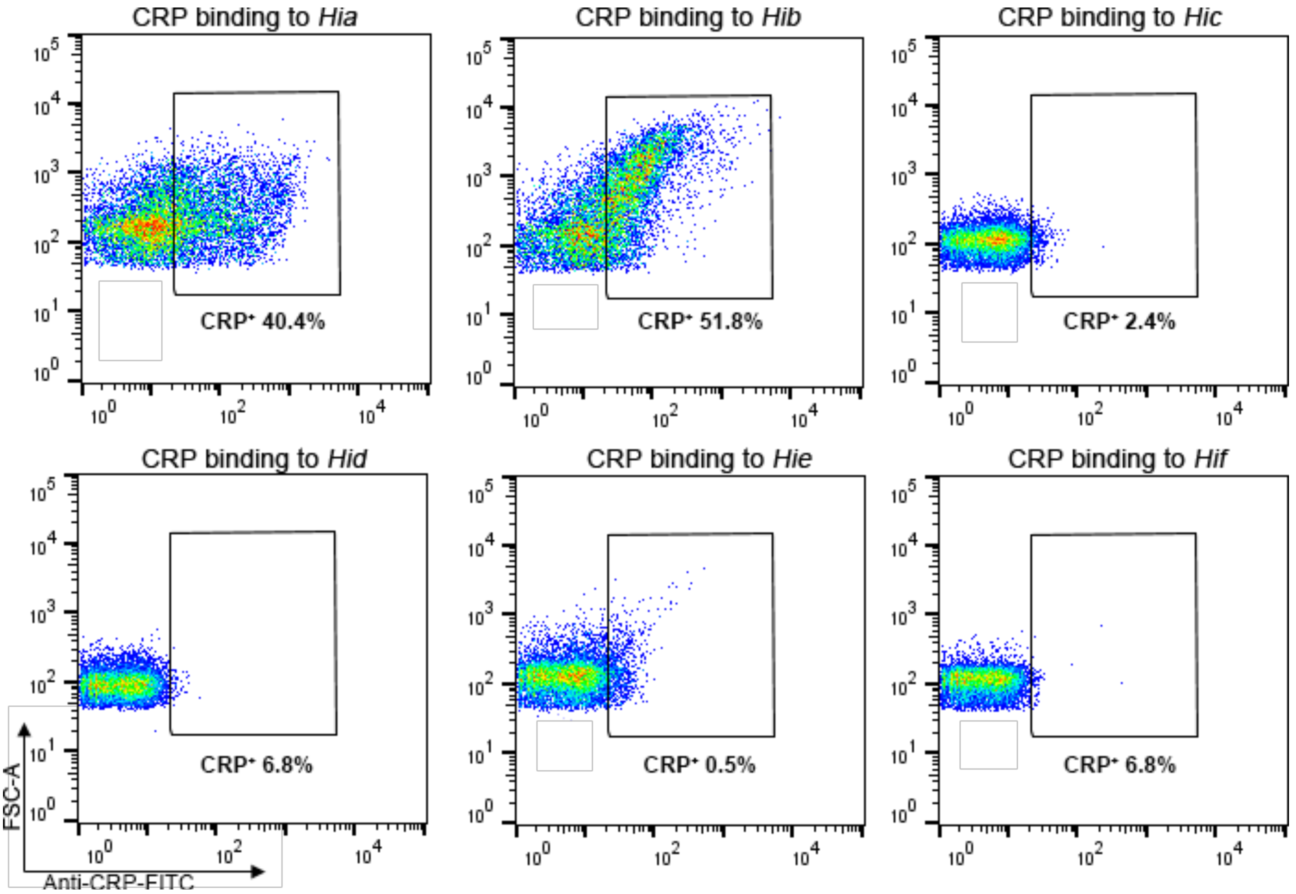

Appendix Figure S4. Detection of mCRP binding to *H. influenzae* by flow cytometry.
10^6 CFU *H. influenzae* were incubated with mCRP, stained with anti-CRP antibody, and analyzed by flow cytometry. The percentage of CRP-positive (CRP^+) cells is indicated within the gates, as shown in Fig. 1F.

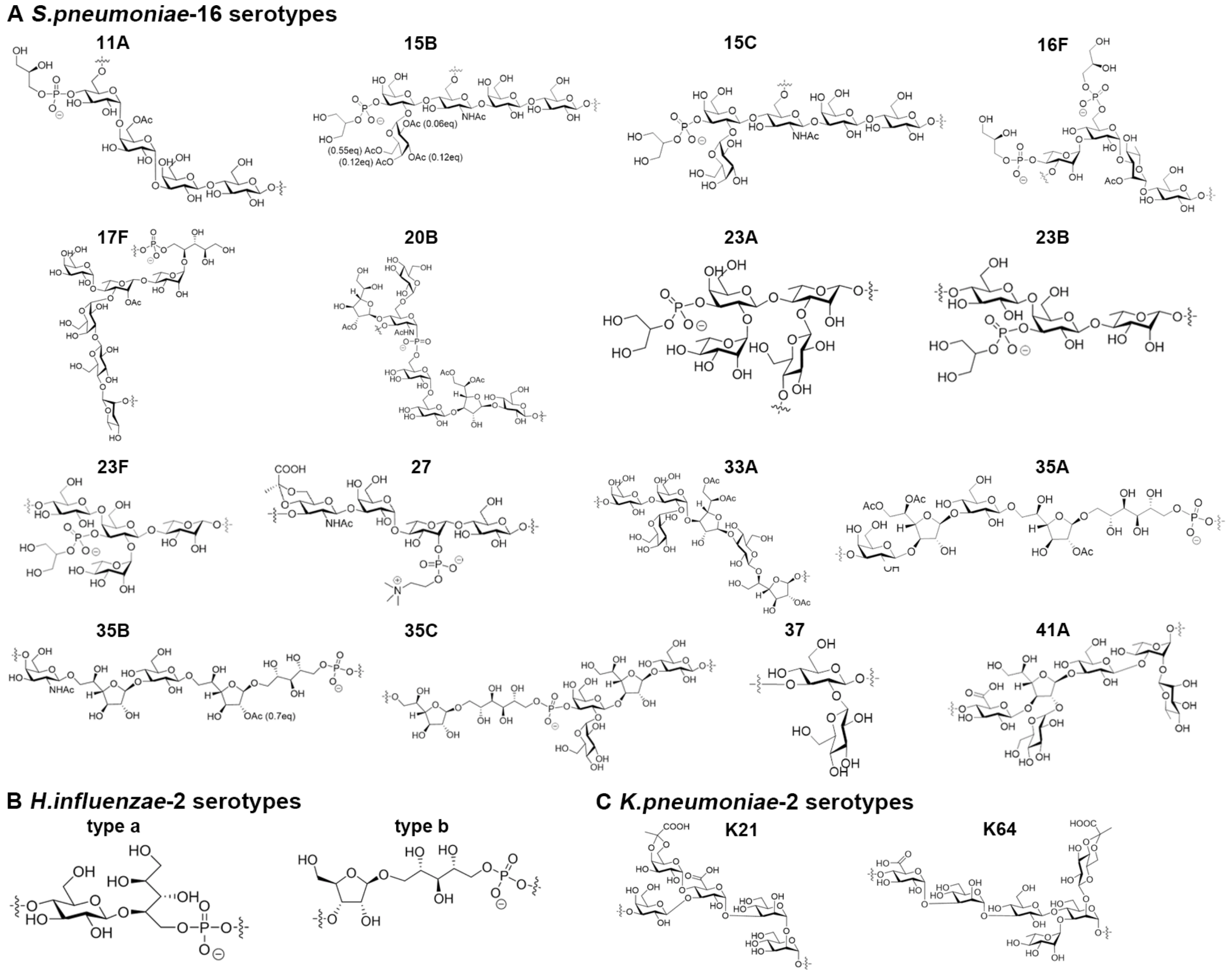

Appendix Figure S5. Chemical architectures of CRP-binding capsules.

(**A**) Chemical architectures of CRP-binding pneumococcal capsules (CPS11A, 15B, 15C, 16F, 17F, 20B, 23A, 23B, 23F, 27, 33A, 35A, 35B, 35C, 37 and 41A).

(**B**) Chemical architectures of *H. influenzae* types a and b capsules.

(**C**) Chemical architectures of *K. pneumoniae* type K21 and type K64 capsules.

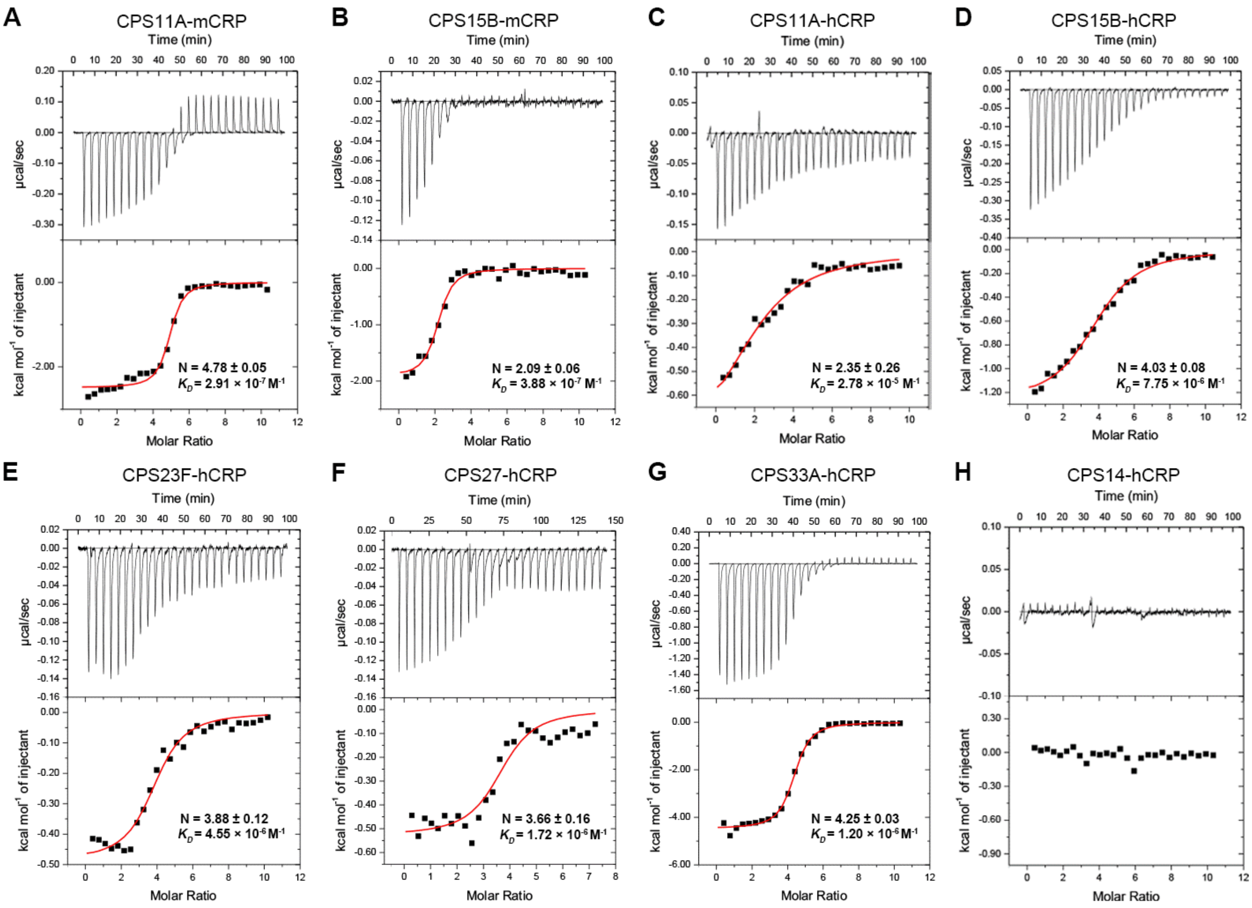

I The binding kinetics of mCRP and CPSs

CPS	Stoichiometry (N)	Dissociation constant (K_D, M)	Binding enthalpy (ΔH, cal/mol)
11A	4.78 ± 0.05	(2.91 ± 11.8) × 10^{-7}	-2490 ± 41.75
15B	2.09 ± 0.06	(3.82 ± 14.1) × 10^{-7}	-1921 ± 73.69
23F	4.71 ±0.04	(2.46 ± 12.3) × 10^{-7}	-2276 ± 33.07
27	2.90 ± 0.07	(1.1 ± 12.8) × 10^{-5}	1468 ± 47.01
33A	35.4 ± 6.61	(4.9 ± 12.6) × 10^{-4}	-4148 ± 1314
14	ND	ND	ND

ND, not detectable.

J The binding kinetics of hCRP and CPSs

CPS	Stoichiometry (N)	Dissociation constant (K_D, M)	Binding enthalpy (ΔH, cal/mol)
11A	2.35 ± 0.26	(2.79 ± 12.4) × 10^{-5}	-882.0 ± 128.7
15B	4.03 ± 0.08	(7.75 ± 64.5) × 10^{-6}	-1921 ± 73.69
23F	3.88 ±0.12	(4.55 ± 17.6) × 10^{-6}	-486.6 ± 19.76
27	3.66 ± 0.16	(1.72 ± 3.73) × 10^{-6}	1468 ± 47.01
33A	4.25 ± 0.03	(1.20 ± 10.5) × 10^{-6}	-4476 ± 44.02
14	ND	ND	ND

ND, not detectable.

Appendix Figure S6. The binding affinity of CRP to capsules.

(**A-H**) Isothermal Titration Calorimetry (ITC) data for the interaction of CPS11A-mCRP (A), CPS15B-mCRP (B), CPS14-mCRP (C), CPS11A-hCRP (D), CPS15B-hCRP (E), CPS23F- hCRP (F), CPS27- hCRP (G), CPS33A- hCRP (H), and CPS14-hCRP (I). Data after base-line integration and concentration normalization were shown and the stoichiometry (N) and the dissociation constant (K_D) were indicated in the plot. CPS14, negative control.

(**I**) The summary of ITC data for the interaction of mCRP and capsules.

(**J**) The summary of ITC data for the interaction of hCRP and capsules.

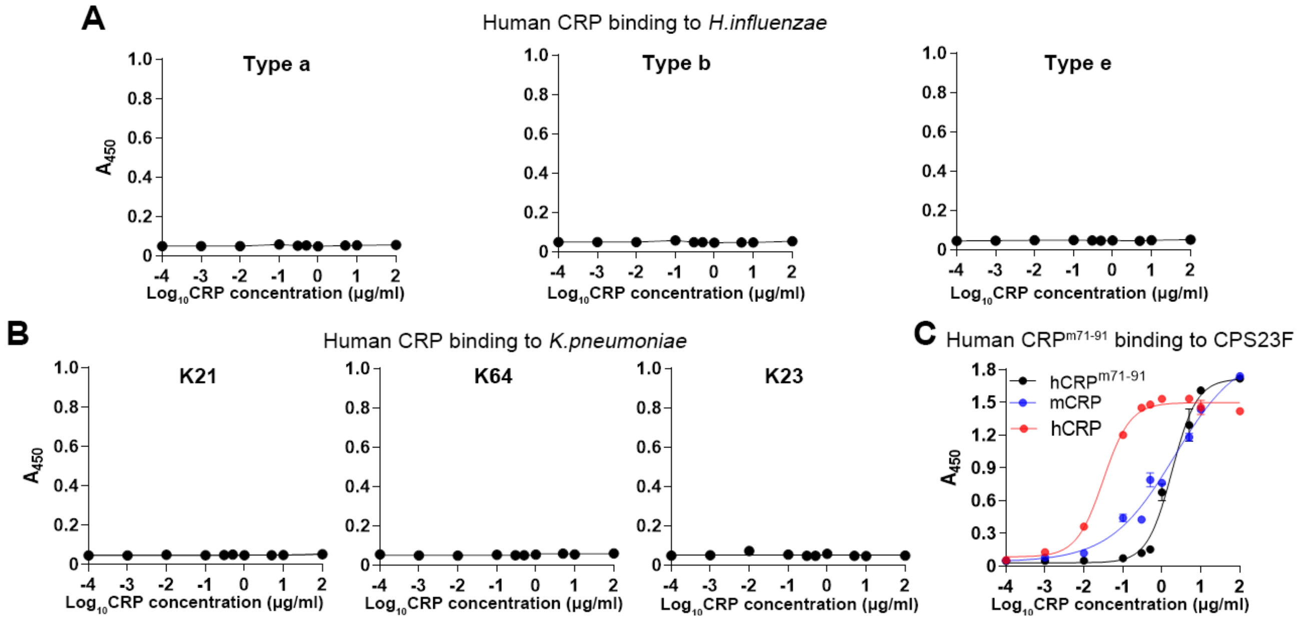

Appendix Figure S7. Human CRP binding to Gram-negative bacterial capsules.
(**A**, **B**) r-hCRP binding to *H. influenzae* and *K. pneumoniae*, respectively. CPS-coated wells were incubated with different concentrations of hCRP as in Fig. 1J. n = 3.
(**C**) CPS23F binding of a human-mouse CRP hybrid. CPS23F binding of wild type hCRP, mCRP and human-mouse CRP hybrid (m71-91) was determined and presented as in Fig. 7I. n = 3.

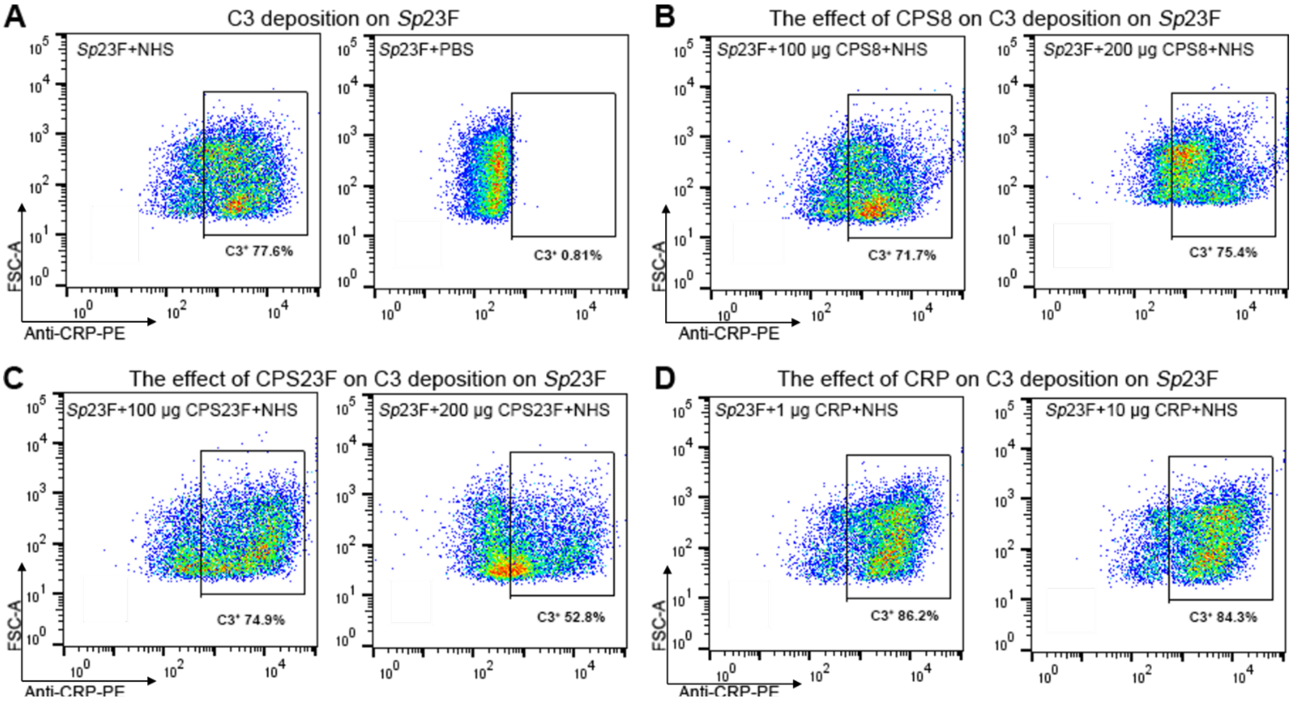

Appendix Figure S8. Evaluation of human C3 activation on pneumococcal capsules by flow cytometry.

(**A**) C3 deposition on serotype-23F *S. pneumoniae*. The 10^6 CFU of serotype-23F *S. pneumoniae* (*Sp*23F) were incubated with normal human serum (NHS) or PBS at 37°C for 30 min, followed by flow cytometric analysis to assess C3 deposition as in Fig. 7C.

(**B**, **C**) Inhibition of C3 deposition by CPS8 (B) and CPS23F (C). Purified CPS23F or CPS8 (100 and 200 μg) were incubated with NHS at 37°C for 30 min. Then, 10^6 CFU of *Sp*23F was added, and C3 deposition was assessed by flow cytometry.

(**D**) The effect of exogenous CRP on C3 deposition. *Sp*23F was first incubated with purified human CRP (1 μg and 10 μg) at 37°C for 30 min, followed by incubation with NHS. C3 deposition on *Sp*23F was then analyzed by flow cytometry.

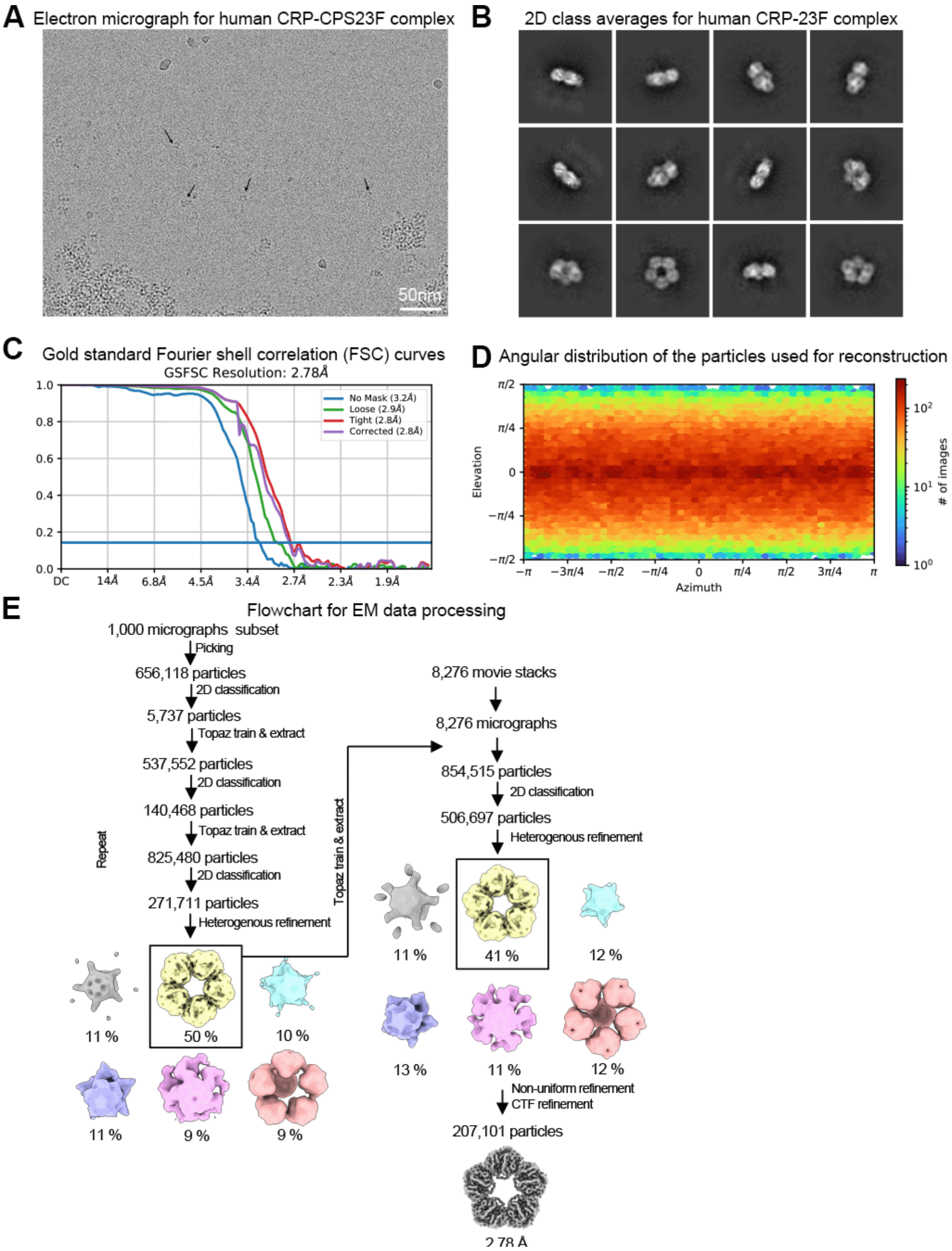

Appendix Figure S9. Cryo-EM structural analysis of the human CRP and CPS23F complex.
(**A**) Representative electron micrograph for the CRP-CPS23F complex.
(**B**) Representative 2D class averages for the CRP-CPS23F complex.
(**C**) Gold standard Fourier shell correlation (FSC) curves.
(**D**) Angular distribution of the particles used for reconstruction.
(**E**) Flowchart for EM data processing. Details can be found in Materials and Methods.

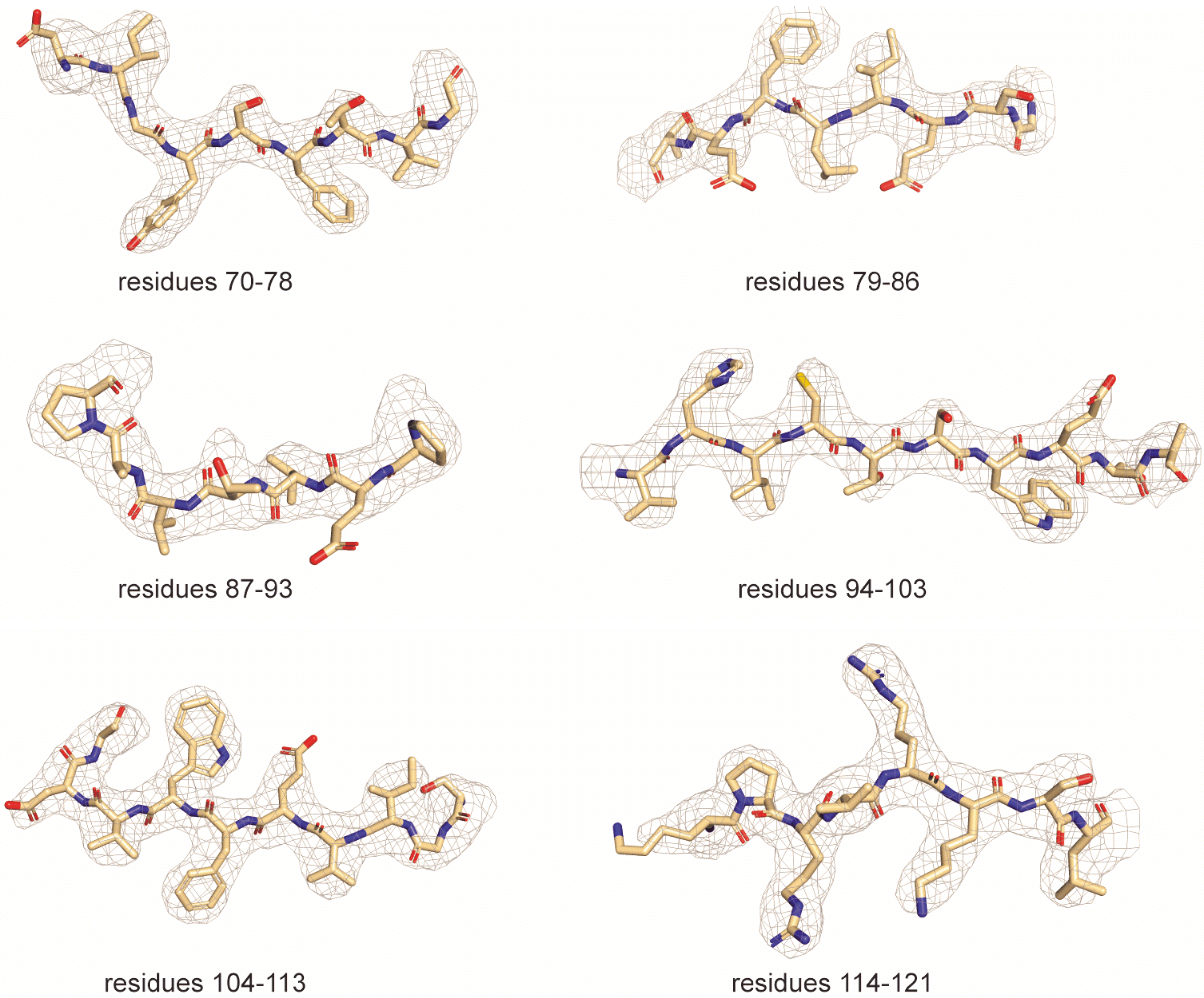

Appendix Figure S10. Representative densities and atomic models.
Representative densities and atomic models of several structural elements.

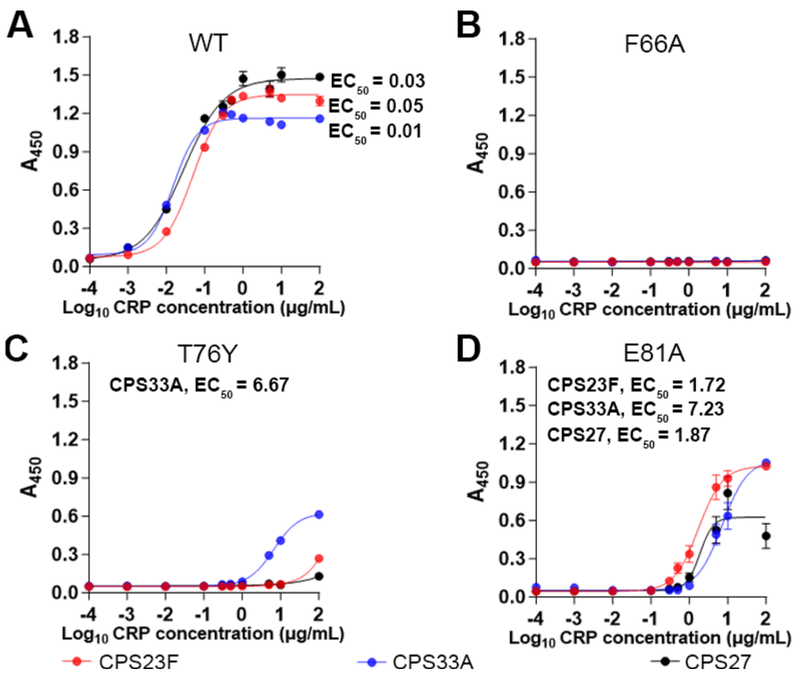

Appendix Figure S11. Capsule binding of human CRP mutants.

(**A**) Capsule binding to human CRP was assessed by ELISA at various concentrations using plate wells pre-coated with CPS23F, CPS33A, and CPS27. n = 3.

(**B**-**D**) The capsule binding of hCRP variants with mutants in amino acid residues (F66A, T76Y, and E81A) was tested for their interactions with CPS23F, CPS27, and CPS33A. n = 3.

Appendix Table S1. CPS23F-binding proteins from mouse KC and serum

Uniprot accession	Protein	Description	Abundance		CPS23F: CPS8 fold
			CPS23F	CPS8	
P14847	Crp	C-reactive protein	6.335×10^{10}	0	∞
G3UY98	Mettl3	N6-adenosine-methyltransferase subunit	1.001×10^{10}	0	∞
A0A0R4J213	Slc14a2	Urea transporter	2.010×10^{9}	0	∞
Q8CFG8	C1sb	Complement C1s-B subcomponent	2.000×10^{9}	0	∞
Q8CG16	C1ra	Complement C1r-A subcomponent	1.790×10^{9}	0	∞
A0A077S9N1	Lyz1	1,4-beta-N-acetylmuramidase C	9.678×10^{8}	0	∞
Q497N1	Rps26	40S ribosomal protein S26	1.296×10^{8}	0	∞
Q6IRU2	Tpm4	Tropomyosin alpha-4 chain	1.261×10^{8}	0	∞
A0A1W2P7I2	Epb41l2	Band 4.1-like protein 2	1.006×10^{8}	0	∞
Q8K469	Oas1g	2'-5' oligoadenylate synthase	9.195×10^{7}	0	∞
D3YXF5	C7	Complement component 7	9.184×10^{7}	0	∞
P39054-2	Dnm2	Isoform 2 of Dynamin-2	8.220×10^{7}	0	∞
G3X9G4	Dnm2	Dynamin GTPase	8.220×10^{7}	0	∞
Q8BPB5	Efemp1	EGF-containing fibulin-like extracellular matrix protein 1	7.518×10^{7}	0	∞
Q8BUT2	Dnm3	Dynamin GTPase	6.234×10^{7}	0	∞
Q9DCU6	Mrpl4	39S ribosomal protein L4, mitochondrial	5.564×10^{7}	0	∞
Q9DBS2	Tprg1l	Tumor protein p63-regulated gene 1-like protein	4.830×10^{7}	0	∞
O08601	Mttp	Microsomal triglyceride transfer protein large subunit	4.129×10^{7}	0	∞
A0A1W2P8C0	Epb41l2	Band 4.1-like protein 2 (Fragment)	3.798×10^{7}	0	∞
Q6ZPF4	Fmnl3	Formin-like protein 3	3.786×10^{7}	0	∞
Q99JB2	Stoml2	Stomatin-like protein 2, mitochondrial	3.022×10^{7}	0	∞
Q3TKM5	Idh3g	Isocitrate dehydrogenase [NAD] subunit, mitochondrial	3.015×10^{7}	0	∞
Q5SYD0	Myo1d	Unconventional myosin-Id	2.776×10^{7}	0	∞
Q3UQ44	Iqgap2	Ras GTPase-activating-like protein IQGAP2	2.561×10^{7}	0	∞

Uniprot accession	Protein	Description	Abundance		CPS23F :CPS8 fold
			CPS23F	CPS8	
Q9DCU9	Hoga1	4-hydroxy-2-oxoglutarate aldolase, mitochondrial	1.760×10^{7}	0	∞
Q3UFJ3	Pdha1	Pyruvate dehydrogenase E1 component subunit alpha	1.496×10^{7}	0	∞
P17426-2	Ap2a1	Isoform B of AP-2 complex subunit alpha-1	1.156×10^{7}	0	∞
P17427	Ap2a2	AP-2 complex subunit alpha-2	1.156×10^{7}	0	∞
A0A0U1RPW2	Tjp1	Tight junction protein ZO-1	9.041×10^{6}	0	∞
Q3UGJ5	Rasa3	Uncharacterized protein	8.876×10^{6}	0	∞
A0A0A0MQG2	Sptbn1	Spectrin beta chain, non-erythrocytic 1 (Fragment)	6.806×10^{6}	0	∞
Q8CC88	Vwa8	von Willebrand factor A domain-containing protein 8	3.542×10^{6}	0	∞
A0A3B0IP04	C1qa	Complement C1q subcomponent subunit A	4.576×10^{9}	1.772×10^{7}	258.289
P14106	C1qb	Complement C1q subcomponent subunit B	6.649×10^{9}	3.353×10^{7}	198.302
Q02105	C1qc	Complement C1q subcomponent subunit C	6.203×10^{9}	3.497×10^{7}	177.394
E9Q6C2	C1s1	Complement component 1, s subcomponent 1	3.206×10^{9}	2.660×10^{7}	120.543
G0YP42	NA	Anti-human Langerin 2G3 lambda chain	2.004×10^{9}	6.616×10^{7}	30.292
Q9Z2T6	Krt85	Keratin, type II cuticular Hb5	5.756×10^{10}	1.958×10^{9}	29.404
E9PVG8	9530053A07Rik	RIKEN cDNA 9530053A07 gene	1.336×10^{11}	5.573×10^{9}	23.963
P97290	Serping1	Plasma protease C1 inhibitor	2.084×10^{9}	9.106×10^{7}	22.886
P03995-2	Gfap	Isoform 2 of Glial fibrillary acidic protein	5.756×10^{10}	2.749×10^{9}	20.938
Q61171	Prdx2	Peroxiredoxin-2	1.546×10^{9}	1.028×10^{8}	15.036
Q3UAD6	Hsp90b1	HATPase_c domain-containing protein	6.149×10^{7}	4.119×10^{6}	14.928
Q0VBK2	Krt80	Keratin, type II cytoskeletal 80	2.116×10^{8}	1.424×10^{7}	14.860
Q8BIG7	Comtd1	Catechol O-methyltransferase domain-containing protein 1	6.638×10^{7}	5.160×10^{6}	12.866
A2AE15	Cfp	Complement factor properdin	1.705×10^{9}	1.355×10^{8}	12.580

Uniprot accession	Protein	Description	Abundance		CPS23F: CPS8 fold
			CPS23F	CPS8	
Q4L0E7	Krt77	Type II cytokeratin Kb39 (Fragment)	6.887×10^{10}	6.192×10^{9}	11.122
A0A0A0MQ76	Nop58	Nucleolar protein 58	8.422×10^{7}	7.702×10^{6}	10.934
A0A087WQ46	Nop58	Nucleolar protein 58 (Fragment)	8.422×10^{7}	7.702×10^{6}	10.934
Q91X70	C6	Complement component 6	2.174×10^{8}	2.044×10^{7}	10.635
Q61782	NA	Type I epidermal keratin mRNA, 3'end (Fragment)	7.079×10^{9}	6.987×10^{8}	10.132
E9Q3W4	Plec	Plectin	1.679×10^{8}	1.681×10^{7}	9.985
A0JLV3	Hist1h2bj	Histone H2B (Fragment)	1.004×10^{9}	1.033×10^{8}	9.721
P01027-2	C3	Isoform Short of Complement C3	4.169×10^{10}	4.364×10^{9}	9.554
P42125	Eci1	Enoyl-CoA delta isomerase 1, mitochondrial	2.033×10^{8}	2.212×10^{7}	9.187
O35643	Ap1b1	AP-1 complex subunit beta-1	1.818×10^{8}	2.000×10^{7}	9.094
Q8BH35	C8b	Complement component C8 beta chain	8.207×10^{7}	9.724×10^{6}	8.439
Q3UBI6	Rpl7	Uncharacterized protein	4.551×10^{8}	5.630×10^{7}	8.084
E9Q1Z0	Krt90	Keratin 90	1.122×10^{11}	1.408×10^{10}	7.971
Q60759	Gcdh	Glutaryl-CoA dehydrogenase, mitochondrial	1.316×10^{8}	1.653×10^{7}	7.962
P97350	Pkp1	Plakophilin-1	5.830×10^{8}	7.355×10^{7}	7.927
Q9CZJ2	Hspa12b	Heat shock 70 kDa protein 12B	3.900×10^{7}	5.013×10^{6}	7.779
P04104	Krt1	Keratin, type II cytoskeletal 1	2.255×10^{11}	2.973×10^{10}	7.585
P01632	Igkv7-33	Ig kappa chain V-I region S107A	6.204×10^{8}	8.380×10^{7}	7.403
P01027	C3	Complement C3	1.259×10^{11}	1.703×10^{10}	7.395
Q3UAC2	Rps3a1	40S ribosomal protein S3a	7.314×10^{7}	9.955×10^{6}	7.347
P50446	Krt6a	Keratin, type II cytoskeletal 6A	9.620×10^{10}	1.315×10^{10}	7.315
Q3UV11	Krt6b	Keratin, type II cytoskeletal 6B	9.620×10^{10}	1.315×10^{10}	7.315
Q8BGZ7	Krt75	Keratin, type II cytoskeletal 75	9.620×10^{10}	1.315×10^{10}	7.315
Q32P04	Krt5	Keratin 5	9.620×10^{10}	1.315×10^{10}	7.315
E9Q557	Dsp	Desmoplakin	2.236×10^{9}	3.075×10^{8}	7.273

Uniprot accession	Protein	Description	Abundance		CPS23F: CPS8 fold
			CPS23F	CPS8	
Q9DCV7	Krt7	Keratin, type II cytoskeletal 7	3.380×10^{10}	4.701×10^{9}	7.191
A0A2R8VHP3	Gm5478	Predicted pseudogene 5478	1.043×10^{11}	1.458×10^{10}	7.156
Q6NXH9	Krt73	Keratin, type II cytoskeletal 73	1.687×10^{11}	2.412×10^{10}	6.993
Q08EK4	Krt77	Keratin 77	1.954×10^{11}	2.837×10^{10}	6.887
Q5R3T5	G8(anti-MRBC)	G8(Anti-MRBC hybridoma) light chain (Fragment)	2.141×10^{8}	3.127×10^{7}	6.846
Q9R0H5	Krt71	Keratin, type II cytoskeletal 71	1.526×10^{11}	2.269×10^{10}	6.727
Q6IFZ9	Krt74	Keratin, type II cytoskeletal 74	1.526×10^{11}	2.269×10^{10}	6.727
Q9DCW4	Etfb	Electron transfer flavoprotein subunit beta	1.496×10^{8}	2.248×10^{7}	6.654
A0A0B4J1N0	Ighv1-76	Immunoglobulin heavy variable 1-76	8.139×10^{8}	1.227×10^{8}	6.632
H3BL60	C3	Complement C3 (Fragment)	4.223×10^{10}	6.812×10^{9}	6.199
Q61176	Arg1	Arginase-1	4.632×10^{8}	7.595×10^{7}	6.099
Q9D9P1	Chchd3	MICOS complex subunit Mic19	2.521×10^{7}	4.200×10^{6}	6.003
Q99LC5	Etfa	Electron transfer flavoprotein subunit alpha, mitochondrial	1.572×10^{8}	2.621×10^{7}	5.999
Q02257	Jup	Junction plakoglobin	1.809×10^{9}	3.017×10^{8}	5.996
A1L0X5	Krt78	Krt78 protein (Fragment)	9.494×10^{10}	1.672×10^{10}	5.680
Q8BP54	Pcx	Uncharacterized protein (Fragment)	3.810×10^{7}	6.941×10^{6}	5.490
A2A998	C8a	Complement component C8 alpha chain	6.800×10^{7}	1.241×10^{7}	5.480
Q3U026	Mogs	Uncharacterized protein	1.214×10^{8}	2.225×10^{7}	5.458
Q5SUA5	Myo1g	Unconventional myosin-Ig	8.922×10^{7}	1.760×10^{7}	5.071
P11679	Krt8	Keratin, type II cytoskeletal 8	1.475×10^{10}	2.946×10^{9}	5.005
Q4VA93	Prkca	Protein kinase C	6.742×10^{7}	1.356×10^{7}	4.972
Q99M73	Krt84	Keratin, type II cuticular Hb4	1.484×10^{10}	3.034×10^{9}	4.891
B9EIU2	C4a	C4a anaphylatoxin	7.359×10^{8}	1.506×10^{8}	4.886
Q6IFZ8	Gm5414	Predicted gene 5414	1.269×10^{10}	2.700×10^{9}	4.700
O70152	Dpm1	Dolichol-phosphate mannosyltransferase subunit 1	6.392×10^{7}	1.421×10^{7}	4.498

Uniprot accession	Protein	Description	Abundance		CPS23F: CPS8 fold
			CPS23F	CPS8	
A0A286YDB7	Ssr1	Signal sequence receptor subunit alpha (Fragment)	1.492×10^{8}	3.362×10^{7}	4.437
Q9EQK5	Mvp	Major vault protein	8.311×10^{7}	1.886×10^{7}	4.406
Q8VED5	Krt79	Keratin, type II cytoskeletal 79	3.393×10^{10}	7.781×10^{9}	4.361
P07744	Krt4	Keratin, type II cytoskeletal 4	1.632×10^{10}	3.771×10^{9}	4.328
P20918	Plg	Plasminogen	5.140×10^{8}	1.192×10^{8}	4.313
P06909	Cfh	Complement factor H	1.450×10^{9}	3.369×10^{8}	4.305
Q8C196	Cps1	Carbamoyl-phosphate synthase [ammonia], mitochondrial	1.486×10^{9}	3.479×10^{8}	4.270
Q3TQD9	Sardh	Uncharacterized protein (Fragment)	8.950×10^{7}	2.111×10^{7}	4.240
E9Q8B5	Cfhr4	Complement factor H-related 4	5.454×10^{8}	1.303×10^{8}	4.185
A4FUS1	Rps16	Rps16 protein	1.857×10^{8}	4.502×10^{7}	4.126
A0A3B2WBL1	Rpl10a	Ribosomal protein	1.184×10^{9}	2.949×10^{8}	4.015
Q9CR57	Rpl14	60S ribosomal protein L14	1.852×10^{8}	4.647×10^{7}	3.985
Q03265	Atp5f1a	ATP synthase subunit alpha, mitochondrial	1.111×10^{9}	2.814×10^{8}	3.949
P01029	C4b	Complement C4-B	8.334×10^{8}	2.111×10^{8}	3.948
Q14B21	Mrpl9	39S ribosomal protein L9, mitochondrial	5.759×10^{7}	1.470×10^{7}	3.917
A2AMW0	Capzb	F-actin-capping protein subunit beta	1.374×10^{8}	3.527×10^{7}	3.895
Q3TR93	Decr2	Uncharacterized protein	4.438×10^{7}	1.158×10^{7}	3.832
P14115	Rpl27a	60S ribosomal protein L27a	1.710×10^{8}	4.513×10^{7}	3.790
Q61495	Dsg1a	Desmoglein-1-alpha	4.736×10^{8}	1.252×10^{8}	3.782
P35700	Prdx1	Peroxiredoxin-1	3.370×10^{8}	8.911×10^{7}	3.782
Q8R0I8	Cfhr2	BC026782 protein	1.080×10^{9}	2.858×10^{8}	3.777
Q9DBG1	Cyp27a1	Sterol 26-hydroxylase, mitochondrial	2.111×10^{8}	5.618×10^{7}	3.757
Q3U861	Atp6v1d	V-type proton ATPase subunit D	2.851×10^{8}	7.666×10^{7}	3.719
P62830	Rpl23	60S ribosomal protein L23	1.217×10^{8}	3.293×10^{7}	3.697
F8VPR2	Fmnl2	Formin-like protein 2	2.802×10^{7}	7.593×10^{6}	3.690

Uniprot accession	Protein	Description	Abundance		CPS23F: CPS8 fold
			CPS23F	CPS8	
P45952	Acadm	Medium-chain specific acyl-CoA dehydrogenase, mitochondrial	9.905×10^{7}	2.709×10^{7}	3.657
Q3TKR5	Rpl5	Ribosomal protein L5	1.696×10^{8}	4.662×10^{7}	3.638
P68404	Prkcb	Protein kinase C beta type	3.678×10^{7}	1.018×10^{7}	3.615
P33267	Cyp2f2	Cytochrome P450 2F2	7.924×10^{7}	2.196×10^{7}	3.609
Q00PI9	Hnrnpul2	Heterogeneous nuclear ribonucleoprotein U-like protein 2	1.743×10^{8}	4.848×10^{7}	3.595
Q5SVF7	Nipsnap1	Uncharacterized protein	1.508×10^{10}	4.250×10^{9}	3.548
Q5EBQ6	Rpl9	60S ribosomal protein L9	8.030×10^{8}	2.282×10^{8}	3.519
Q53ZD4	Mgst1	Glutathione transferase	6.168×10^{8}	1.763×10^{8}	3.500
O89054	Actb	Cytoskeletal beta-actin (Fragment)	9.044×10^{8}	2.587×10^{8}	3.496
Q8BH80	Vapb	Vesicle-associated membrane protein, associated protein B and C	8.400×10^{7}	2.421×10^{7}	3.469
Q9CPQ8	Atp5mg	ATP synthase subunit g, mitochondrial	1.978×10^{9}	5.704×10^{8}	3.468
Q99M74	Krt82	Keratin, type II cuticular Hb2	1.470×10^{10}	4.312×10^{9}	3.409
Q3TD78	Nipsnap2	NIPSNAP domain-containing protein	3.235×10^{8}	9.673×10^{7}	3.344
Q9WV55	Vapa	Vesicle-associated membrane protein-associated protein A	1.077×10^{8}	3.244×10^{7}	3.321
P97429	Anxa4	Annexin A4	2.395×10^{8}	7.443×10^{7}	3.217
Q545A2	Slc25a5	ADP/ATP translocase	1.858×10^{9}	5.871×10^{8}	3.164
Q91XB2	Mrpl3	Mitochondrial ribosomal protein L3	1.118×10^{8}	3.564×10^{7}	3.136
Q8JZU2	Slc25a1	Tricarboxylate transport protein, mitochondrial	1.205×10^{8}	3.843×10^{7}	3.136
Q99LC3	Ndufa10	NADH dehydrogenase [ubiquinone] 1 alpha subcomplex subunit 10, mitochondrial	4.595×10^{7}	1.466×10^{7}	3.135
Q8C166	Cpne1	Copine-1	7.679×10^{8}	2.451×10^{8}	3.133
A0A668KLU9	Cfhr2	Complement factor H-related 2	9.925×10^{8}	3.177×10^{8}	3.124
P54869	Hmgcs2	Hydroxymethylglutaryl-CoA synthase, mitochondrial	1.129×10^{8}	3.620×10^{7}	3.118

Uniprot accession	Protein	Description	Abundance		CPS23F: CPS8 fold
			CPS23F	CPS8	
Q8BH95	Echs1	Enoyl-CoA hydratase, mitochondrial	4.340×10^{7}	1.397×10^{7}	3.107
Q3U6S1	Vim	Vimentin	2.779×10^{8}	8.996×10^{7}	3.089
Q5I0W0	Atp5pb	ATP synthase F(0) complex subunit B1, mitochondrial	2.176×10^{8}	7.054×10^{7}	3.085
Q9R092	Hsd17b6	17-beta-hydroxysteroid dehydrogenase type 6	5.621×10^{7}	1.851×10^{7}	3.037
O88451	Rdh7	Retinol dehydrogenase 7	9.450×10^{7}	3.118×10^{7}	3.031
A0A0R3P9C8	Ndufa9	NADH dehydrogenase 1 alpha subcomplex subunit 9, mitochondrial	3.467×10^{8}	1.147×10^{8}	3.024
Q4FJU3	Crip2	Crip2 protein	2.104×10^{8}	6.971×10^{7}	3.019
Q3UGS1	Nipsnap1	NIPSNAP domain-containing protein	7.062×10^{9}	2.360×10^{9}	2.992
Q5J7N1	Kras	Kras protein	1.479×10^{8}	4.963×10^{7}	2.979
Q0VDV7	Kras	Kras protein	1.479×10^{8}	4.963×10^{7}	2.979
Q3THU8	Slc25a3	Uncharacterized protein	2.713×10^{8}	9.160×10^{7}	2.962
Q80XN0	Bdh1	D-beta-hydroxybutyrate dehydrogenase, mitochondrial	2.267×10^{9}	7.723×10^{8}	2.936
O88844	Idh1	Isocitrate dehydrogenase [NADP] cytoplasmic	5.674×10^{7}	1.936×10^{7}	2.932
Q9R0X4	Acot9	Acyl-coenzyme A thioesterase 9, mitochondrial	7.331×10^{7}	2.514×10^{7}	2.915
A2A513	Krt10	Keratin, type I cytoskeletal 10	7.987×10^{10}	2.746×10^{10}	2.909
Q3UA17	Mtch2	Uncharacterized protein	1.390×10^{8}	4.801×10^{7}	2.896
P67778	Phb	Prohibitin	3.483×10^{8}	1.203×10^{8}	2.895
P51175	Ppox	Protoporphyrinogen oxidase	1.452×10^{8}	5.026×10^{7}	2.890
P21956-2	Mfge8	Isoform 2 of Lactadherin	9.647×10^{7}	3.364×10^{7}	2.868
A0A075B5M7	Igkv5-39	Immunoglobulin kappa variable 5-39	1.144×10^{8}	4.033×10^{7}	2.837
Q9QZD8	Slc25a10	Mitochondrial dicarboxylate carrier	1.062×10^{8}	3.747×10^{7}	2.833
P99028	Uqcrh	Cytochrome b-c1 complex subunit 6, mitochondrial	3.466×10^{8}	1.230×10^{8}	2.817
Q3U9P0	Rps10	S10_plectin domain-containing protein	2.166×10^{8}	7.690×10^{7}	2.816
Q5M9L7	Rps17	40S ribosomal protein S17	6.270×10^{8}	2.257×10^{8}	2.778

Uniprot accession	Protein	Description	Abundance		CPS23F: CPS8 fold
			CPS23F	CPS8	
Q9D0M3-2	Cyc1	Isoform 2 of Cytochrome c1, heme protein, mitochondrial	7.092×10^{9}	2.562×10^{9}	2.768
Q9ESW4	Agk	Acylglycerol kinase, mitochondrial	1.811×10^{8}	6.576×10^{7}	2.754
Q3TS44	Psma1	Proteasome subunit alpha type	6.687×10^{7}	2.432×10^{7}	2.750
Q8C2Q8	Atp5c1	ATP synthase subunit gamma	1.649×10^{10}	6.002×10^{9}	2.748
A2AKU9	Atp5c1	ATP synthase subunit gamma	1.649×10^{10}	6.002×10^{9}	2.748
Q76LB8	Rdh9	Cis-retinol/androgen dehydrogenase type 3	5.394×10^{7}	1.965×10^{7}	2.745
A1E2B8	NA	Inducible heat shock protein 70	2.428×10^{8}	8.915×10^{7}	2.723
P16054	Prkce	Protein kinase C epsilon type	1.291×10^{8}	4.779×10^{7}	2.702
P06684	C5	Complement C5	2.392×10^{8}	8.867×10^{7}	2.698
Q9DCT2	Ndufs3	NADH dehydrogenase iron-sulfur protein 3, mitochondrial	3.823×10^{7}	1.426×10^{7}	2.681
Q06185	Atp5me	ATP synthase subunit e, mitochondrial	5.504×10^{8}	2.055×10^{8}	2.678
P35550	Fbl	rRNA 2'-O-methyltransferase fibrillarin	2.567×10^{7}	9.684×10^{6}	2.650
Q8BH04	Pck2	Phosphoenolpyruvate carboxykinase [GTP], mitochondrial	2.699×10^{7}	1.022×10^{7}	2.642
Q3V340	Adap2	Uncharacterized protein	5.671×10^{7}	2.160×10^{7}	2.626
Q545F8	Rps4x	40S ribosomal protein S4	5.649×10^{8}	2.152×10^{8}	2.625
Z4YKT6	Dhrs7b	Dehydrogenase/reductase SDR family member 7B	4.303×10^{7}	1.642×10^{7}	2.620
Q5FWI9	Ap2m1	AP-2 complex subunit mu	1.347×10^{8}	5.164×10^{7}	2.608
Q3TWV4	Ap2m1	AP-2 complex subunit mu	1.347×10^{8}	5.164×10^{7}	2.608
Q9DB77	Uqcrc2	Cytochrome b-c1 complex subunit 2, mitochondrial	2.965×10^{8}	1.142×10^{8}	2.596
Q3TIQ2	Rpl12	60S ribosomal protein L12	2.400×10^{8}	9.251×10^{7}	2.595
Q3UJ34	Ass1	Argininosuccinate synthase	8.799×10^{7}	3.394×10^{7}	2.593
G5E8R3	Pcx	Pyruvate carboxylase	4.843×10^{7}	1.906×10^{7}	2.541
Q3TCQ3	Pcx	Pyruvate carboxylase	4.843×10^{7}	1.906×10^{7}	2.541
Q80X85	Mrps7	28S ribosomal protein S7, mitochondrial	2.671×10^{8}	1.054×10^{8}	2.534

Uniprot accession	Protein	Description	Abundance		CPS23F: CPS8 fold
			CPS23F	CPS8	
B1ARA3	Rpl26	60S ribosomal protein L26 (Fragment)	1.186×10^8	4.697×10^7	2.525
A0A0R4IZZ5	Clec4f	C-type lectin domain family 4 member F	7.238×10^7	2.867×10^7	2.524
A8DUP7	Hbbt1	Beta-globin	3.803×10^9	1.527×10^9	2.491
P48962	Slc25a4	ADP/ATP translocase 1	1.457×10^9	5.871×10^8	2.481
P49718	Mcm5	DNA replication licensing factor MCM5	3.651×10^7	1.473×10^7	2.479
P20444	Prkca	Protein kinase C alpha type	3.349×10^7	1.356×10^7	2.470
Q542G9	Anxa2	Annexin	3.538×10^8	1.441×10^8	2.455
Q8VCL2	Sco2	Protein SCO2 homolog, mitochondrial	2.508×10^8	1.031×10^8	2.433
Q91X77	Cyp2c50	Cytochrome P450 2C50	2.202×10^8	9.057×10^7	2.432
P56654	Cyp2c37	Cytochrome P450 2C37	2.202×10^8	9.057×10^7	2.432
Q6XVG2	Cyp2c54	Cytochrome P450 2C54	2.202×10^8	9.057×10^7	2.432
Q99JR1	Sfxn1	Sideroflexin-1	5.253×10^7	2.162×10^7	2.430
Q3U0S6	Rasip1	Ras-interacting protein 1	5.526×10^8	2.278×10^8	2.426
P54071	Idh2	Isocitrate dehydrogenase [NADP], mitochondrial	7.282×10^8	3.014×10^8	2.416
P47963	Rpl13	60S ribosomal protein L13	2.375×10^8	9.841×10^7	2.414
A0A0B4J1J2	Igkv5-43	Immunoglobulin kappa chain variable 5-43 (Fragment)	1.603×10^8	6.665×10^7	2.405
P01642	Gm10881	Ig kappa chain V-V region L7 (Fragment)	1.603×10^8	6.665×10^7	2.405
Q5SX53	Slc25a11	Uncharacterized protein	1.890×10^8	7.904×10^7	2.391
Q3UKH3	Acaa2	Uncharacterized protein	1.147×10^9	4.818×10^8	2.380
Q99JY0	Hadhb	Trifunctional enzyme subunit beta, mitochondrial	1.414×10^9	5.961×10^8	2.372
P11928	Oas1a	2'-5'-oligoadenylate synthase 1A	8.029×10^7	3.404×10^7	2.359
P28843	Dpp4	Dipeptidyl peptidase 4	2.092×10^8	8.911×10^7	2.348
P43024	Cox6a1	Cytochrome c oxidase subunit 6A1, mitochondrial	2.011×10^9	8.584×10^8	2.343
Q9D023	Mpc2	Mitochondrial pyruvate carrier 2	1.475×10^8	6.353×10^7	2.322
Q99N15	Hsd17b10	17beta-hydroxysteroid dehydrogenase type 10/short chain L-3-hydroxyacyl-CoA dehydrogenas	7.508×10^7	3.240×10^7	2.318
Q54AH9	Hbb-b2	Beta-2-globin (Fragment)	2.972×10^9	1.292×10^9	2.300

Uniprot accession	Protein	Description	Abundance		CPS23F: CPS8 fold
			CPS23F	CPS8	
A8DUM2	Hbbt1	Beta-globin	3.803×10^{9}	1.659×10^{9}	2.292
Q9ERI6	Rdh14	Retinol dehydrogenase 14	6.549×10^{7}	2.892×10^{7}	2.264
Q9DBM2	Ehhadh	Peroxisomal bifunctional enzyme	3.280×10^{8}	1.458×10^{8}	2.249
Q8VEH7	Oas1g	2'-5' oligoadenylate synthase	7.654×10^{7}	3.404×10^{7}	2.249
B1Q450	HBB1	Hemoglobin beta chain subunit	4.079×10^{9}	1.819×10^{9}	2.242
J3QNY6	Abcb11	Bile salt export pump	5.077×10^{7}	2.265×10^{7}	2.241
Q9DB20	Atp5po	ATP synthase subunit O, mitochondrial	1.995×10^{9}	8.969×10^{8}	2.225
D3YUT3	Rps19	40S ribosomal protein S19 (Fragment)	4.018×10^{8}	1.807×10^{8}	2.224
Q549A5	Clu	Clusterin	3.740×10^{9}	1.685×10^{9}	2.219
Q3TVJ8	Ssr4	Signal sequence receptor subunit delta	9.117×10^{7}	4.145×10^{7}	2.200
Q2XU92	Acsbg2	Long-chain-fatty-acid--CoA ligase ACSBG2	2.468×10^{9}	1.123×10^{9}	2.198
P56480	Atp5f1b	ATP synthase subunit beta, mitochondrial	1.943×10^{8}	8.902×10^{7}	2.183
Q8VDD5	Myh9	Myosin-9	1.011×10^{8}	4.639×10^{7}	2.179
Q9Z0X1	Aifm1	Apoptosis-inducing factor 1, mitochondrial	3.229×10^{7}	1.489×10^{7}	2.169
Q3V1K9	Des	IF rod domain-containing protein	1.083×10^{8}	5.020×10^{7}	2.157
Q3TTN3	Vdac3	Voltage-dependent anion-selective channel protein 3	2.530×10^{8}	1.180×10^{8}	2.145
Q9QUK9	Try5	TESP4	7.629×10^{10}	3.594×10^{10}	2.123
E9Q986	Ctnnd1	Catenin delta-1	1.669×10^{8}	7.952×10^{7}	2.099
Q8CB17	Fetub	Uncharacterized protein	1.365×10^{8}	6.507×10^{7}	2.098
A8DUK2	Hbbt1	Beta-globin	5.656×10^{9}	2.700×10^{9}	2.095
D3YUP5	Exoc3l2	Exocyst complex component 3-like 2	1.210×10^{8}	5.804×10^{7}	2.084
Q3UEK1	Mbl2	Mannan-binding protein	1.947×10^{9}	9.353×10^{8}	2.082
A0A068BGR9	Ndufa7	Complex I-B14.5a	1.158×10^{8}	5.571×10^{7}	2.078
Q8BMD8	Slc25a24	Calcium-binding mitochondrial carrier protein SCaMC-1	2.364×10^{8}	1.139×10^{8}	2.075
A0A1L1SQA8	Rps25	40S ribosomal protein S25	4.448×10^{8}	2.144×10^{8}	2.074
Q8BU88	Mrpl22	39S ribosomal protein L22, mitochondrial	1.531×10^{8}	7.389×10^{7}	2.073

Uniprot accession	Protein	Description	Abundance		CPS23F: CPS8 fold
			CPS23F	CPS8	
Q921R2	Rps13	40S ribosomal protein S13	1.092×10^{9}	5.272×10^{8}	2.071
Q9R0Z4	Ehhadh	L-specific multifunctional beta-oxdiation protein	1.791×10^{8}	8.659×10^{7}	2.069
P21614	Gc	Vitamin D-binding protein	3.846×10^{8}	1.865×10^{8}	2.062
Q9D8L4	NA	Uncharacterized protein	3.695×10^{8}	1.793×10^{8}	2.061
A0A075B5N3	Igkv8-28	Immunoglobulin kappa variable 8-28	1.757×10^{9}	8.629×10^{8}	2.036
Q9WTI7	Myo1c	Unconventional myosin-Ic	1.605×10^{9}	7.945×10^{8}	2.020
A0A4V6JA63	Ighg2b	IgG2b (Fragment)	4.208×10^{8}	2.083×10^{8}	2.020
Q7M754	Gm5409	Try10-like trypsinogen	1.085×10^{9}	5.378×10^{8}	2.018
Q91VT4	Cbr4	Carbonyl reductase family member 4	7.887×10^{8}	3.913×10^{8}	2.016
P62843	Rps15	40S ribosomal protein S15	1.353×10^{8}	6.757×10^{7}	2.003
P54310	Lipe	Hormone-sensitive lipase	7.734×10^{7}	3.867×10^{7}	2.000

Proteins were considered as CPS23F receptor candidates once meeting following criteria:
1. Annotated as receptor or binding proteins;
2. CPS23F:CPS8 enrichment fold ≥ 2.000.
3. Membrane protein-enriched fraction of mouse liver nonparenchymal cells (NPCs) in the presence of 10% mouse serum

Proteins were ranked according to the protein abundance in CPS23F group.
NA, not accessible.

Appendix Table S2. CPS23F-binding proteins from mouse KC

Uniprot accession	Protein	Description	Abundance		CPS23F: CPS8 fold
			CPS23F	CPS8	
Q8BQ07	R3hdm1	Uncharacterized protein	2.367×10^8	0	∞
Q9JHI5	Ivd	Isovaleryl-CoA dehydrogenase, mitochondrial	2.610×10^7	0	∞
O08807	Prdx4	Peroxiredoxin-4	1.936×10^8	1.590×10^8	12.175
Q542G9	Anxa2	Annexin	7.142×10^7	1.176×10^7	6.0743
Q80ZS3	Mrps26	Small ribosomal subunit protein mS26	1.231×10^8	2.448×10^7	5.026
Q3UKC8	Gng5	Guanine nucleotide-binding protein subunit gamma	2.928×10^8	8.710×10^7	3.362
F8WIB1	Arl1	ADP-ribosylation factor-like 1	5.150×10^7	1.700×10^7	3.030
Q9D0L7-2	Armc10	Armadillo repeat-containing protein 10	2.818×10^7	9.323×10^6	3.023
Q05DV1	Por	NADPH--hemoprotein reductase	1.015×10^8	3.456×10^7	2.938
Q99M74	Krt82	Keratin, type II cuticular Hb2	1.596×10^9	5.755E8	2.774
E9PVG8	Fcgbpl1	Fc fragment of IgG binding protein like 1	8.450×10^9	3.130×10^9	2.700
Q8R4R6	Nup35	Nucleoporin NUP35	5.947×10^7	2.207×10^7	2.694
B9EKL6	Ptp4a1	Protein tyrosine phosphatase 4a1	6.353×10^7	2.379×10^7	2.670
P50518	Atp6v1e1	V-type proton ATPase subunit E 1	5.170×10^7	1.936×10^7	2.700
P50516	Atp6v1a	V-type proton ATPase catalytic subunit A	5.356×10^7	2.143×10^7	2.499
Q3UAD6	Hsp90b1	Histidine kinase/HSP90-like ATPase domain-containing protein	6.409×10^7	2.567×10^7	2.497
Q9CQB4	Uqcrb	Cytochrome b-c1 complex subunit 7	4.539×10^8	1.929×10^8	2.353
H3BJU7	Arhgef2	Rho guanine nucleotide exchange factor 2	1.266×10^7	5.404×10^6	2.343
Q4FJK0	Decr1	Decr1 protein	4.080×10^7	1.748×10^7	2.334
P27048	Snrpb	Small nuclear ribonucleoprotein-associated protein B	5.507×10^7	2.381×10^7	2.313
Q8VD75	Hip1	Huntingtin-interacting protein 1	2.081×10^7	9.008×10^6	2.310

Uniprot accession	Protein	Description	Abundance		CPS23F: CPS8 fold
			CPS23F	CPS8	
Q8JZN5	Acad9	Complex I assembly factor ACAD9, mitochondrial	4.524×10^8	1.969×10^8	2.298
P52843	Sult2a1	Sulfotransferase 2A1	4.834×10^7	2.107×10^7	2.295
Q544Y7	Cfl1	Cofilin 1, non-muscle	4.205×10^7	1.841×10^7	2.284
P39688	Fyn	Tyrosine-protein kinase Fyn	1.073×10^8	4.825×10^7	2.223
Q6P5H2-2	Nes	Nestin	2.153×10^9	9.746×10^8	2.209
Q3UAS2	Capza1	F-actin-capping protein subunit alpha	8.479×10^7	3.842×10^7	2.207
Q3TJ21	Pycr2	Pyrroline-5-carboxylate reductase	1.258×10^8	5.707×10^7	2.204
A0A1B0GR60	Ftl1	Ferritin	7.441×10^7	3.384×10^7	2.199
O70569	rps14	Small ribosomal subunit protein uS11	9.224×10^7	4.208×10^7	2.192
P62267	Rps23	Small ribosomal subunit protein uS12	1.341×10^8	6.279×10^7	2.136
Q8R2Y8	Ptrh2	Peptidyl-tRNA hydrolase 2, mitochondrial	4.575×10^7	2.149×10^7	2.129
Q5SQB0	Npm1	Nucleophosmin	7.924×10^7	3.782×10^7	2.095
Q3UJ76	Ap2s1	AP complex subunit sigma	9.174×10^7	4.391×10^7	2.090
Q8VDP4	Ccar2	Cell cycle and apoptosis regulator protein 2	4.077×10^7	1.952×10^7	2.089
Q5M9J8	Rpl28	Large ribosomal subunit protein eL28	1.037×10^8	5.052×10^7	2.053
O08599	Stxbp1	Syntaxin-binding protein 1	2.616×10^7	1.288×10^7	2.031
Q9WVM8	Aadat	Kynurenine/alpha-aminoadipate aminotransferase, mitochondrial	2.696×10^7	1.346×10^7	2.002
Q8R1S0	Coq6	Ubiquinone biosynthesis monooxygenase COQ6, mitochondrial	3.301×10^7	1.649×10^7	2.002
Q9CYD3	Crtap	Cartilage-associated protein	4.978×10^7	2.492×10^7	1.997

Proteins were considered as CPS23F receptor candidates once meeting following criteria:
1. Annotated as receptor or binding proteins;
2. CPS23F:CPS8 enrichment fold ≥ 2.000.
3. Membrane protein-enriched fraction of mouse liver nonparenchymal cells (NPCs) without mouse serum
Proteins were ranked according to the protein abundance in CPS23F group.
NA, not accessible.

Appendix Table S3. CPS23F-binding proteins from human serum

Uniprot accession	Protein	Description	Abundance		CPS23F: CPS8 fold
			CPS23F	CPS8	
P02776	PF4	Platelet factor 4	1.211×10^{9}	0	∞
P02741	Crp	C-reactive protein	8.833×10^{8}	0	∞
P12883	MYH7	Myosin-7	7.135×10^{8}	0	∞
P13533	MYH6	Myosin-6	6.685×10^{8}	0	∞
A0A6E1W127	MYH7B	Myosin-7B	4.116×10^{8}	0	∞
A0A590UJU8	MYL2	Myosin regulatory light chain 2, ventricular/cardiac muscle isoform	3.322×10^{8}	0	∞
I3L1K6	MYL4	Myosin light chain 4	3.192×10^{8}	0	∞
P12524	MYCL	Protein L-Myc	2.374×10^{8}	0	∞
Q9UIW2	PLXNA1	Plexin-A1	2.022×10^{8}	0	∞
K7EQL4	TNNT1	Troponin T, slow skeletal muscle	1.343×10^{8}	0	∞
G3V489	TNNI1	Troponin I, slow skeletal muscle	1.308×10^{8}	0	∞
P05976	MYL1	Myosin light chain 1/3, skeletal muscle isoform	1.240×10^{8}	0	∞
P35609	ACTN2	Alpha-actinin-2	1.109×10^{8}	0	∞
A0A075B7D8	IGHV3OR15-7	Immunoglobulin heavy variable 3/OR15-7	9.126×10^{7}	0	∞
E9PJL7	CRYAB	Alpha-crystallin B chain	8.506×10^{7}	0	∞
H3BML9	MYL11	Myosin regulatory light chain 2, skeletal muscle isoform	7.248×10^{7}	0	∞
P06732	CKM	Creatine kinase M-type	6.791×10^{7}	0	∞
P05109	S100A8	Protein S100-A8	6.667×10^{7}	0	∞
P68363	TUBA1B	Tubulin alpha-1B chain	5.891×10^{7}	0	∞
P63316	TNNC1	Troponin C, slow skeletal and cardiac muscles	5.037×10^{7}	0	∞
P68366	TUBA4A	Tubulin alpha-4A chain	5.014×10^{7}	0	∞
P02745	C1QA	Complement C1q subcomponent subunit	4.815×10^{7}	0	∞
A0A2R2Y2Q3	TPM3	Tropomyosin 3 nu	4.514×10^{7}	0	∞
G3V1V7	MYBPC1	Myosin binding protein C, slow type, isoform CRA_e	4.461×10^{7}	0	∞

Uniprot accession	Protein	Description	Abundance		CPS23F: CPS8 fold
			CPS23F	CPS8	
P20851	C4BPB	C4b-binding protein beta chain	4.287×10^7	0	∞
Q13103	SPP2	Secreted phosphoprotein 24	4.226×10^7	0	∞
H7C5W9	ATP2A2	Endoplasmic reticulum class 1/2 Ca (2+) ATPase	4.151×10^7	0	∞
P04075	ALDOA	Fructose-bisphosphate aldolase A	4.123×10^7	0	∞
A0A6Q8PFK8	HSPB1	Heat shock protein beta-1	3.264×10^7	0	∞
P27105	STOM	Stomatin OS=Homo sapiens	2.986×10^7	0	∞
A0A0A0MS76	ZNF99	Zinc finger protein 99	2.774×10^7	0	∞
P12273	PIP	Prolactin-inducible protein	2.631×10^7	0	∞
H0YKX5	TPM1	Tropomyosin alpha-1 chain	2.542×10^7	0	∞
A0A1X7SC65	HSPB6	Heat shock protein beta-6	2.386×10^7	0	∞
A0A0C4DH36	IGHV3-38	Probable non-functional immunoglobulin heavy variable 3-38	2.348×10^7	0	∞
P11217	PYGM	Glycogen phosphorylase, muscle form	2.330×10^7	0	∞
P06727	APOA4	Apolipoprotein A-IV	2.289×10^7	0	∞
E5RGZ4	ENO3	2-phospho-D-glycerate hydro-lyase	2.133×10^7	0	∞
Q14315	FLNC	Filamin-C OS=Homo sapiens	2.071×10^7	0	∞
P06733	ENO1	Alpha-enolase OS=Homo sapiens	1.910×10^7	0	∞
A0A0G2JL69	C2	C3/C5 convertase	1.679×10^7	0	∞
Q8WZ42	TTN	Titin OS=Homo sapiens	1.506×10^7	0	∞
A0A3B3ISR2	C1R	Complement subcomponent C1r	1.319×10^7	0	∞
P25705	ATP5F1A	ATP synthase subunit alpha, mitochondrial	4.854×10^8	3.279×10^6	148.017
Q6KB66	KRT80	Keratin, type II cytoskeletal 80	4.573×10^8	1.081×10^7	42.301
H0YF91	HYDIN	Hydrocephalus-inducing protein homolog	1.584×10^9	4.293×10^7	36.884
A0A0A0MSG9	SUN3	SUN domain-containing protein 3	1.334×10^8	7.284×10^6	18.308

Uniprot accession	Protein	Description	Abundance		CPS23F: CPS8 fold
			CPS23F	CPS8	
P02748	C9	Complement component C9	1.206×10^{10}	8.664×10^{8}	13.918
P07360	C8G	Complement component C8 gamma chain	2.327×10^{9}	1.720×10^{8}	13.527
Q9H2G4	TSPYL2	Testis-specific Y-encoded-like protein 2	3.146×10^{9}	2.627×10^{7}	11.974
P07358	C8B	Complement component C8 beta chain	2.066×10^{9}	1.771×10^{8}	11.662
P07357	C8A	Complement component C8 alpha chain	3.728×10^{9}	3.352×10^{8}	11.122
E9PJV1	GLYATL1	Glycine N-acyltransferase-like protein 1	1.299×10^{9}	1.302×10^{8}	9.977
P01031	C5	Complement C5	3.468×10^{9}	3.852×10^{8}	9.01
P13671	C6	Complement component C6	2.071×10^{9}	2.402×10^{8}	8.623
P10643	C7	Complement component C7	2.001×10^{9}	2.373×10^{8}	8.43
C9JGI8	PALS2	MAGUK p55 subfamily member 6	2.038×10^{8}	2.560×10^{7}	7.961
A0A140TA32	C4A	C4a anaphylatoxin	4.151×10^{9}	5.320×10^{8}	7.802
A0A140TA29	C4B	C4a anaphylatoxin	4.151×10^{9}	5.320×10^{8}	7.802
P36980	CFHR2	Complement factor H-related protein 2	1.512×10^{9}	1.985×10^{8}	7.617
P06702	S100A9	Protein S100-A9	1.044×10^{8}	1.384×10^{7}	7.541
Q03591	CFHR1	Complement factor H-related protein 1	2.790×10^{9}	3.726×10^{8}	7.488
P01024	C3	Complement C3	2.253×10^{11}	3.149×10^{10}	7.154
A0A0G2JPR0	C4A	C4a anaphylatoxin	3.709×10^{9}	5.320×10^{8}	6.971
P12814	ACTN1	Alpha-actinin-1	5.768×10^{7}	8.496E6	6.789
P68133	ACTA1	Actin, alpha skeletal muscle	2.460×10^{9}	4.121×10^{8}	5.969
Q9BXR6	CFHR5	Complement factor H-related protein 5	4.829×10^{9}	8.661×10^{8}	5.576
P02671	FGA	Fibrinogen alpha chain	6.017×10^{8}	1.094×10^{8}	5.502
P01599	IGKV1-17	Immunoglobulin kappa variable 1-17	1.058×10^{8}	1.978×10^{7}	5.350

Uniprot accession	Protein	Description	Abundance		CPS23F: CPS8 fold
			CPS23F	CPS8	
O14791	APOL1	Apolipoprotein L1	1.524×10^{8}	3.320×10^{7}	4.590
Q92496	CFHR4	Complement factor H-related protein 4	5.265×10^{7}	9.962×10^{6}	5.285
P04003	C4BPA	C4b-binding protein alpha chain	1.950×10^{9}	4.296×10^{8}	4.540
G5E9F8	PROS1	Vitamin K-dependent protein S	9.908×10^{7}	2.214×10^{7}	4.475
P07477	PRSS1	Trypsin-1	1.365×10^{8}	3.058×10^{7}	4.464
P02743	APCS	Serum amyloid P-component	8.399×10^{9}	1.900×10^{9}	4.421
P27169	PON1	Serum paraoxonase/arylesterase 1	1.090×10^{8}	2.519×10^{7}	4.329
P0CG39	POTEJ	POTE ankyrin domain family member J	8.361×10^{8}	1.963×10^{8}	4.259
P27918	CFP	Properdin	1.878×10^{10}	4.566×10^{9}	4.112
A0A0A0MS15	IGHV3-49	Immunoglobulin heavy variable 3-49	1.796×10^{8}	4.401×10^{7}	4.082
P06312	IGKV4-1	Immunoglobulin kappa variable 4-1	1.781×10^{9}	4.664×10^{8}	3.819
P60709	ACTB	Actin, cytoplasmic 1	1.753×10^{9}	4.630×10^{8}	3.787
P02747	C1QC	Complement C1q subcomponent subunit C	1.414×10^{8}	3.740×10^{7}	3.781
Q562R1	ACTBL2	Beta-actin-like protein 2	1.153×10^{9}	3.107×10^{8}	3.711
P04406	GAPDH	Glyceraldehyde-3-phosphate dehydrogenase	7.976×10^{7}	2.168×10^{7}	3.679
A0A0A0MRZ8	IGKV3D-11	Immunoglobulin kappa variable 3D-11	5.353×10^{9}	1.489×10^{9}	3.595
P05089	ARG1	Arginase-1 OS=Homo sapiens	1.918×10^{7}	5.736×10^{6}	3.343
A0A0J9YX35	IGHV3-64D	Immunoglobulin heavy variable 3-64D	3.812×10^{8}	1.251×10^{8}	3.046
Q96IY4	CPB2	Carboxypeptidase B2	5.950×10^{7}	1.991×10^{7}	2.988
P59665	DEFA1; DEFA1B	Neutrophil defensin 1	7.638×10^{7}	2.613×10^{7}	2.924
P55056	APOC4	Apolipoprotein C-IV	6.771×10^{9}	2.365×10^{9}	2.863
P02647	APOA1	Apolipoprotein A-I	5.019×10^{8}	1.788×10^{8}	2.807
P02675	FGB	Fibrinogen beta chain	5.329×10^{8}	1.931×10^{8}	2.759
A0A075B6I9	IGLV7-46	Immunoglobulin lambda variable 7-46	1.094×10^{8}	4.061×10^{7}	2.694

Uniprot accession	Protein	Description	Abundance		CPS23F: CPS8 fold
			CPS23F	CPS8	
Q9H4B7	TUBB1	Tubulin beta-1 chain	3.513×10^7	1.327 × 107	2.648
P01009	SERPINA1	Alpha-1-antitrypsin	9.661×10^7	3.678×10^7	2.627
P02765	AHSG	Alpha-2-HS-glycoprotein	3.442×10^7	1.366×10^7	2.520
Q02413	DSG1	Desmoglein-1	8.443×10^7	3.388×10^7	2.492
Q13156	RPA4	Replication protein A 30 kDa subunit	4.989×10^8	2.050×10^8	2.434
P49913	CAMP	Cathelicidin antimicrobial peptide	2.239×10^9	9.260×10^8	2.418
P08603	CFH	Complement factor H	8.914×10^8	3.707×10^8	2.405
P01764	IGHV3-23	Immunoglobulin heavy variable 3-23	2.257×10^9	9.898×10^8	2.280
A0A0J9YY99	NA	Ig-like domain-containing protein	2.257×10^9	9.898×10^8	2.280
P04114	APOB	Apolipoprotein B-100	3.842×10^7	1.701×10^7	2.259
A0A0C4DH35	IGHV3-35	Probable non-functional immunoglobulin heavy variable 3-35	1.567×10^9	7.011×10^8	2.235
P01040	CSTA	Cystatin-A	6.798×10^7	3.178×10^7	2.139
P10909	CLU	Clusterin	5.618×10^8	2.672×10^8	2.102
P02751	FN1	Fibronectin	1.272×10^8	6.112×10^7	2.081
P01042	KNG1	Kininogen-1	5.250×10^8	2.559×10^8	2.052
F5H265	UBC	Polyubiquitin-C	9.249×10^7	4.563×10^7	2.027

Proteins were considered as CPS23F receptor candidates once meeting following criteria:
1. Annotated as receptor or binding proteins;
2. CPS23F:CPS8 enrichment fold ≥ 2.000.
Proteins were ranked according to the protein abundance in CPS23F group.
NA, not accessible.

Appendix Table S4. Cryo-EM data processing and refinement statistics

**Data collection and processing**	**CRP/23F complex**
Voltage (kV)	300
Electron exposure ($e^-/Å^2$)	50
Defocus range (μm)	-1.0~-2.0
Pixel size (Å)	0.85
Number of frames collected	32
Micrographs Collected (no.)	8,276
Symmetry imposed	C5
Final particles (no.)	207,101
Map resolution (Å)	2.78
FSC threshold	0.143
**Refinement**	
Initial model used (PDB code)	1B09
Map sharpening methods	DeepEMhancer
Model composition	
Non-hydrogen atoms	8430
Protein residues	1030
Ligands	15
R.m.s. deviations	
Bond lengths (Å)	0.005
Bond angles (°)	1.061
Validation	
MolProbity Score	2.04
Clash Score	6.10
Poor rotamers (%)	3.00
Ramachandran plot	
Favored	95.10
Allowed	4.90
Disallowed	0.00
Model vs. Data	
CC (mask)	0.83
CC (volume)	0.84
CC (peaks)	0.81
Mean ligand CC	0.68

Appendix Table S5. Strains used in this study

Strain ID	Genotype	Capsule type	CT50 in WT mouse (min)	CT50 in *Crp*$^{-/-}$ mouse (min)	Source	Region
S. pneumoniae						
TH15820	TH870Δ*cps*	-	0.99	0.99	-	-
TH15943	TH870Δ*cps*::*cps*8	8	18.4	-	-	-
TH15984	Wild type	9N	2.13	1.7	Sputum	China
TH13133	TH870Δ*cps*::*cps*9V	9V	1.46	2.12	-	-
TH2594	Wild type	10A	1.28	1.06	Blood	China
TH12932	Wild type	11A	1.49	>30	Sputum	China
TH15944	TH870Δ*cps*::*cps*14	14	0.57	0.47	-	-
TH2941	Wild type	15B	0.97	>30	Blood	China
TH886	Wild type	15C	0.9	>30	CSF	USA
TH16784	Wild type	16F	0.79	>30	Sputum	China
TH2734	Wild type	17F	4.59	24.9	Blood	China
TH14190	TH870Δ*cps*::*cps*19A	19A	1.26	2.03	-	-
TH14188	TH870Δ*cps*::*cps*19F	19F	1.12	1.28	-	-
TH2740	Wild type	19F	-	-	-	China
TH2591	Wild type	20B	1.8	28.89	Blood	China
TH16792	Wild type	21	1.32	18.56	Sputum	China
TH16798	Wild type	23A	2.87	>30	Blood	China
TH901	Wild type	23B	0.87	2.06	Blood	USA
TH15945	TH870Δ*cps*::*cps*23F	23F	1.31	>30	-	-
TH17250	Wild type	27	5.35	16.62	Blood	China
TH17366	TH2740Δ*cps*::*cps*23F	33A	9.51	>30	-	-
TH16814	Wild type	34	1.03	1.33	Sputum	China
TH16815	Wild type	35A	1.5	>30	Sputum	China
TH902	Wild type	35B	11.03	>30	CSF	USA
TH16820	Wild type	35C	1.2	>30	Sputum	China
TH16824	Wild type	37	3.79	9.28	Sputum	China
TH16828	Wild type	41A	9.62	11.5	Sputum	China
TH16830	Wild type	48	1.19	1.01	Sputum	China
H. influenzae						
5558 (TH17228)	Wild type	a	0.49	1.7	-	-
M5216 (TH17189)	Wild type	b	1.08	>30	-	USA
TH17229	Wild type	c	0.57	0.38	Sputum	USA
TH17231	Wild type	d	0.38	0.31	Throat	Netherlands
NCTC10479	Wild type	e	>30		-	-

(TH17230)						
TH17232	Wild type	f	0.57	0.61	Throat	China
Strain ID	Genotype	Capsule type	CT50 in WT mouse (min)	CT50 in *Crp*$^{-/-}$ mouse (min)	Source	Region
K. pneumoniae						
TH12849	Wild type	K3	0.27	0.27	Blood	China
TH12880	Wild type	K7	0.43	0.44	Blood	China
TH13089	Wild type	K10	0.28	0.31	Bile	China
TH12838	Wild type	K14	0.41	0.3	Blood	China
TH13012	Wild type	K21	0.24	6.55	Tissue	China
TH12852	Wild type	K23	0.28	0.29	Blood	China
TH13098	Wild type	K27	0.32	0.31	Blood	China
TH13007	Wild type	K57	0.46	0.43	Blood	China
TH12855	Wild type	K60	0.9	0.63	Blood	China
TH12841	Wild type	K64	0.62	1.76	Blood	China
TH12879	Wild type	K124	0.39	0.35	Blood	China

Appendix Table S6. Information of strains with recombinant protein plasmids

Strain ID	Plasmid backbone	Insertion sequence/segment	Application
TH17157	pCMV-chikv-strepII	cDNA of mouse CRP	Overexpression of candidates in HEK293F cells
TH17158	pCMV-chikv-strepII	cDNA of human CRP	
TH17198	pCMV-chikv-strepII	cDNA of E81A mutant mouse CRP	
TH17199	pCMV-chikv-strepII	cDNA of F66A mutant mouse CRP	
TH17200	pCMV-chikv-strepII	cDNA of E81A mutant human CRP	
TH17201	pCMV-chikv-strepII	cDNA of F66A mutant human CRP	
TH17202	pCMV-chikv-strepII	cDNA of T76Y mutant human CRP	
TH17203	pCMV-chikv-strepII	cDNA of G76Y mutant mouse CRP	
TH17388	pCMV-chikv-strepII	cDNA of 71-91 mutant human CRP	

Appendix Table S7. Primers used in this study

Primer ID	Sequence (5’-3’)	Application
Pr19504	GTCGACATGGAGAAGCTACTCTGGTGCCTTC	Amplification of mouse CRP fragment in TH17157
Pr19505	GGATCCTCATTTTTCGAACTGCGGGTGGCTCCAACCTCCCGATCCACCTCCGGACCACAGCTGCGGCTTAATAAAC	
Pr19512	AAAACTGCAGGCCACCATGGAGAAGCTGT	Amplification of human CRP fragment in TH17158
Pr19513	CGCGGATCCTCATTTTTCGAACTG	
Pr19562	GAATCGTACTGCAGCACCACCCACTCCAAAAGTATACTGTTTATCCTTATTCCAAAATATGAGAAT	Construction of E81A mutant mouse CRP with Pr19504/Pr19505 from TH17157
Pr19563	ATTCTCATATTTTGGAATAAGGATAAACAGTATACTTTTGGAGTGGGTGGTGCTGCAGTACGATTC	
Pr19564	GAATCGTACTTCAGCACCACCCACTCCAAAAGTATACTGTTTATCCTTATTCCAAGCTATGAGAAT	Construction of F66A mutant mouse CRP with Pr19504/Pr19505 from TH17157
Pr19565	ATTCTCATAGCTTGGAATAAGGATAAACAGTATACTTTTGGAGTGGGTGGTGCTGAAGTACGATTC	
Pr19566	GAATAATATTGCAGACCCACCCACTGTAAAACTGTATCCTATATCCTTAGACCAAAATATGAGAAT	Construction of E81A mutant human CRP with Pr19512/Pr19513 from TH17158
Pr19567	ATTCTCATATTTTGGTCTAAGGATATAGGATACAGTTTTACAGTGGGTGGGTCTGCAATATTATTC	
Pr19568	GAATAATATTTCAGACCCACCCACTGTAAAACTGTATCCTATATCCTTAGACCAAGCTATGAGAAT	Construction of F66A mutant human CRP with Pr19512/Pr19513 from TH17158
Pr19569	ATTCTCATAGCTTGGTCTAAGGATATAGGATACAGTTTTACAGTGGGTGGGTCTGAAATATTATTC	
Pr19570	GAATAATATTTCAGACCCACCCACGTAAAAACTGTATCCTATATCCTTAGACCAAAATATGAGAAT	Construction of T76Y mutant human CRP with Pr19512/Pr19513 from TH17158
Pr19571	ATTCTCATATTTTGGTCTAAGGATATAGGATACAGTTTTTACGTGGGTGGGTCTGAAATATTATTC	
Pr19572	GAATCGTACTTCAGCACCACCCACGTAAAAAGTATACTGTTTATCCTTATTCCAAAATATGAGAAT	Construction of G76Y mutant mouse CRP with Pr19504/Pr19505 from TH17157
Pr19573	ATTCTCATATTTTGGAATAAGGATAAACAGTATACTTTTTACGTGGGTGGTGCTGAAGTACGATTC	
Pr19933	GGTGCTGAAGTACGATTCATGGTTTCAGAGATTCCTGAGGCTCCAGTACACATTTGTAC	Amplification of human $CRP^{m71\text{-}91}$ in TH17158
Pr19934	TCGTACTTCAGCACCACCCACTCCAAAAGTATACTGTTTATCCTTAGACCAAAATATGA	

Underlined nucleotides indicate restriction enzyme sites
