# Supplementary material for: C-reactive protein is a broad-spectrum capsule-binding receptor for hepatic capture of blood-borne bacteria
Source: EMBO J. 2025 Nov 10;44(24):7364–94. doi: 10.1038/s44318-025-00623-w (PMC12705745; doi:10.1038/s44318-025-00623-w)
Supplement: Supplementary file 12 — Movie EV10 [file 44318_2025_623_MOESM12_ESM.zip › Movie EV10/Movie EV10 legends.docx]

**Movie EV10. Reduced liver capture of serotype-23F *S. pneumoniae* in *C3^-/-^* and CR3/CRIg^-/-^mice.**

Serotype-23F *S. pneumoniae* were labeled with FITC (green) and intravenously inoculated at 5 × 10^7^ CFU for real-time imaging in WT, *C3^-/-^* and CR3/CRIg^-/-^ mice. Kupffer cells (KCs) and liver sinusoidal vasculature were stained with AF647-conjugated anti-F4/80 (red) and AF594-conjugated anti-CD31 (cyan), respectively. Quantitative analysis is presented in Fig. 5E.
