# Supplementary figures and images for: C-reactive protein is a broad-spectrum capsule-binding receptor for hepatic capture of blood-borne bacteria
Source: EMBO J. 2025 Nov 10;44(24):7364–94. doi: 10.1038/s44318-025-00623-w (PMC12705745; doi:10.1038/s44318-025-00623-w)

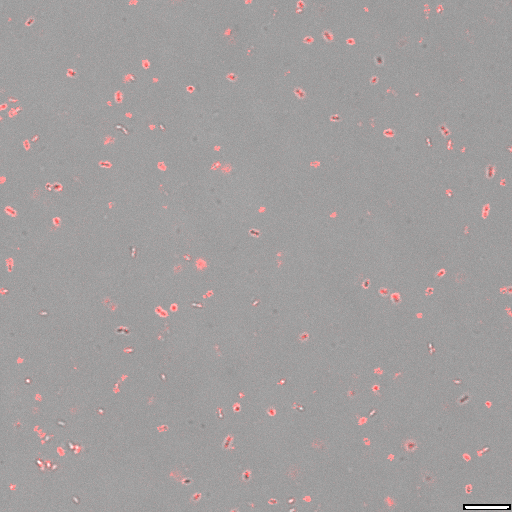

Supplement: Supplementary file 13 — Source data Fig. 1 [file 44318_2025_623_MOESM13_ESM.zip › SD figure 1/Figure 1H/Sp23F_merged.tif]

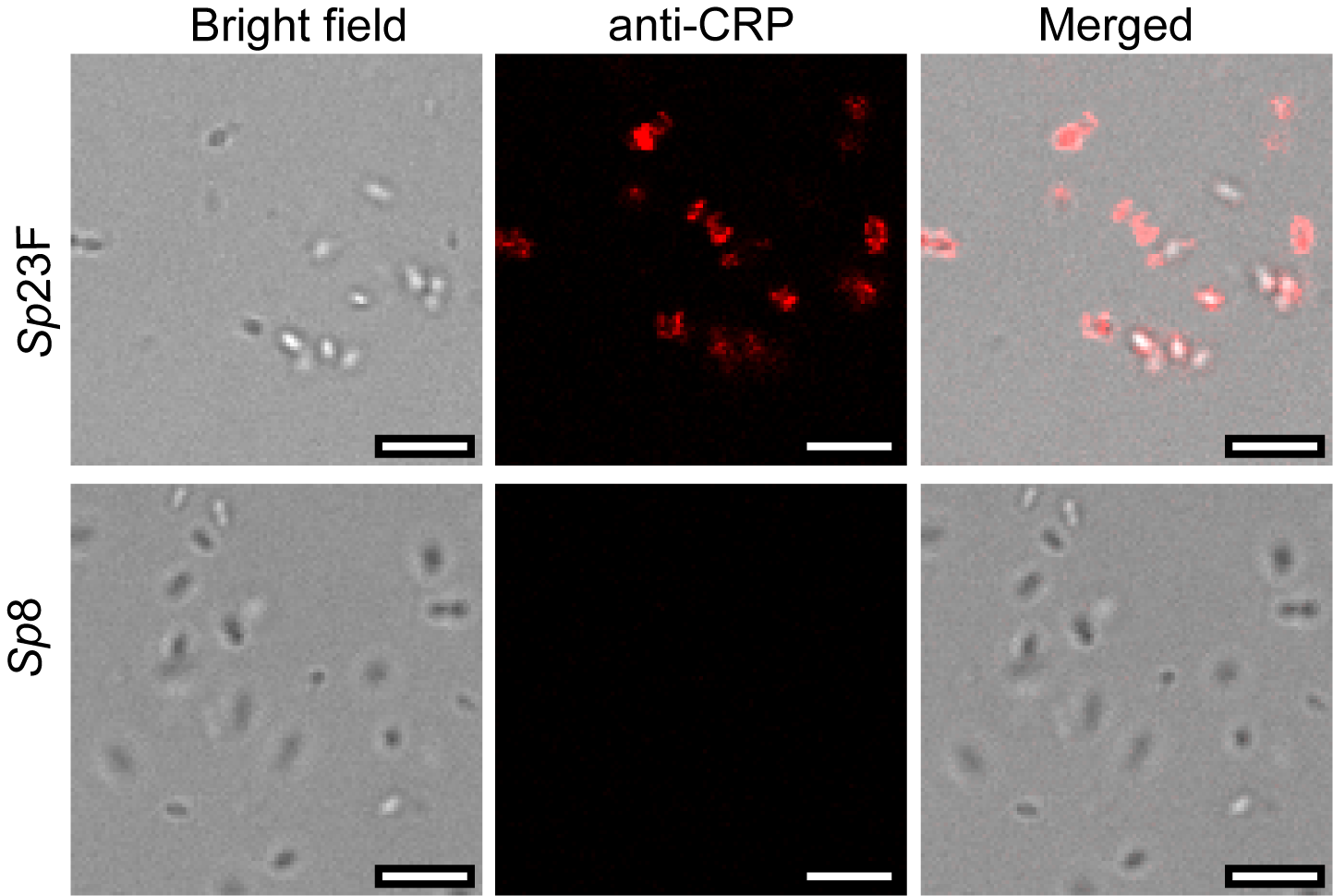

Supplement: Supplementary file 13 — Source data Fig. 1 [file 44318_2025_623_MOESM13_ESM.zip › SD figure 1/Figure 1H/Figure 1H.tif]

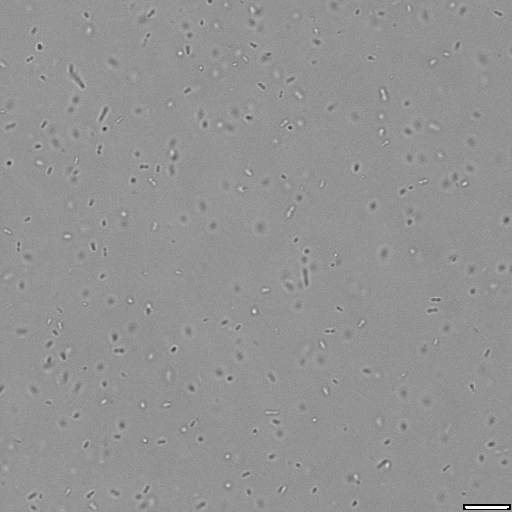

Supplement: Supplementary file 13 — Source data Fig. 1 [file 44318_2025_623_MOESM13_ESM.zip › SD figure 1/Figure 1H/Sp8_Bright field.tif]

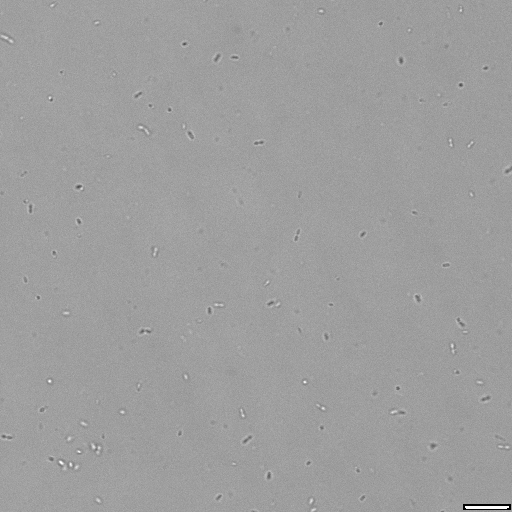

Supplement: Supplementary file 13 — Source data Fig. 1 [file 44318_2025_623_MOESM13_ESM.zip › SD figure 1/Figure 1H/Sp23F_Bright field.tif]

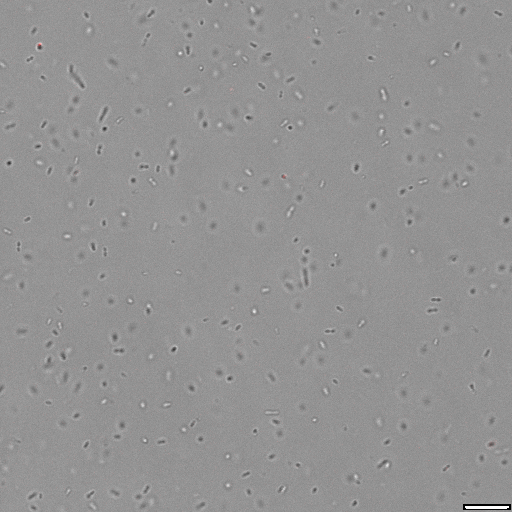

Supplement: Supplementary file 13 — Source data Fig. 1 [file 44318_2025_623_MOESM13_ESM.zip › SD figure 1/Figure 1H/Sp8_merged.tif]

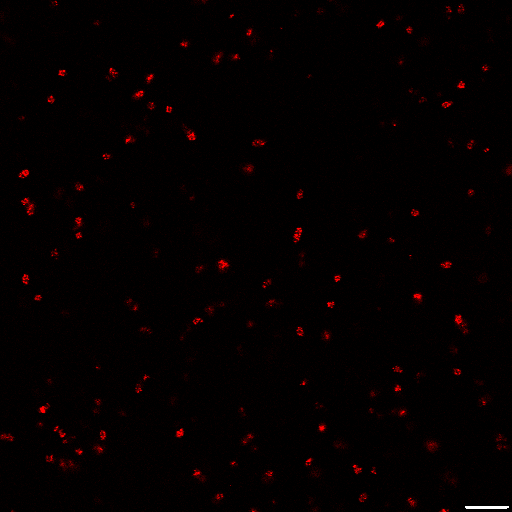

Supplement: Supplementary file 13 — Source data Fig. 1 [file 44318_2025_623_MOESM13_ESM.zip › SD figure 1/Figure 1H/Sp23F_anti-CRP.tif]

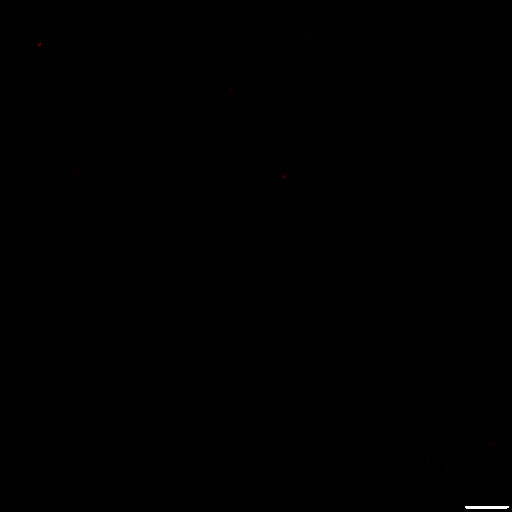

Supplement: Supplementary file 13 — Source data Fig. 1 [file 44318_2025_623_MOESM13_ESM.zip › SD figure 1/Figure 1H/Sp8_anti-CRP.tif]

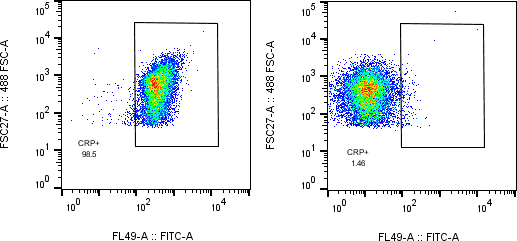

Supplement: Supplementary file 13 — Source data Fig. 1 [file 44318_2025_623_MOESM13_ESM.zip › SD figure 1/Figure 1F/Figure 1F.tiff]

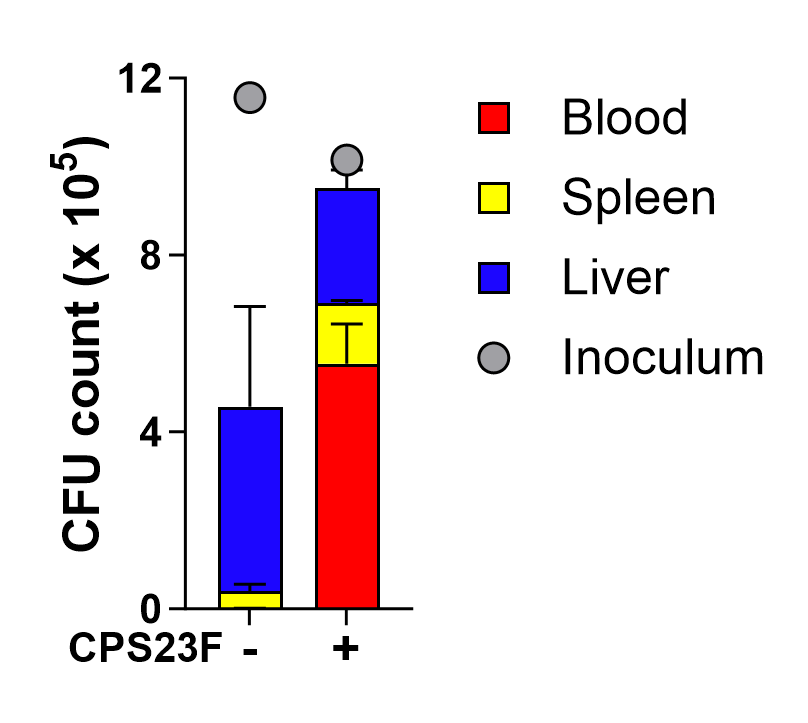

Supplement: Supplementary file 13 — Source data Fig. 1 [file 44318_2025_623_MOESM13_ESM.zip › SD figure 1/Figure 1A/Figure 1A right panel.tif]

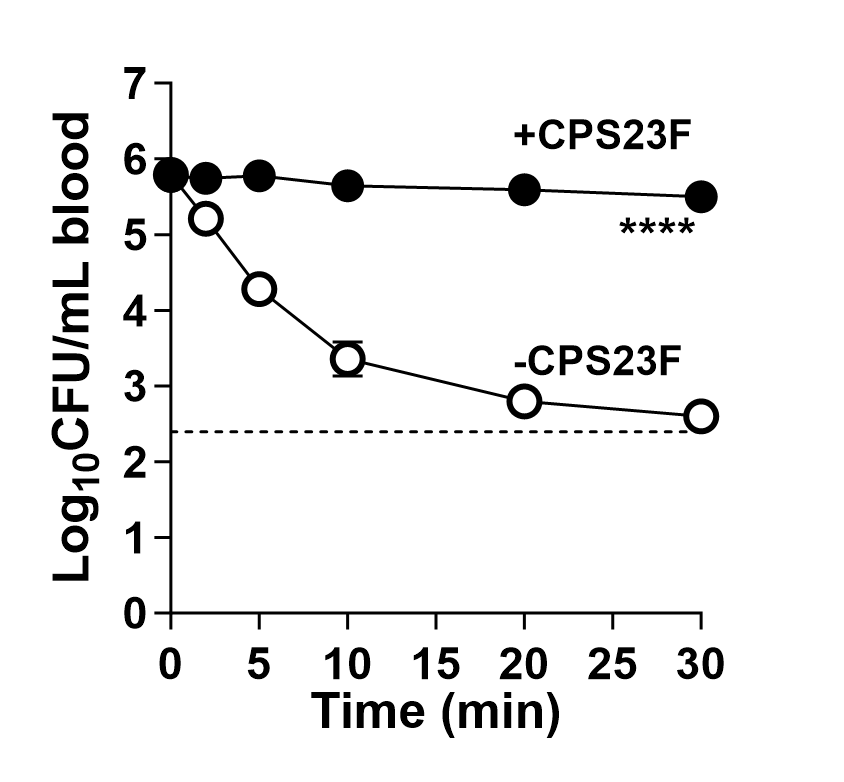

Supplement: Supplementary file 13 — Source data Fig. 1 [file 44318_2025_623_MOESM13_ESM.zip › SD figure 1/Figure 1A/Figure 1A left panel.tif]

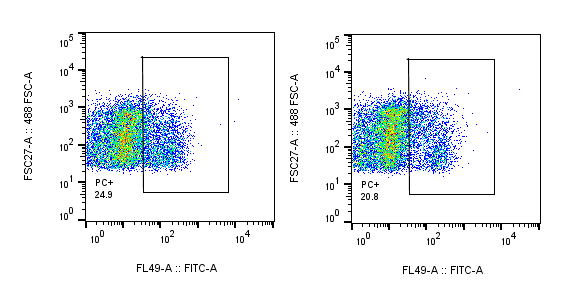

Supplement: Supplementary file 13 — Source data Fig. 1 [file 44318_2025_623_MOESM13_ESM.zip › SD figure 1/Figure 1G/Figure 1G-Layout.tiff]

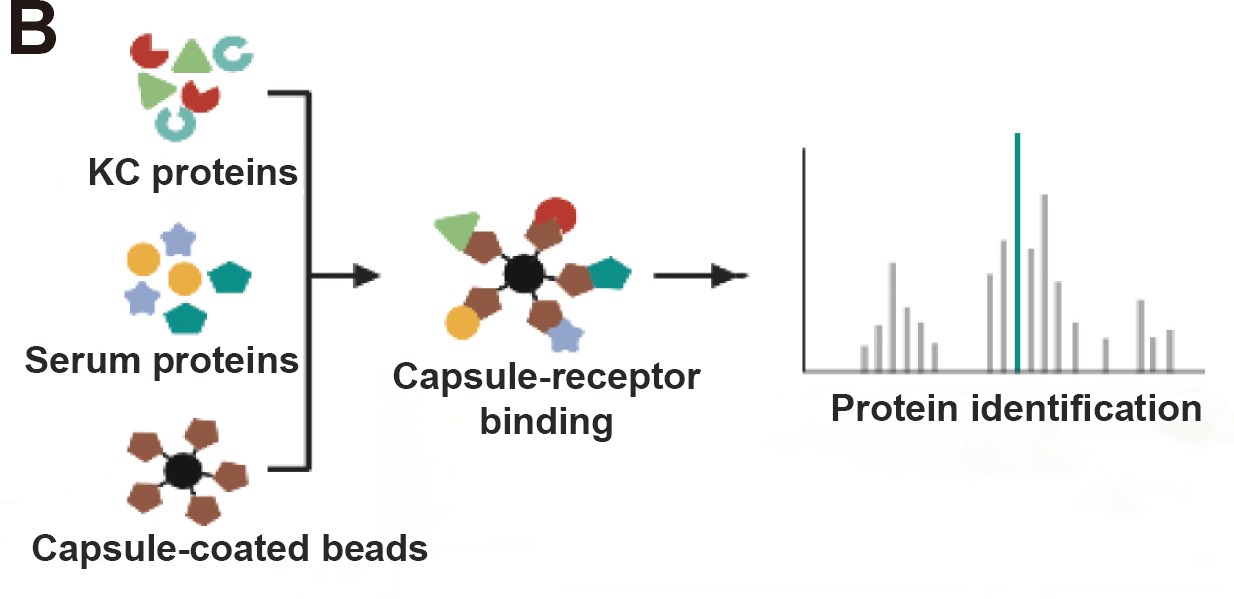

Supplement: Supplementary file 13 — Source data Fig. 1 [file 44318_2025_623_MOESM13_ESM.zip › SD figure 1/Figure 1B/Figure 1B.tif]

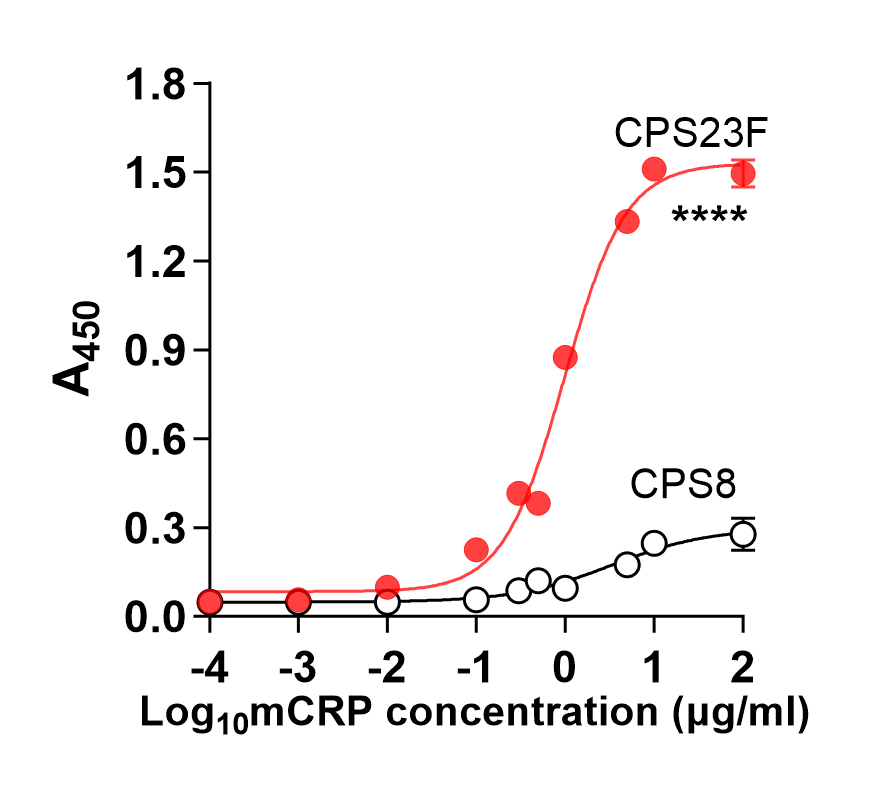

Supplement: Supplementary file 13 — Source data Fig. 1 [file 44318_2025_623_MOESM13_ESM.zip › SD figure 1/Figure 1D/Figure 1D.tif]

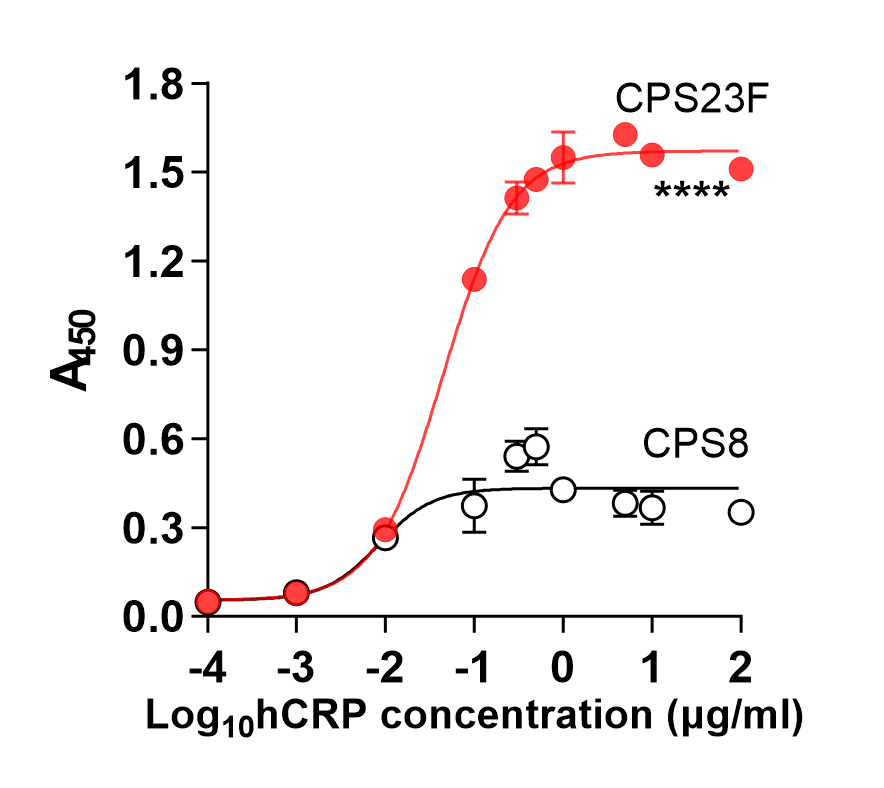

Supplement: Supplementary file 13 — Source data Fig. 1 [file 44318_2025_623_MOESM13_ESM.zip › SD figure 1/Figure 1J/Figure 1J.tif]

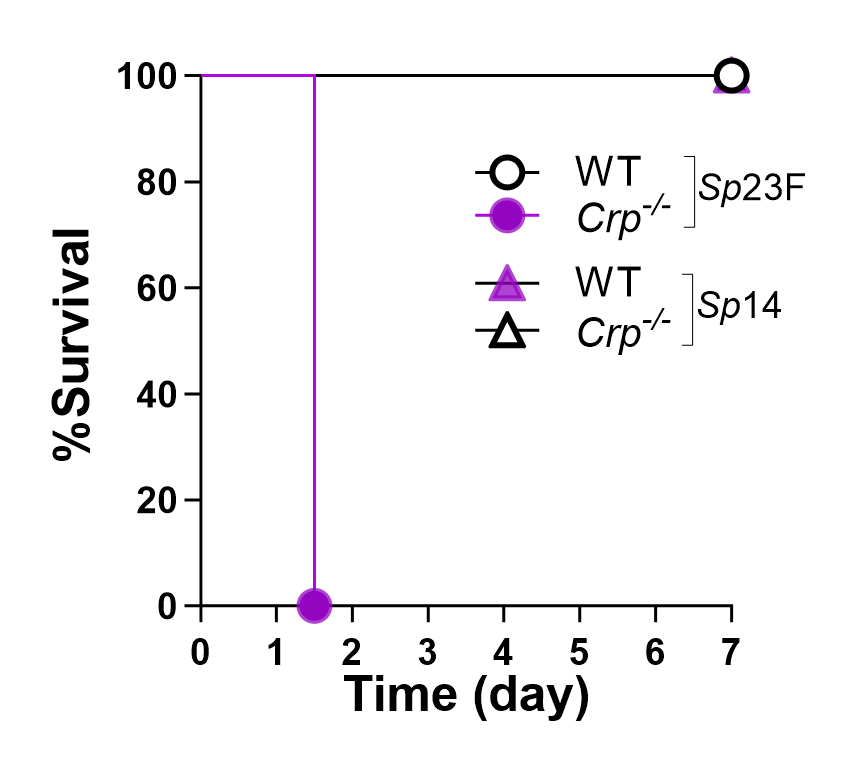

Supplement: Supplementary file 14 — Source data Fig. 2 [file 44318_2025_623_MOESM14_ESM.zip › SD figure 2/Figure 2G/Figure 2G.tif]

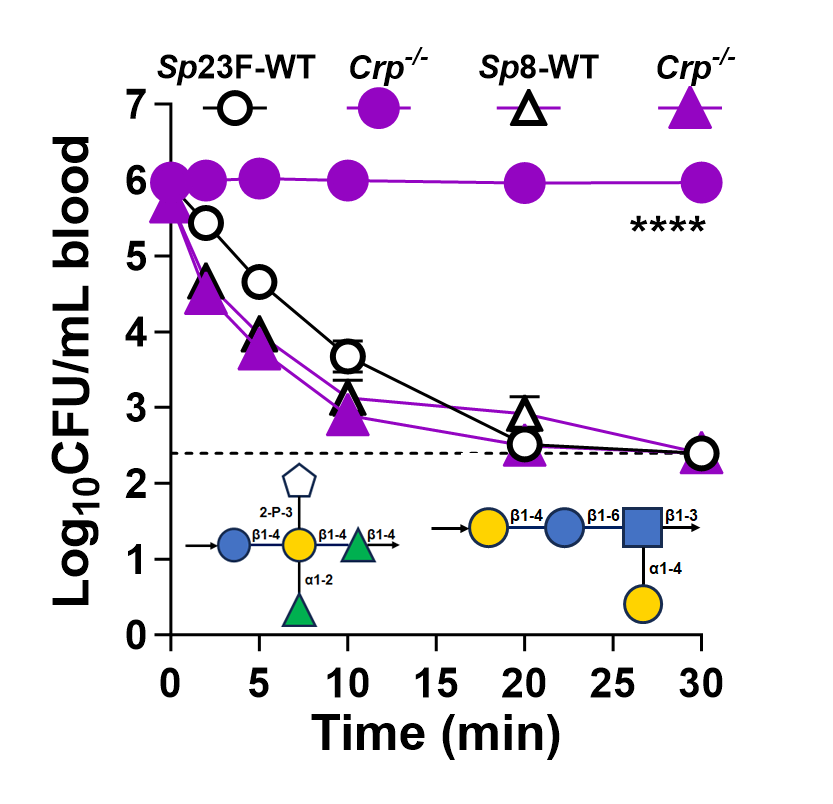

Supplement: Supplementary file 14 — Source data Fig. 2 [file 44318_2025_623_MOESM14_ESM.zip › SD figure 2/Figure 2A/Figure 2A.tif]

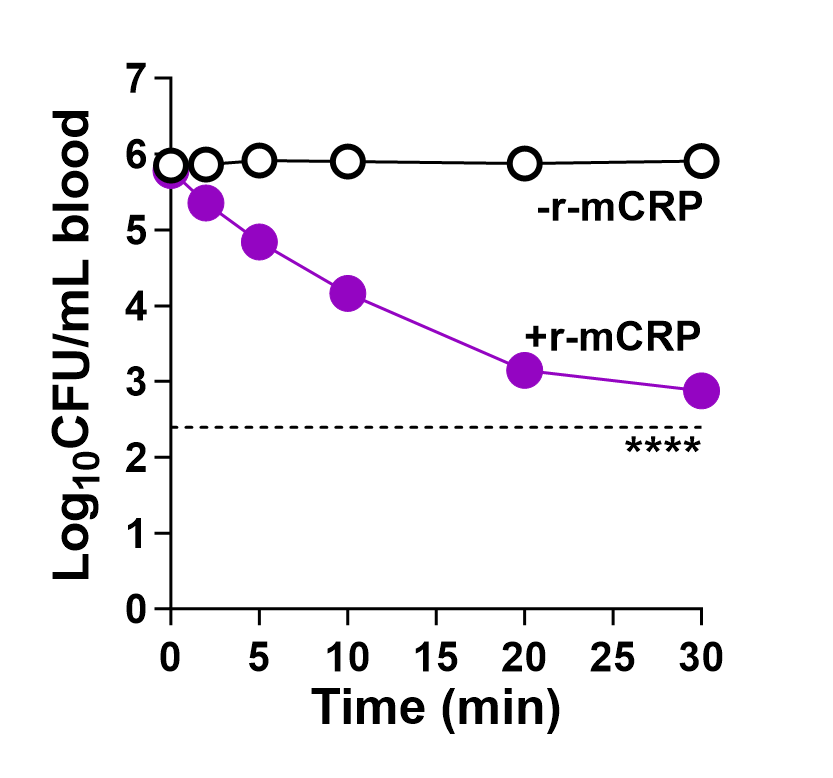

Supplement: Supplementary file 14 — Source data Fig. 2 [file 44318_2025_623_MOESM14_ESM.zip › SD figure 2/Figure 2F/Figure 2F left panel.tif]

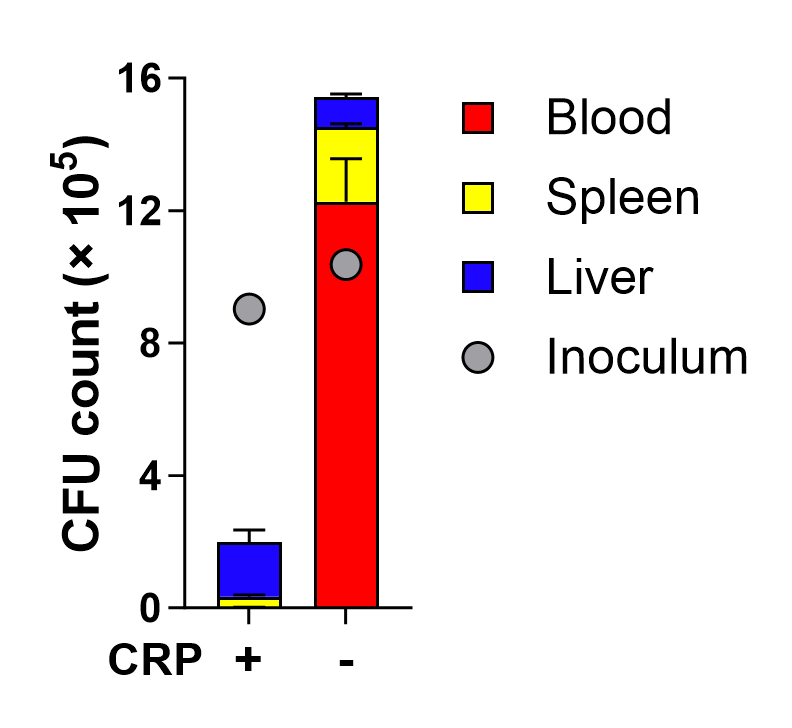

Supplement: Supplementary file 14 — Source data Fig. 2 [file 44318_2025_623_MOESM14_ESM.zip › SD figure 2/Figure 2F/Figure 2F right panel.tif]

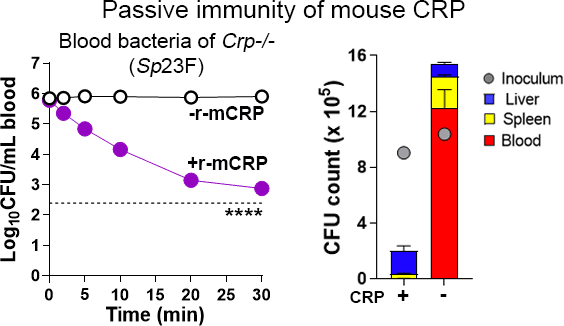

Supplement: Supplementary file 14 — Source data Fig. 2 [file 44318_2025_623_MOESM14_ESM.zip › SD figure 2/Figure 2F/Figure 2F.gif]

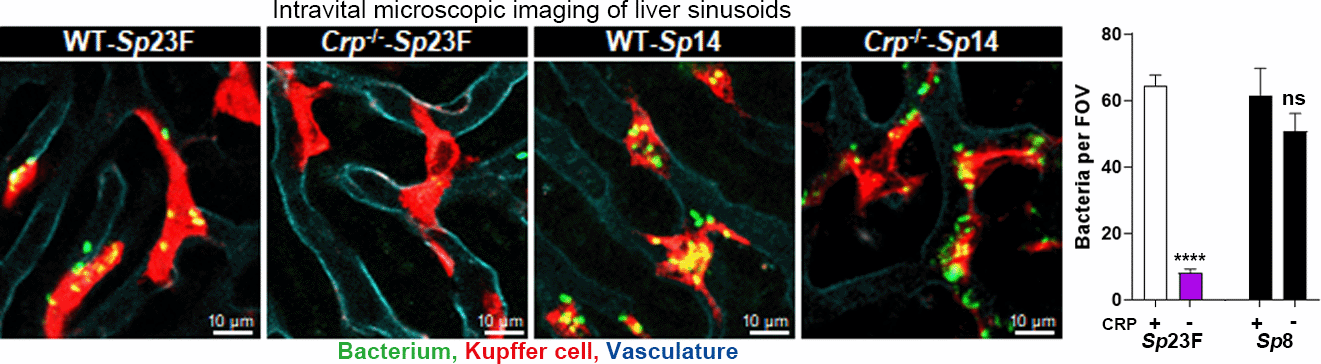

Supplement: Supplementary file 14 — Source data Fig. 2 [file 44318_2025_623_MOESM14_ESM.zip › SD figure 2/Figure 2D/Figure 2D.gif]

## Slide 1
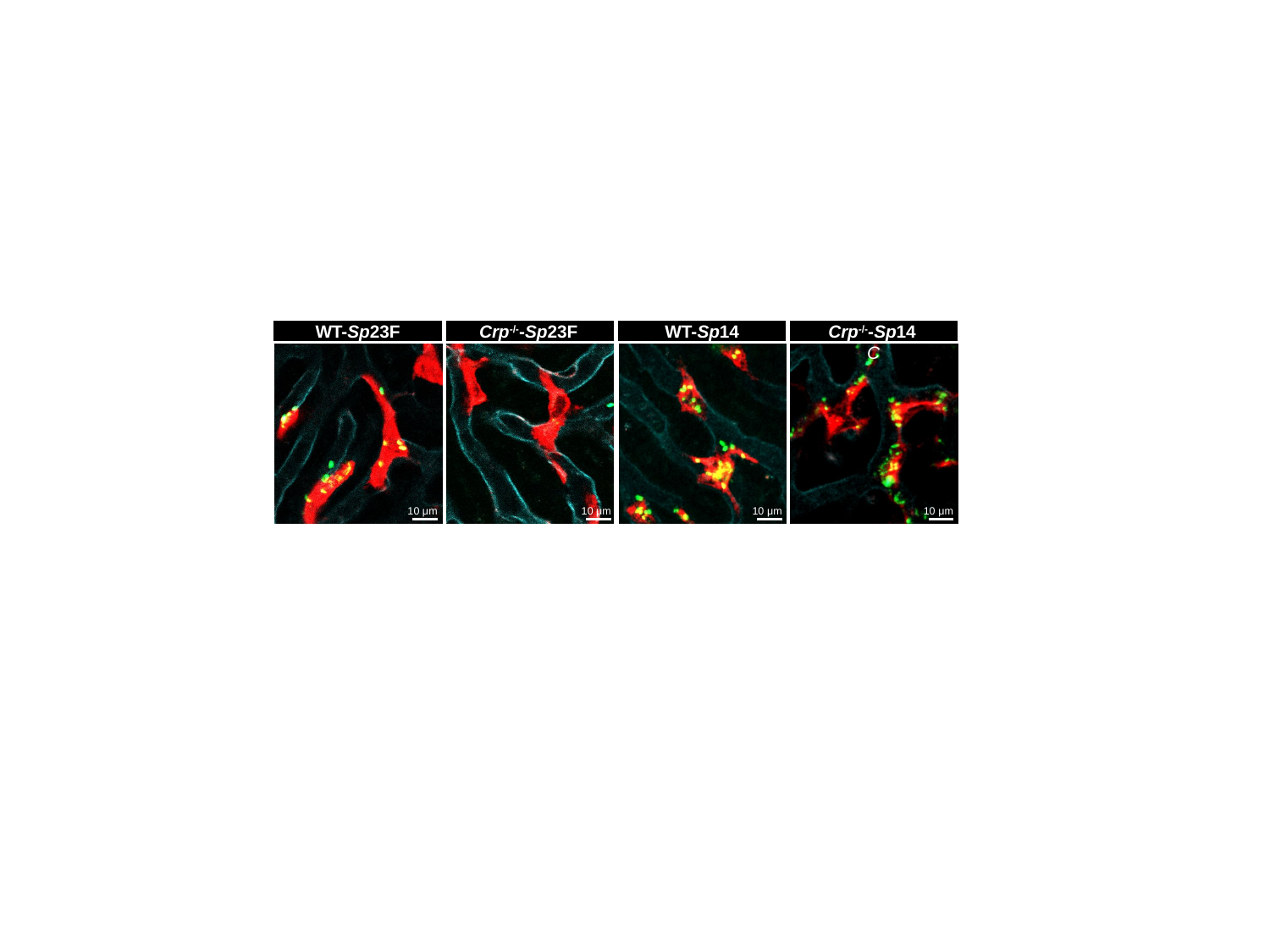

WT-Sp23F
Crp-/--Sp23F
C
WT-Sp14
Crp-/--Sp14
C
10 μm
10 μm
10 μm
10 μm

Supplement: Supplementary file 14 — Source data Fig. 2 [file 44318_2025_623_MOESM14_ESM.zip › SD figure 2/Figure 2D/Figure 2D left panel.pptx]

## Slide 1
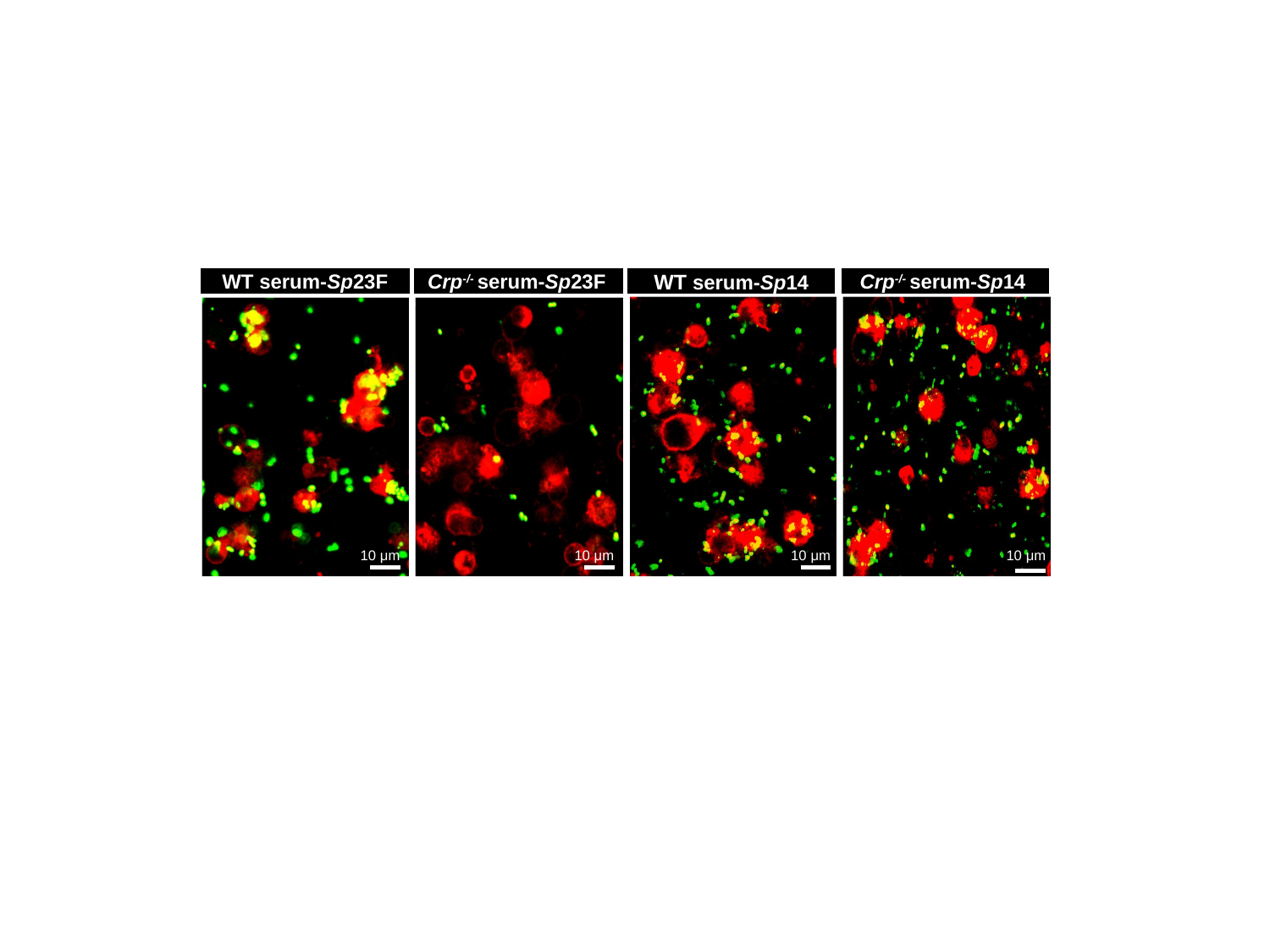

WT serum-Sp23F
WT serum-Sp14
Crp-/- serum-Sp14
Crp-/- serum-Sp23F
10 μm
10 μm
10 μm
10 μm

Supplement: Supplementary file 14 — Source data Fig. 2 [file 44318_2025_623_MOESM14_ESM.zip › SD figure 2/Figure 2E/Figure 2E.pptx]

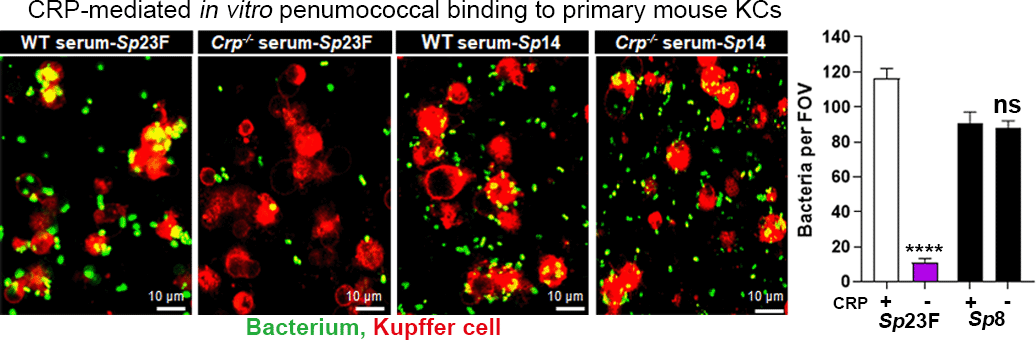

Supplement: Supplementary file 14 — Source data Fig. 2 [file 44318_2025_623_MOESM14_ESM.zip › SD figure 2/Figure 2E/Figure 2E.gif]

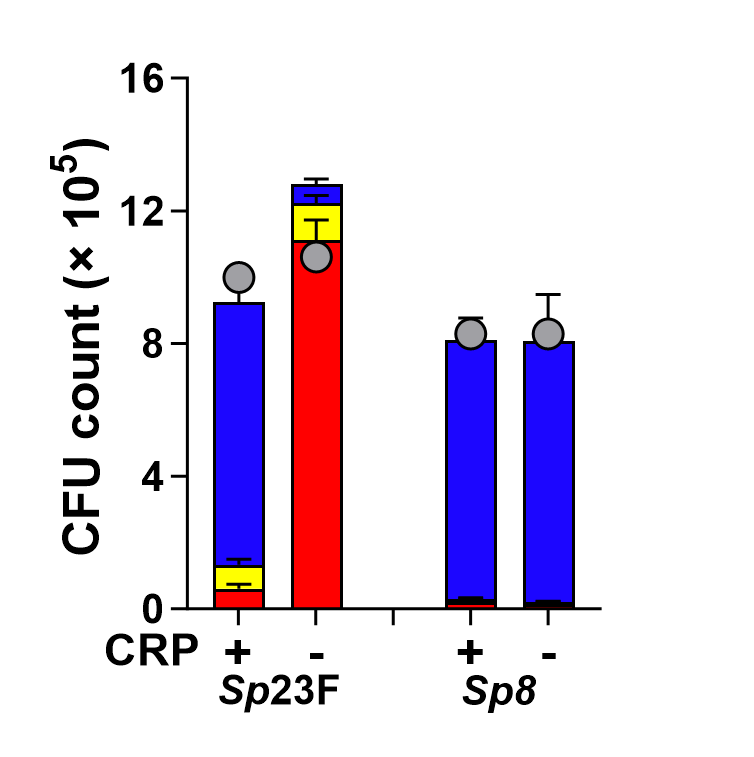

Supplement: Supplementary file 14 — Source data Fig. 2 [file 44318_2025_623_MOESM14_ESM.zip › SD figure 2/Figure 2B/Figure 2B left panel.tif]

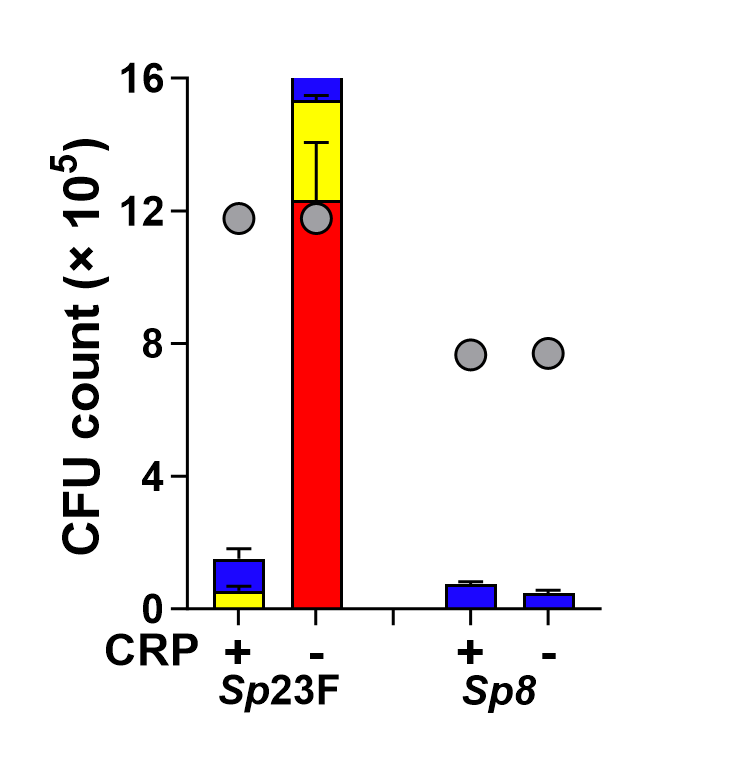

Supplement: Supplementary file 14 — Source data Fig. 2 [file 44318_2025_623_MOESM14_ESM.zip › SD figure 2/Figure 2B/Figure 2B right panel.tif]

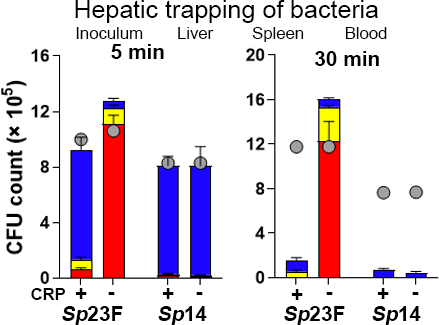

Supplement: Supplementary file 14 — Source data Fig. 2 [file 44318_2025_623_MOESM14_ESM.zip › SD figure 2/Figure 2B/Figure 2B.gif]

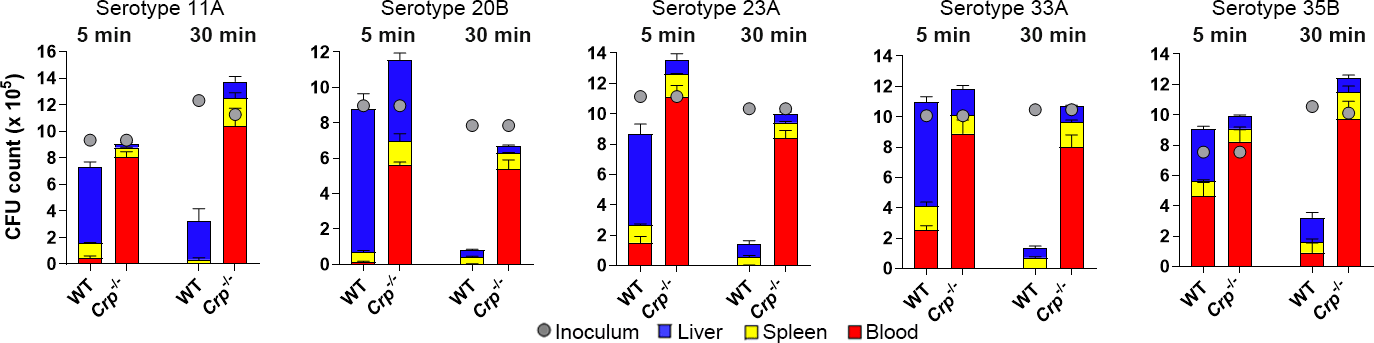

Supplement: Supplementary file 15 — Source data Fig. 3 [file 44318_2025_623_MOESM15_ESM.zip › SD figure 3/Figure 3B/Figure 3B.gif]

## Slide 1
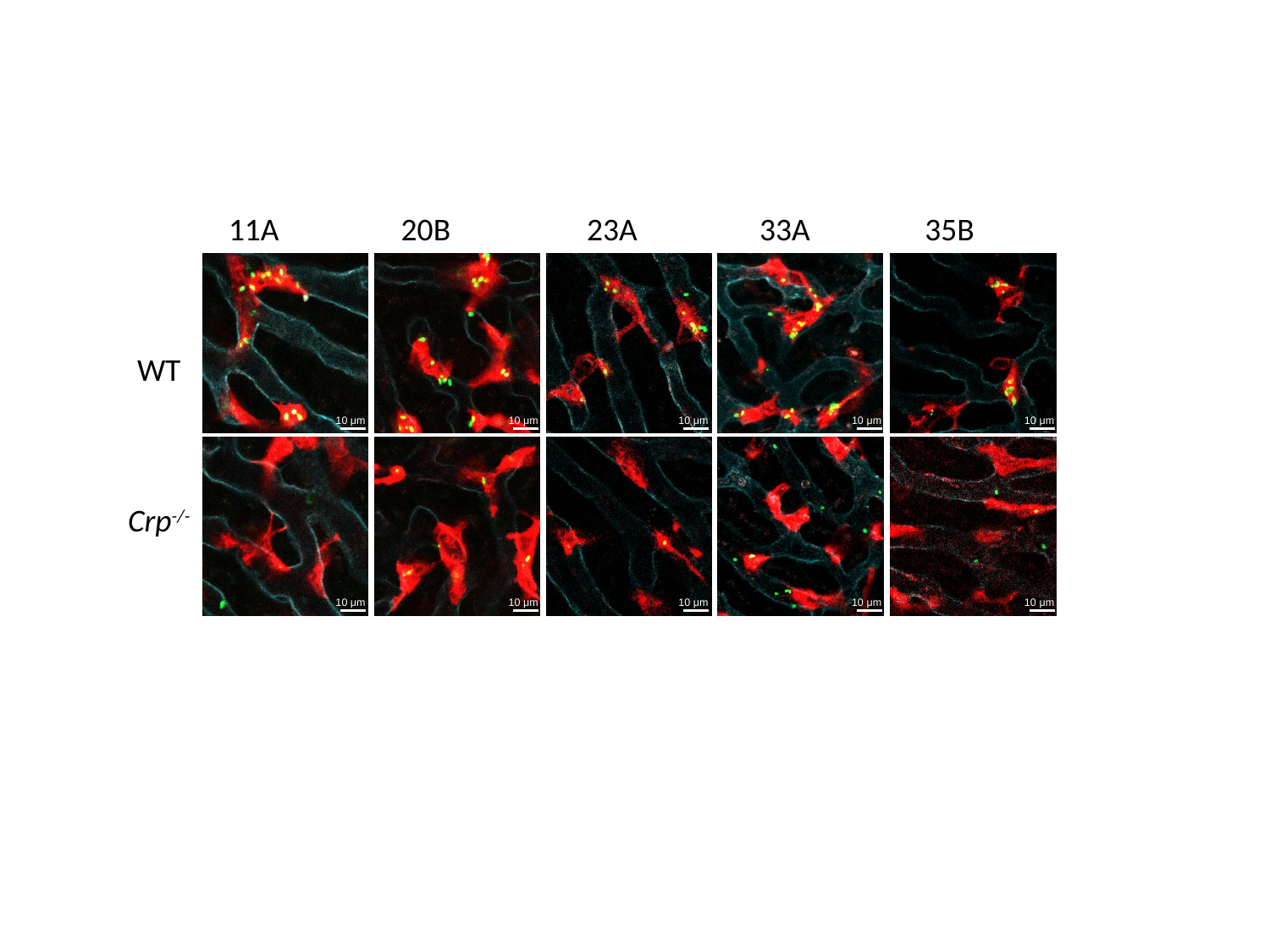

11A 20B 23A 33A 35B
10 μm
10 μm
10 μm
10 μm
10 μm
10 μm
10 μm
10 μm
10 μm
10 μm
WT
Crp-/-

Supplement: Supplementary file 15 — Source data Fig. 3 [file 44318_2025_623_MOESM15_ESM.zip › SD figure 3/Figure 3C/Figure 3.pptx]

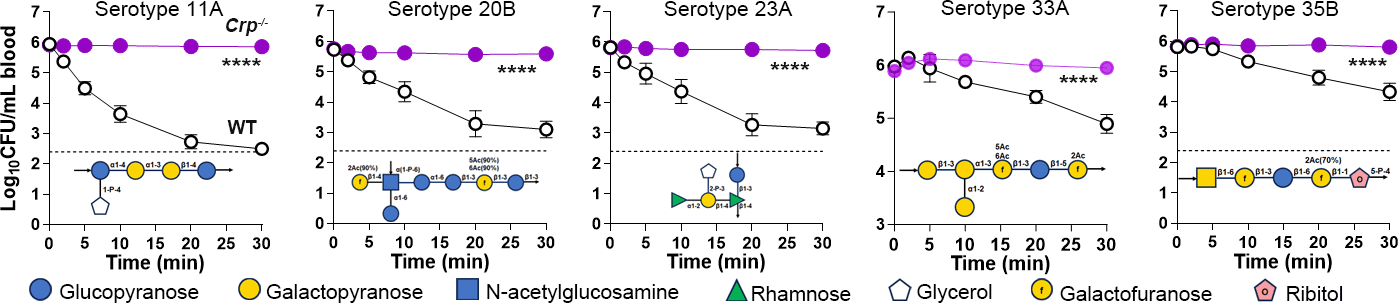

Supplement: Supplementary file 15 — Source data Fig. 3 [file 44318_2025_623_MOESM15_ESM.zip › SD figure 3/Figure 3A/Figure 3A.gif]

## Slide 1
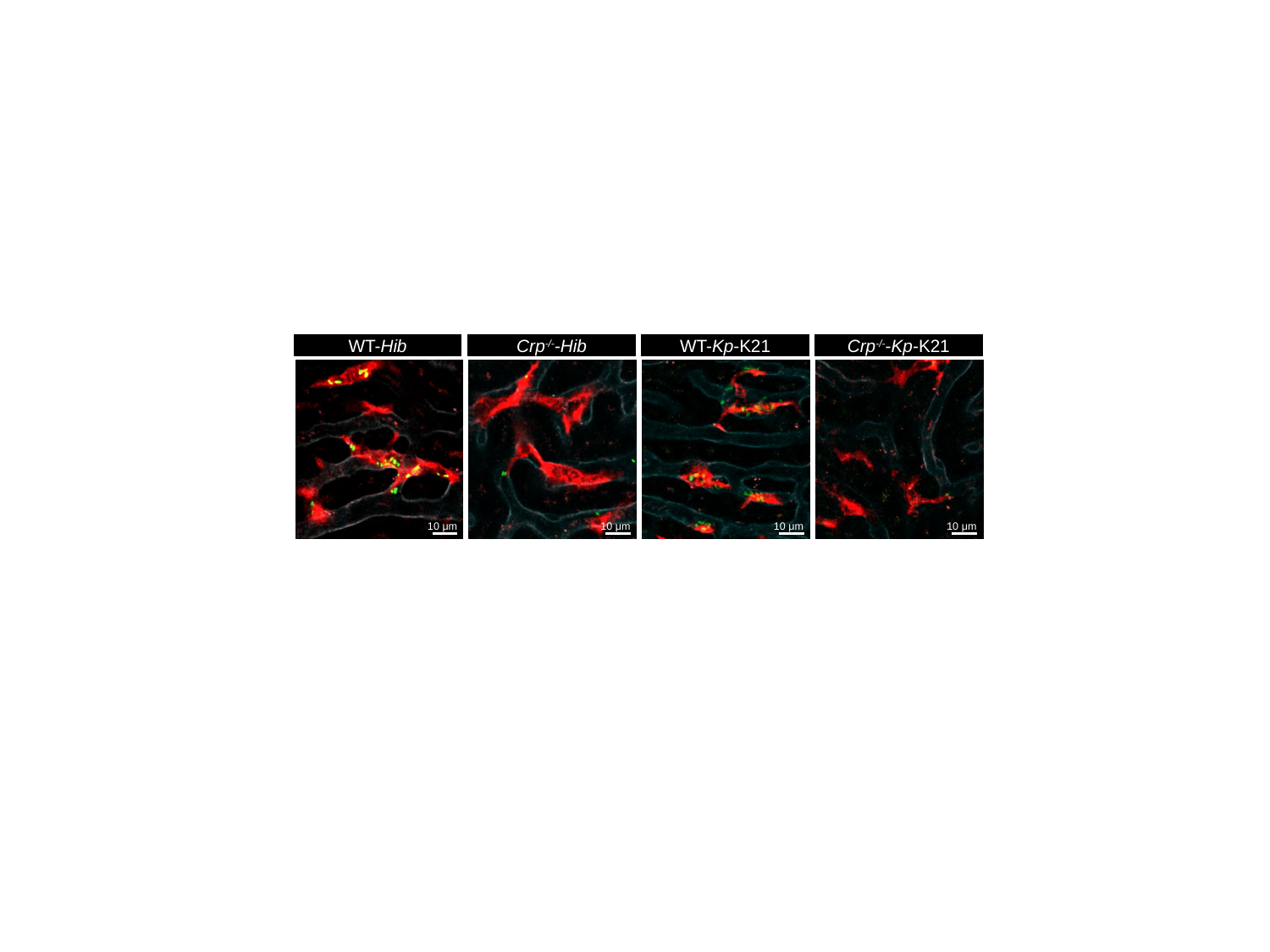

WT-Hib
Crp-/--Hib
WT-Kp-K21
Crp-/--Kp-K21
10 μm
10 μm
10 μm
10 μm

Supplement: Supplementary file 16 — Source data Fig. 4 [file 44318_2025_623_MOESM16_ESM.zip › SD figure 4/Figure 4E/Figure 4E.pptx]

## Slide 1
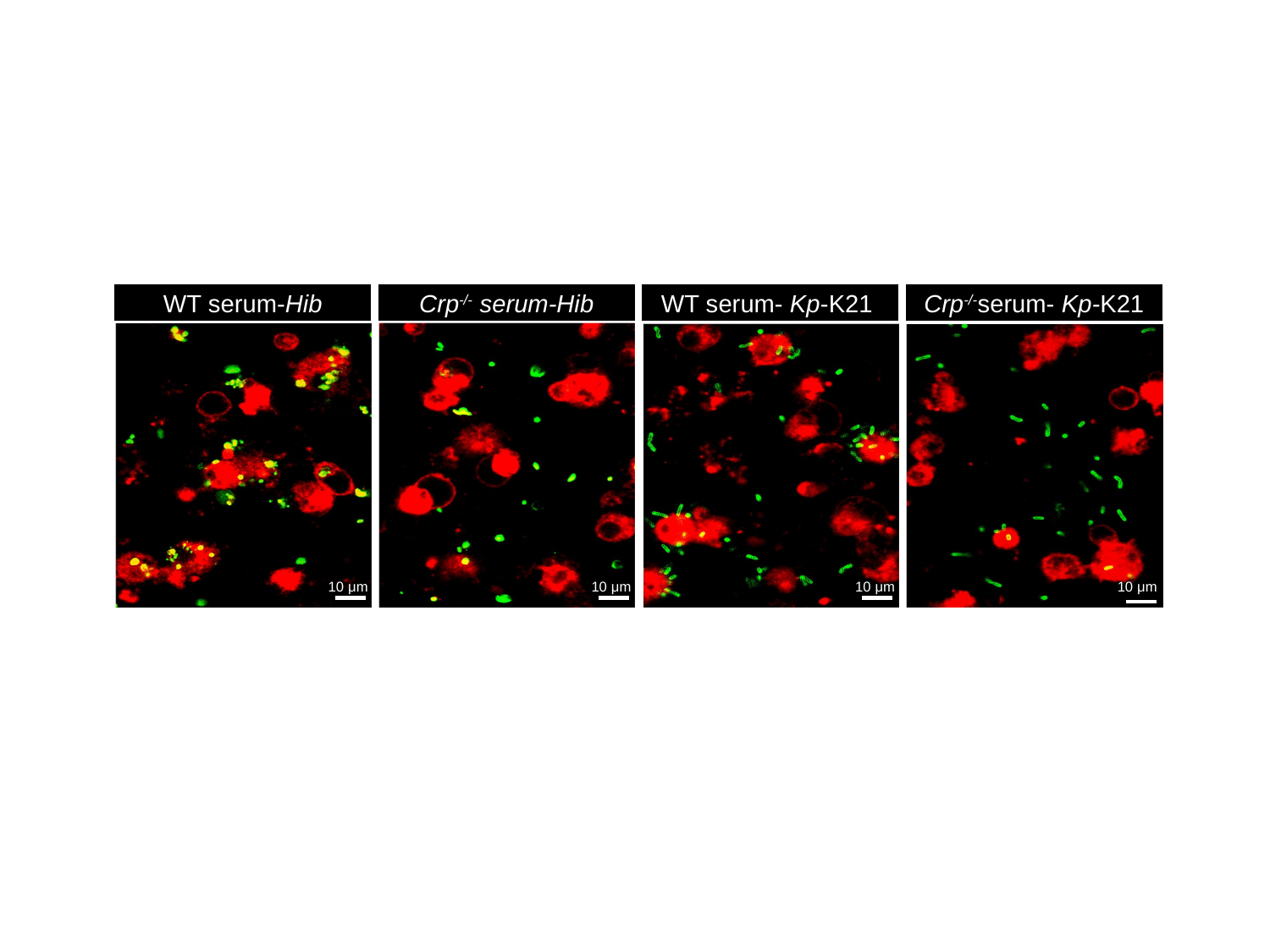

WT serum-Hib
Crp-/- serum-Hib
WT serum- Kp-K21
Crp-/-serum- Kp-K21
10 μm
10 μm
10 μm
10 μm

Supplement: Supplementary file 16 — Source data Fig. 4 [file 44318_2025_623_MOESM16_ESM.zip › SD figure 4/Figure 4F/Figure 4F.pptx]

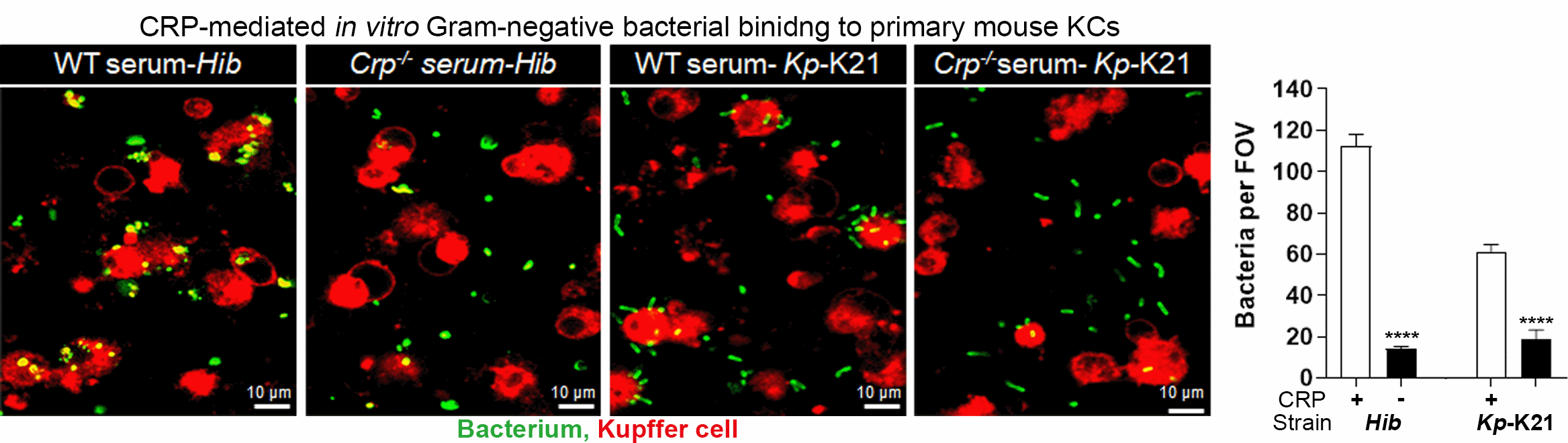

Supplement: Supplementary file 16 — Source data Fig. 4 [file 44318_2025_623_MOESM16_ESM.zip › SD figure 4/Figure 4F/Figure 4F.gif]

## Slide 1
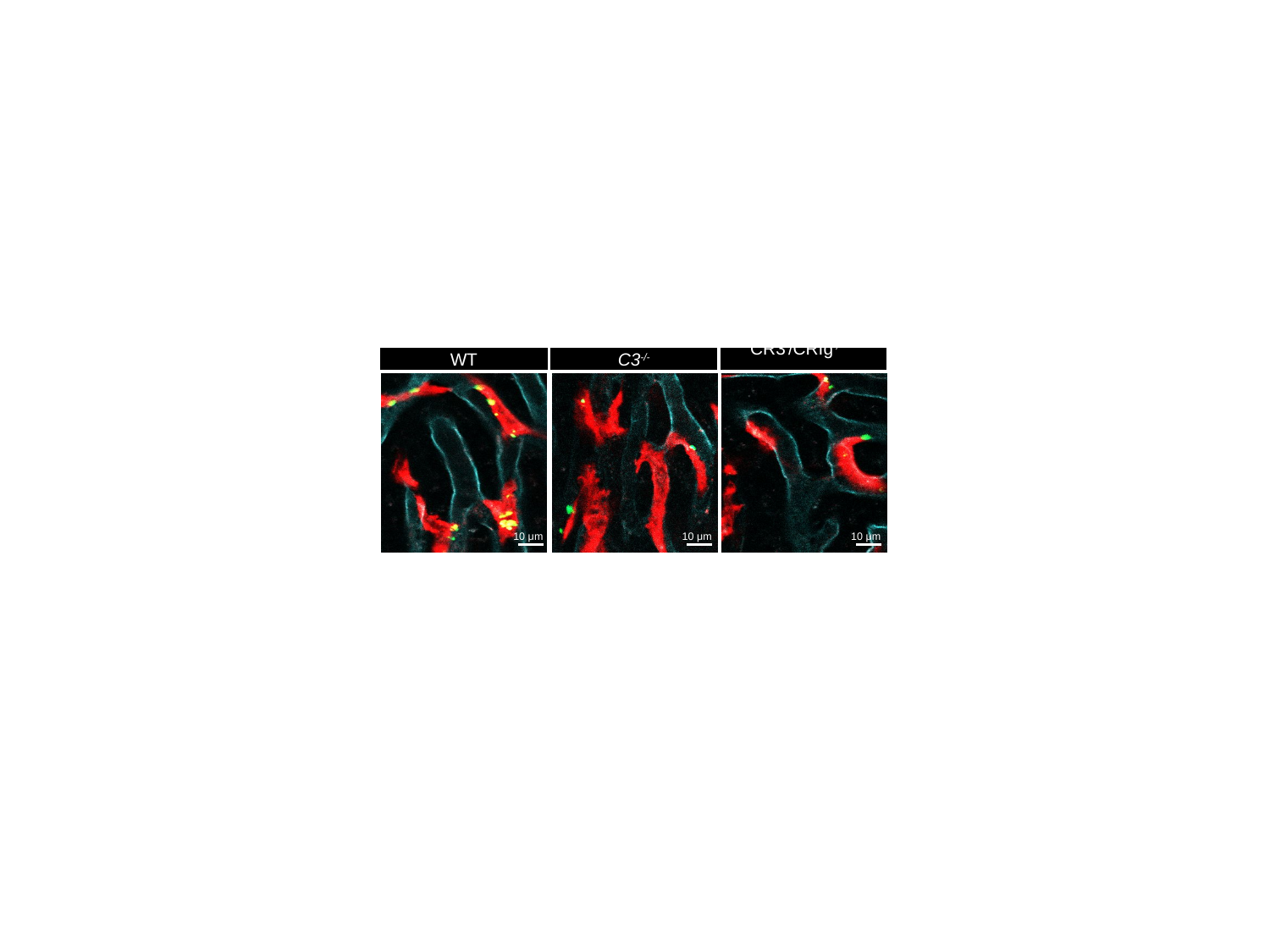

WT
C3-/-
CR3 /CRIg-/-
10 μm
10 μm
10 μm

Supplement: Supplementary file 17 — Source data Fig. 5 [file 44318_2025_623_MOESM17_ESM.zip › SD figure 5/Figure 5E/Figure 5E.pptx]

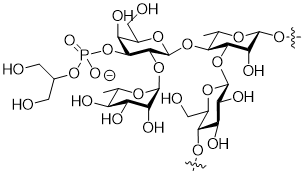

Supplement: Supplementary file 18 — Source data Fig. 6 [file 44318_2025_623_MOESM18_ESM.zip › SD figure 6/Figure 6A/CPS23F.tif]

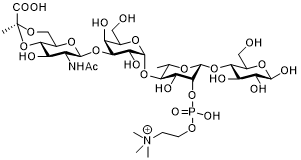

Supplement: Supplementary file 18 — Source data Fig. 6 [file 44318_2025_623_MOESM18_ESM.zip › SD figure 6/Figure 6A/CPS27.tif]

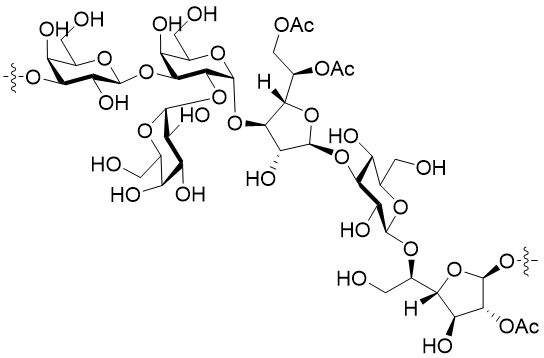

Supplement: Supplementary file 18 — Source data Fig. 6 [file 44318_2025_623_MOESM18_ESM.zip › SD figure 6/Figure 6A/CPS33A.tif]

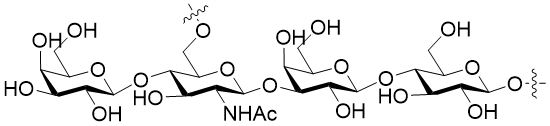

Supplement: Supplementary file 18 — Source data Fig. 6 [file 44318_2025_623_MOESM18_ESM.zip › SD figure 6/Figure 6A/CPS14.tif]

Time (min)

0 10 20 30 40 50 60 70 80 90 100

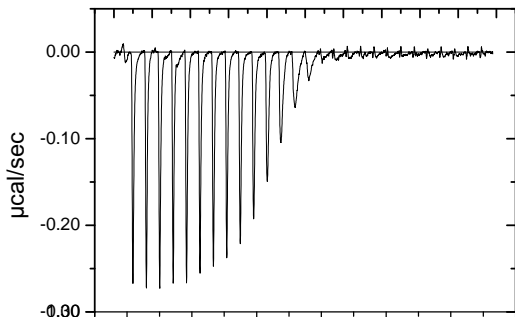

kcal mol<sup>-1</sup> of injectant

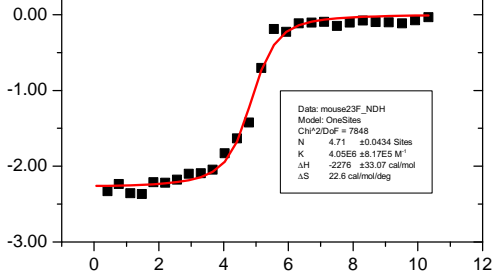

Molar Ratio

Supplement: Supplementary file 18 — Source data Fig. 6 [file 44318_2025_623_MOESM18_ESM.zip › SD figure 6/Figure 6B/CRP-CPS23F.PDF]

Time (min)

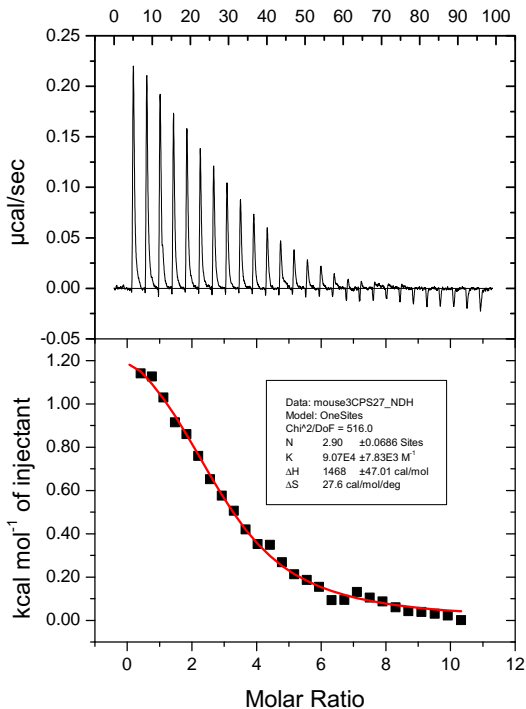

Supplement: Supplementary file 18 — Source data Fig. 6 [file 44318_2025_623_MOESM18_ESM.zip › SD figure 6/Figure 6B/CRP-CPS27.PDF]

Time (min)

0 10 20 30 40 50 60 70 80 90 100

0.08

0.04

0.00

-0.04

$\mu\text{cal/sec}$

0.80

0.40

0.00

-0.40

-0.80

$\text{kcal mol}^{-1}$  of injectant

0

2

4

6

8

10

12

Molar Ratio

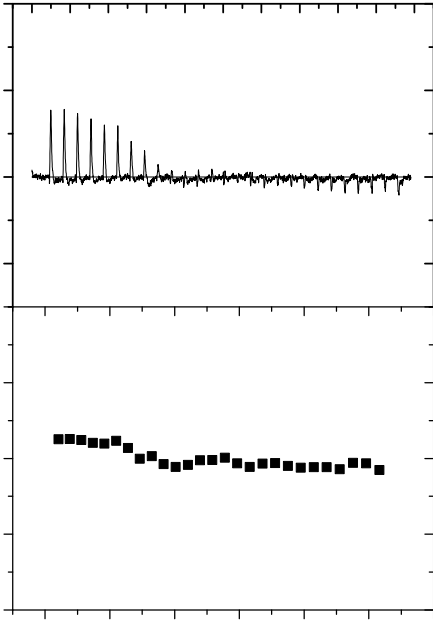

Supplement: Supplementary file 18 — Source data Fig. 6 [file 44318_2025_623_MOESM18_ESM.zip › SD figure 6/Figure 6B/CRP-CPS14.PDF]

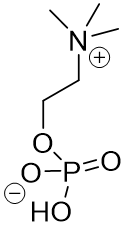

Supplement: Supplementary file 18 — Source data Fig. 6 [file 44318_2025_623_MOESM18_ESM.zip › SD figure 6/Figure 6D/PG.tif]

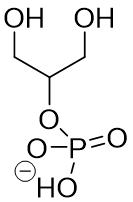

Supplement: Supplementary file 18 — Source data Fig. 6 [file 44318_2025_623_MOESM18_ESM.zip › SD figure 6/Figure 6C/PC.tif]

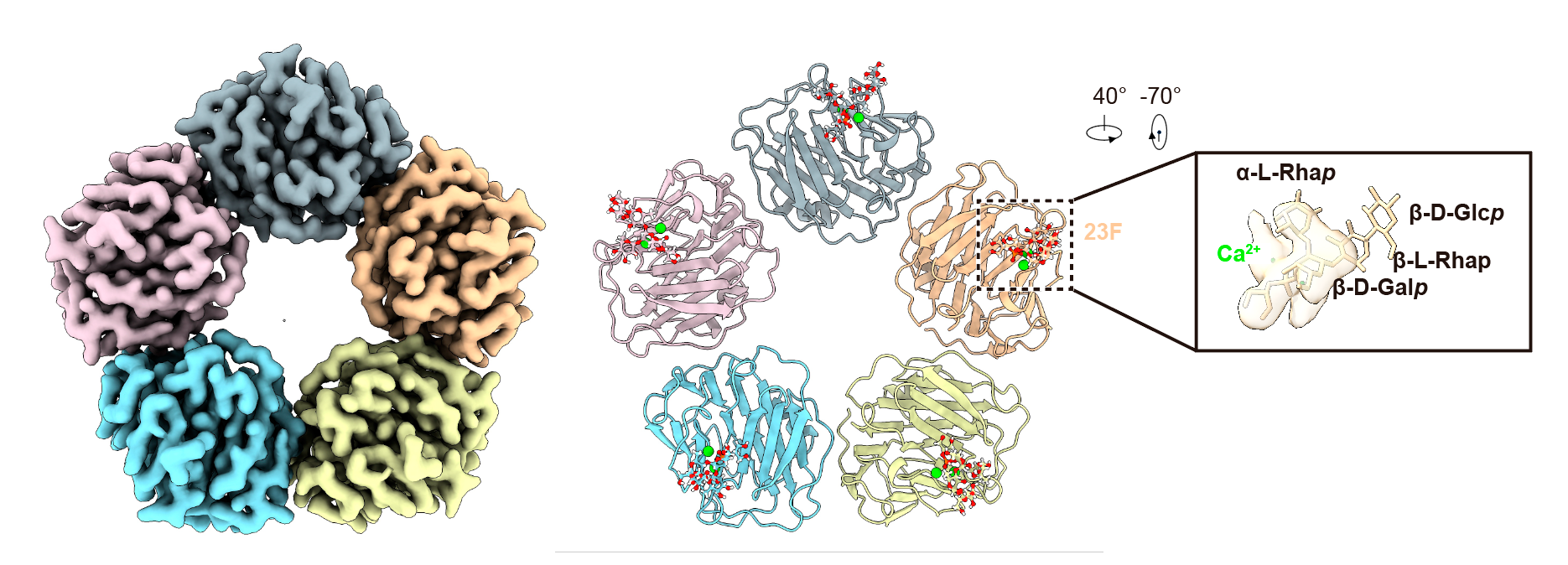

Supplement: Supplementary file 19 — Source data Fig. 7 [file 44318_2025_623_MOESM19_ESM.zip › SD figure 7/Figure 7D/Figure 7D.png]

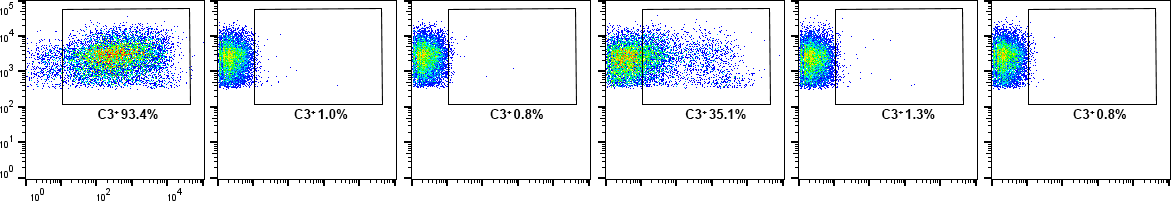

Supplement: Supplementary file 19 — Source data Fig. 7 [file 44318_2025_623_MOESM19_ESM.zip › SD figure 7/Figure 7C/Figure 7C.tiff]

23F

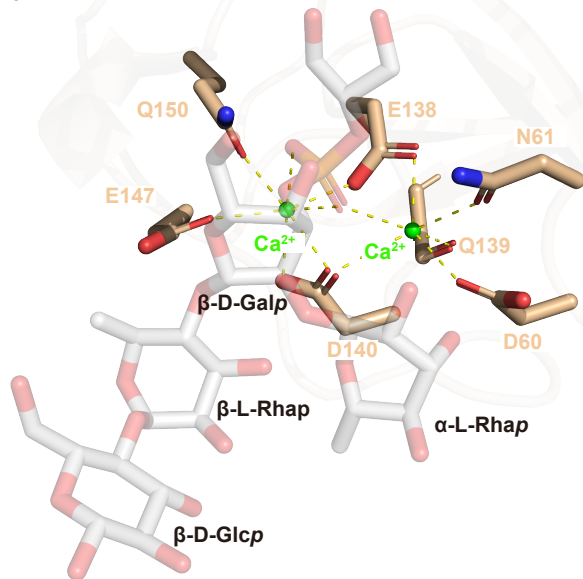

phosphocholine

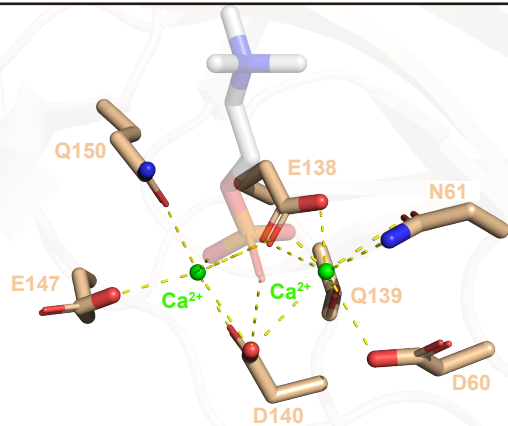

Supplement: Supplementary file 19 — Source data Fig. 7 [file 44318_2025_623_MOESM19_ESM.zip › SD figure 7/Figure 7E/Figure 7E.pdf]

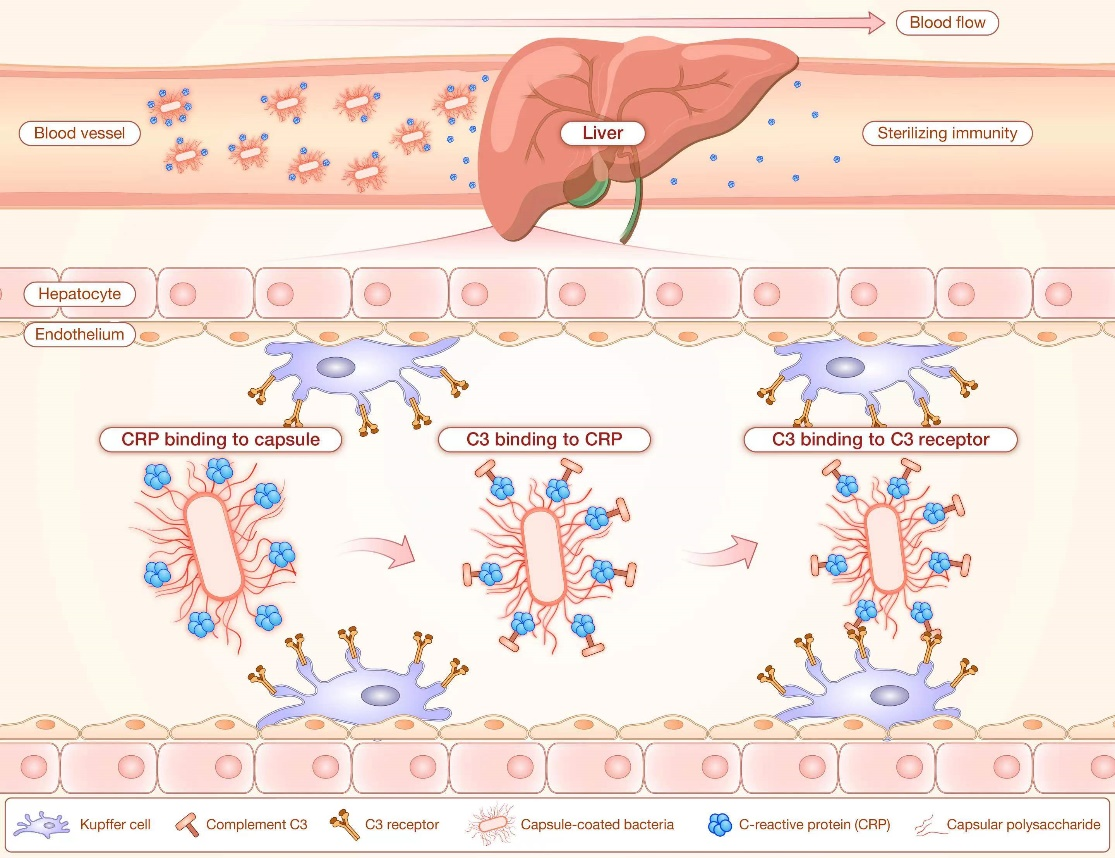

Supplement: Supplementary file 20 — Source data Fig. 8 [file 44318_2025_623_MOESM20_ESM.zip › SD figure 8/Figure 8.tif]

**A**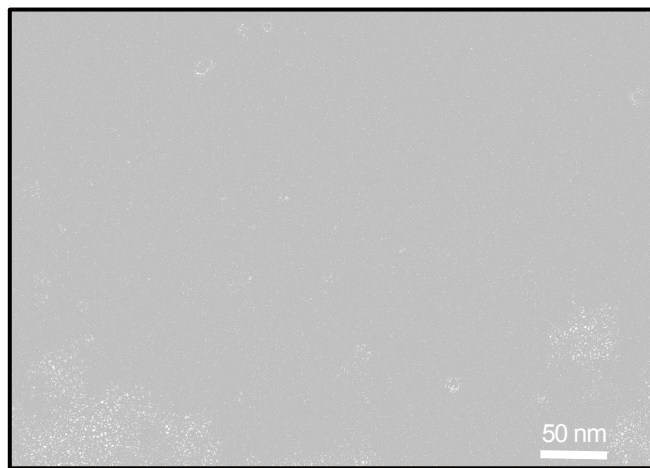**B**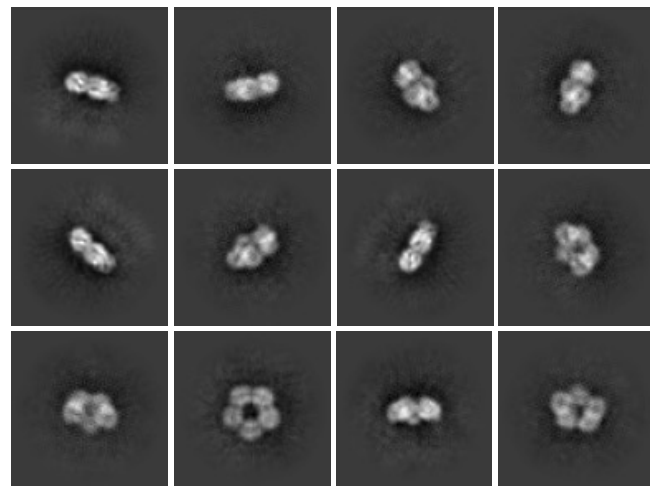**D**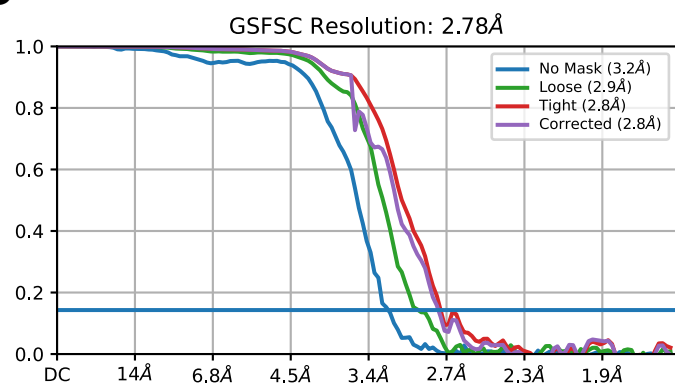**E**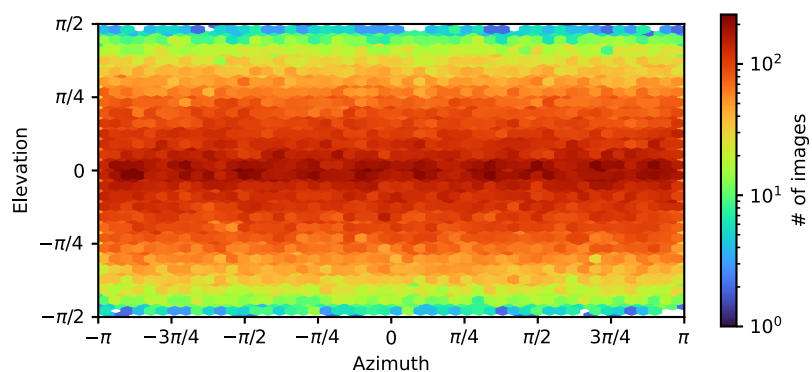**F**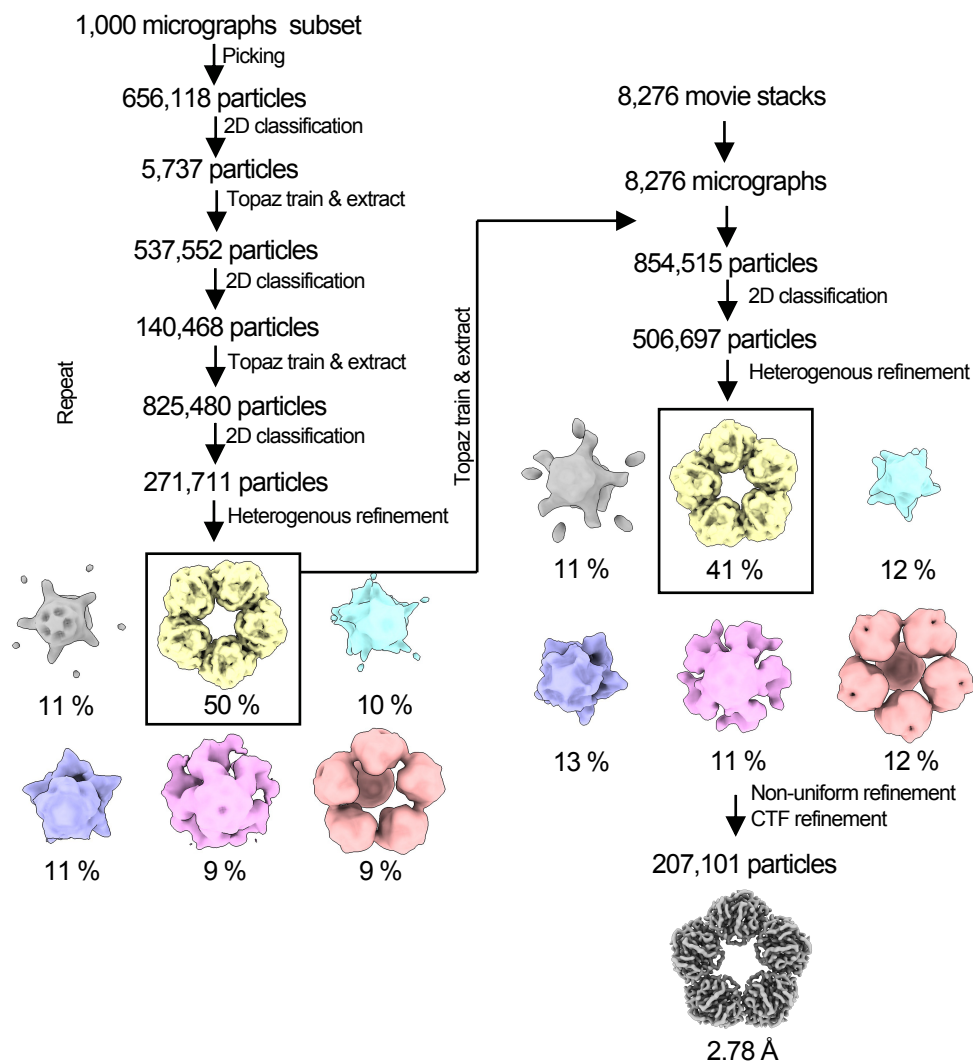

Supplement: Supplementary file 21 — Figure EV and appendix source Data [file 44318_2025_623_MOESM21_ESM.zip › Source data/Appendix Figure S9/Figure S9.pdf]

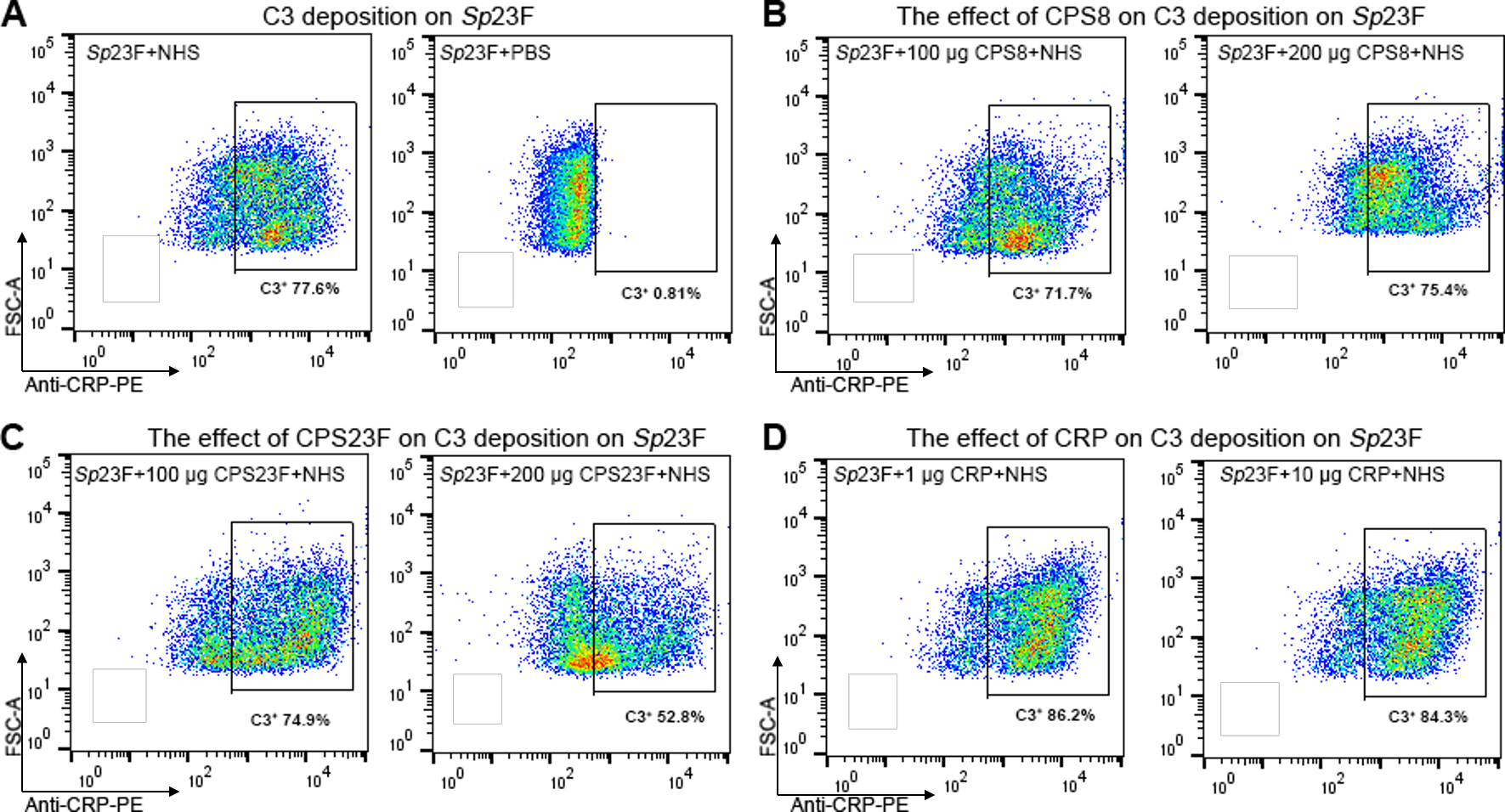

Supplement: Supplementary file 21 — Figure EV and appendix source Data [file 44318_2025_623_MOESM21_ESM.zip › Source data/Appendix Figure S8/Figure S8.tif]

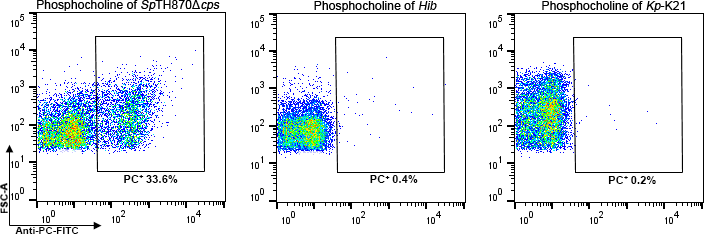

Supplement: Supplementary file 21 — Figure EV and appendix source Data [file 44318_2025_623_MOESM21_ESM.zip › Source data/Appendix Figure S1/Figure S1.tif]

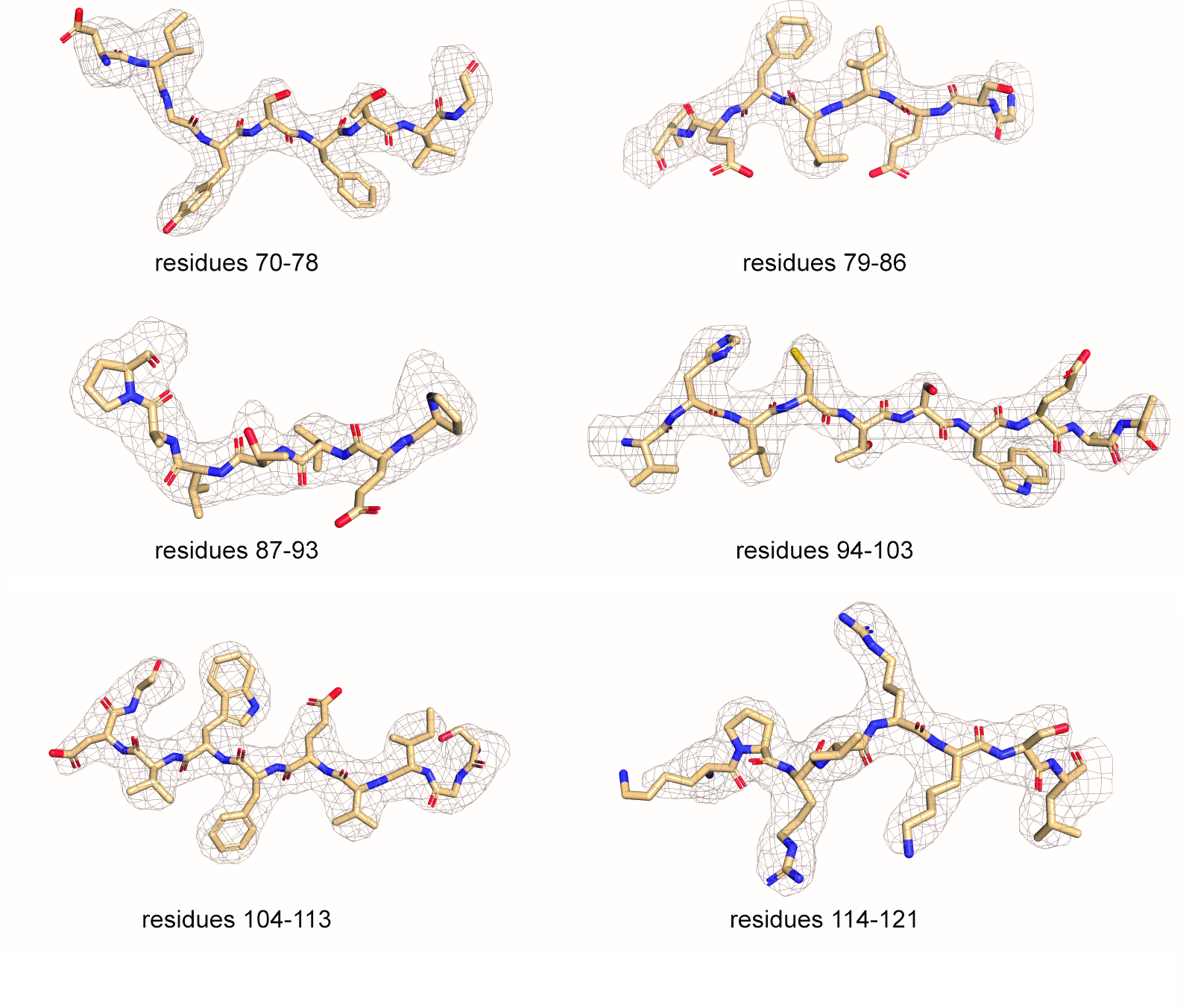

Supplement: Supplementary file 21 — Figure EV and appendix source Data [file 44318_2025_623_MOESM21_ESM.zip › Source data/Appendix Figure S10/Figure S10.tif]

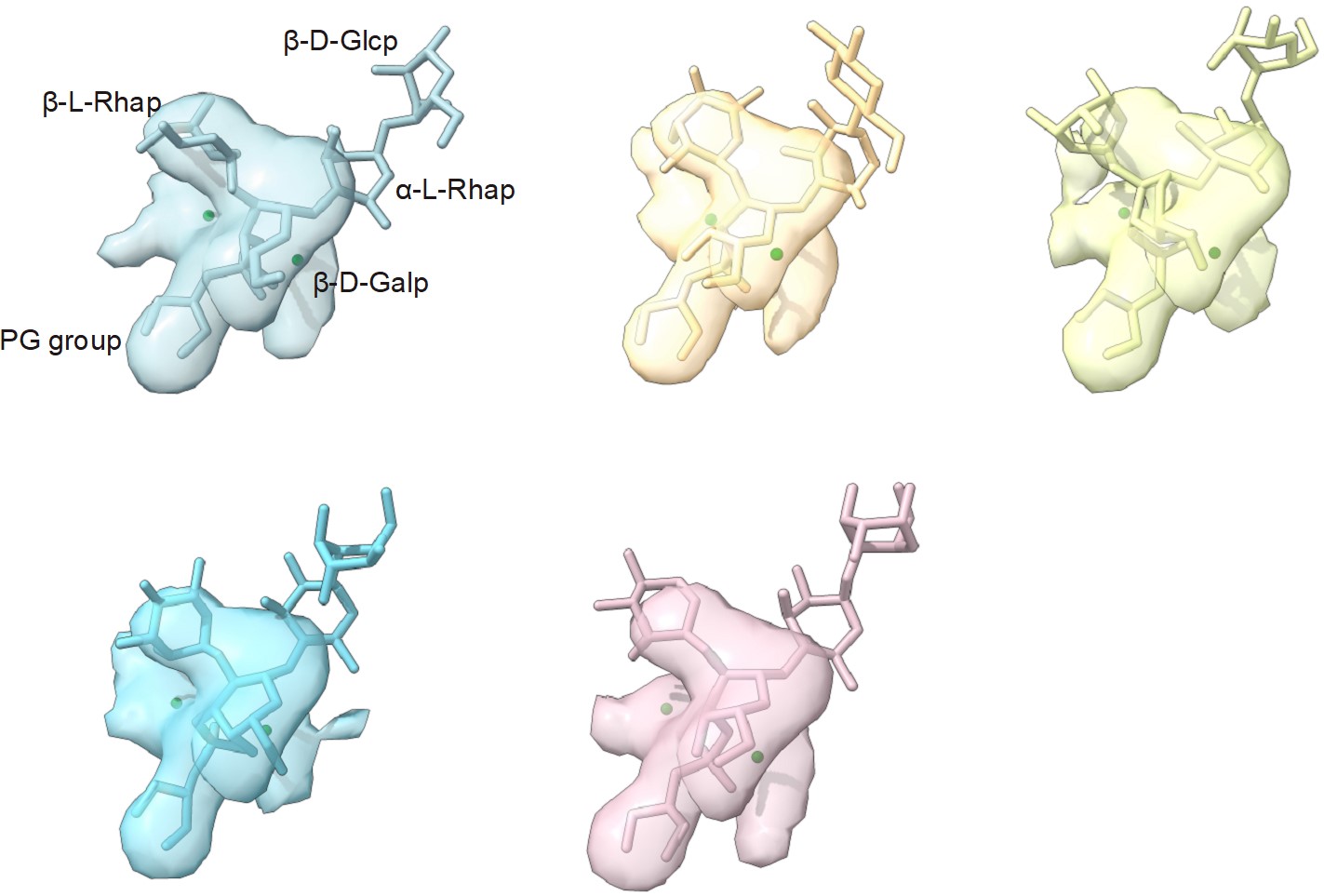

Supplement: Supplementary file 21 — Figure EV and appendix source Data [file 44318_2025_623_MOESM21_ESM.zip › Source data/Figure EV5/Figure EV5.tif.jpg]

## Slide 1
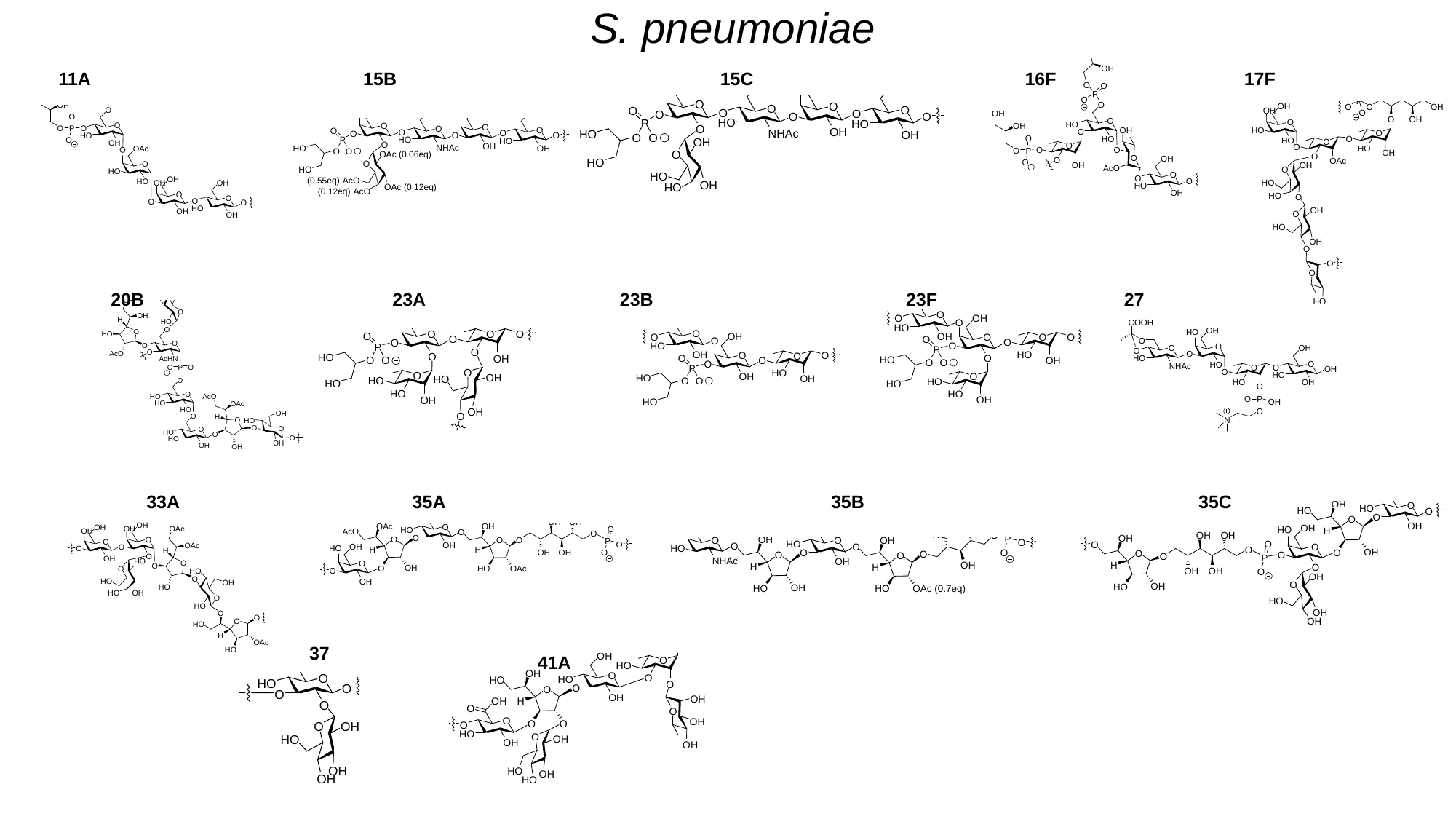

S. pneumoniae
11A
15B
15C
16F
17F
20B
23A
23B
23F
27
33A
35A
35B
35C
37
41A

## Slide 2
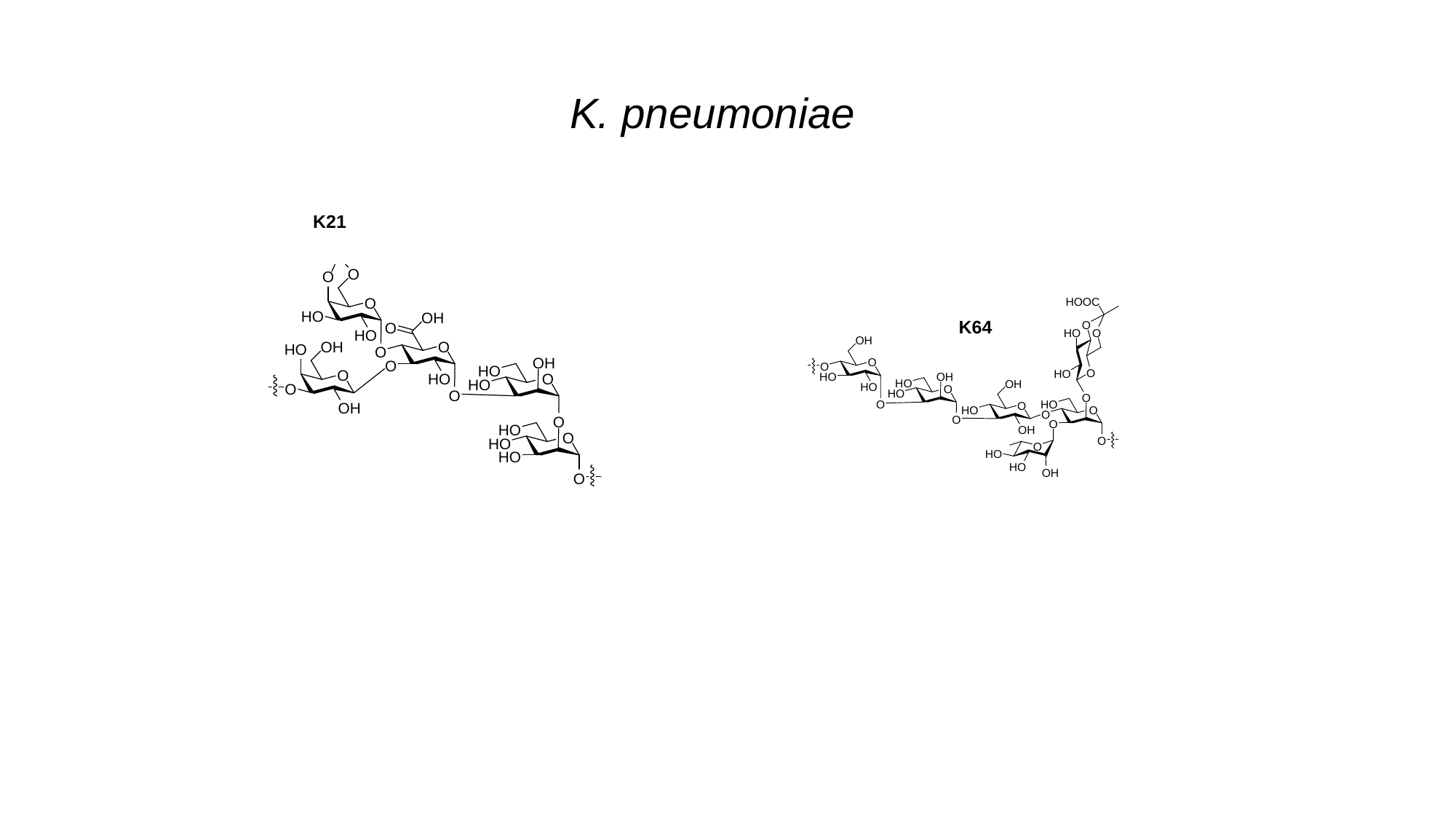

K. pneumoniae
K21
K64

## Slide 3
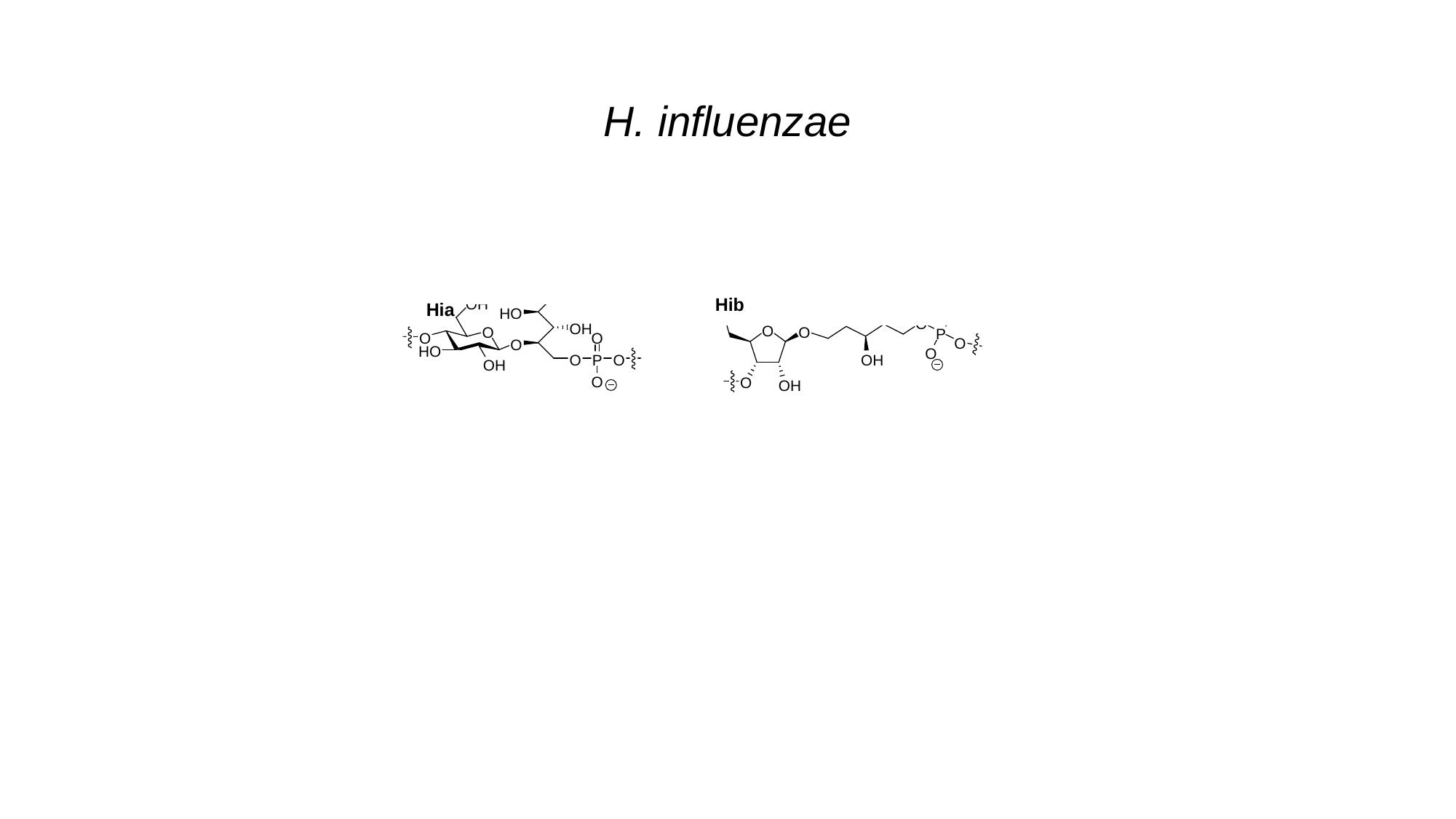

# H. influenzae
Hib
Hia

Supplement: Supplementary file 21 — Figure EV and appendix source Data [file 44318_2025_623_MOESM21_ESM.zip › Source data/Appendix Figure S5/Figure S5.pptx]

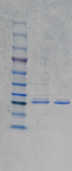

Supplement: Supplementary file 21 — Figure EV and appendix source Data [file 44318_2025_623_MOESM21_ESM.zip › Source data/Figure EV1/Figure EV1A.tif]

Time (min)

0 10 20 30 40 50 60 70 80 90 100

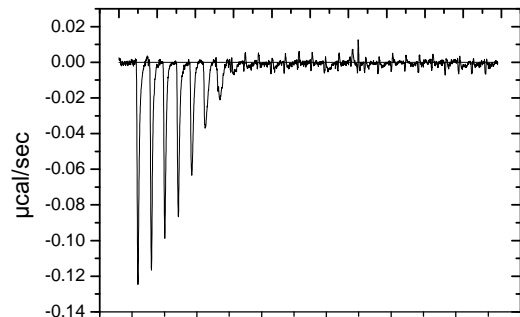

$\text{kcal mol}^{-1}$  of injectant

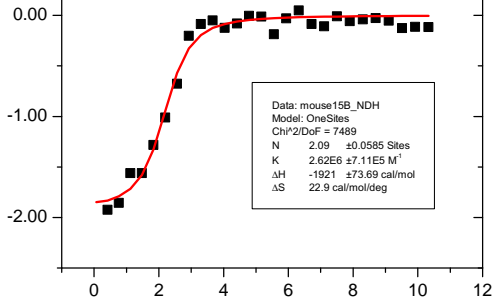

Molar Ratio

Supplement: Supplementary file 21 — Figure EV and appendix source Data [file 44318_2025_623_MOESM21_ESM.zip › Source data/Appendix Figure S6/Figure S6B/CPS15B-mCRP.PDF]

Time (min)

0 10 20 30 40 50 60 70 80 90 100

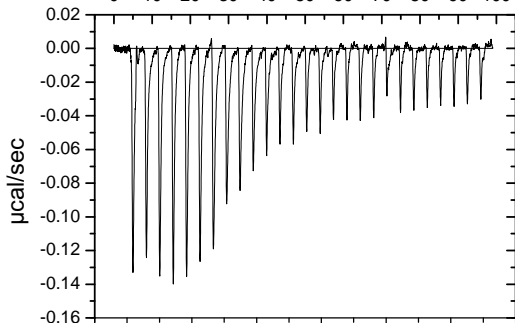

$\text{kcal mol}^{-1}$  of injectant

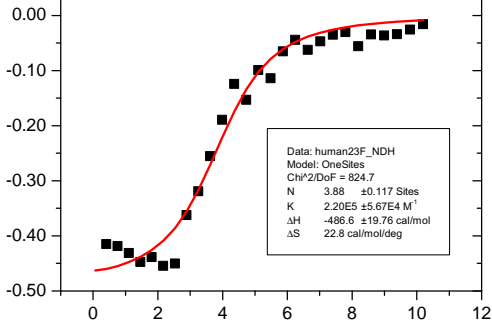

Molar Ratio

Supplement: Supplementary file 21 — Figure EV and appendix source Data [file 44318_2025_623_MOESM21_ESM.zip › Source data/Appendix Figure S6/Figure S6E/CPS23F-hCRP.PDF]

Time (min)

0 10 20 30 40 50 60 70 80 90 100

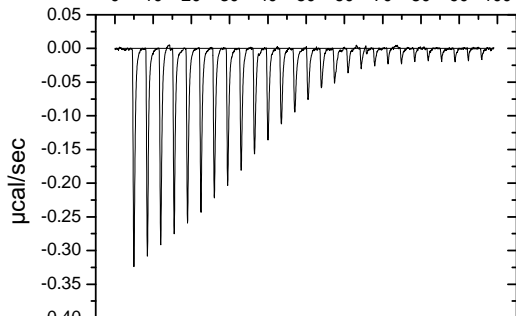

$\text{kcal mol}^{-1}$  of injectant

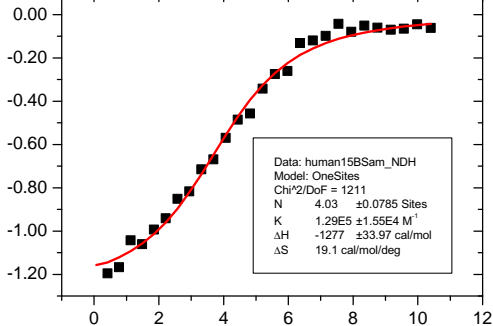

Molar Ratio

Supplement: Supplementary file 21 — Figure EV and appendix source Data [file 44318_2025_623_MOESM21_ESM.zip › Source data/Appendix Figure S6/Figure S6D/CPS15B-hCRP.PDF]

Time (min)

0 10 20 30 40 50 60 70 80 90 100

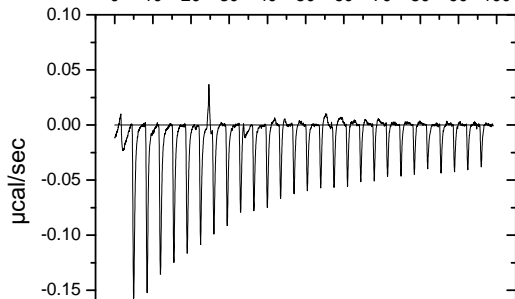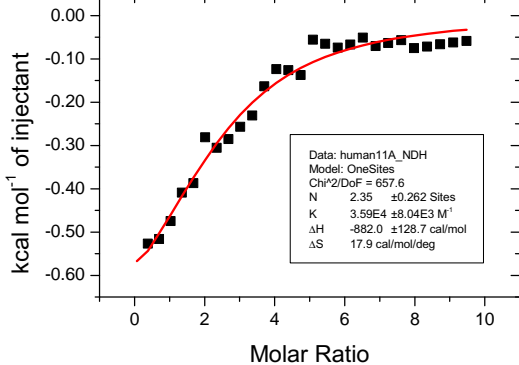

Supplement: Supplementary file 21 — Figure EV and appendix source Data [file 44318_2025_623_MOESM21_ESM.zip › Source data/Appendix Figure S6/Figure S6C/CPS11A-hCRP.PDF]

## Slide 1
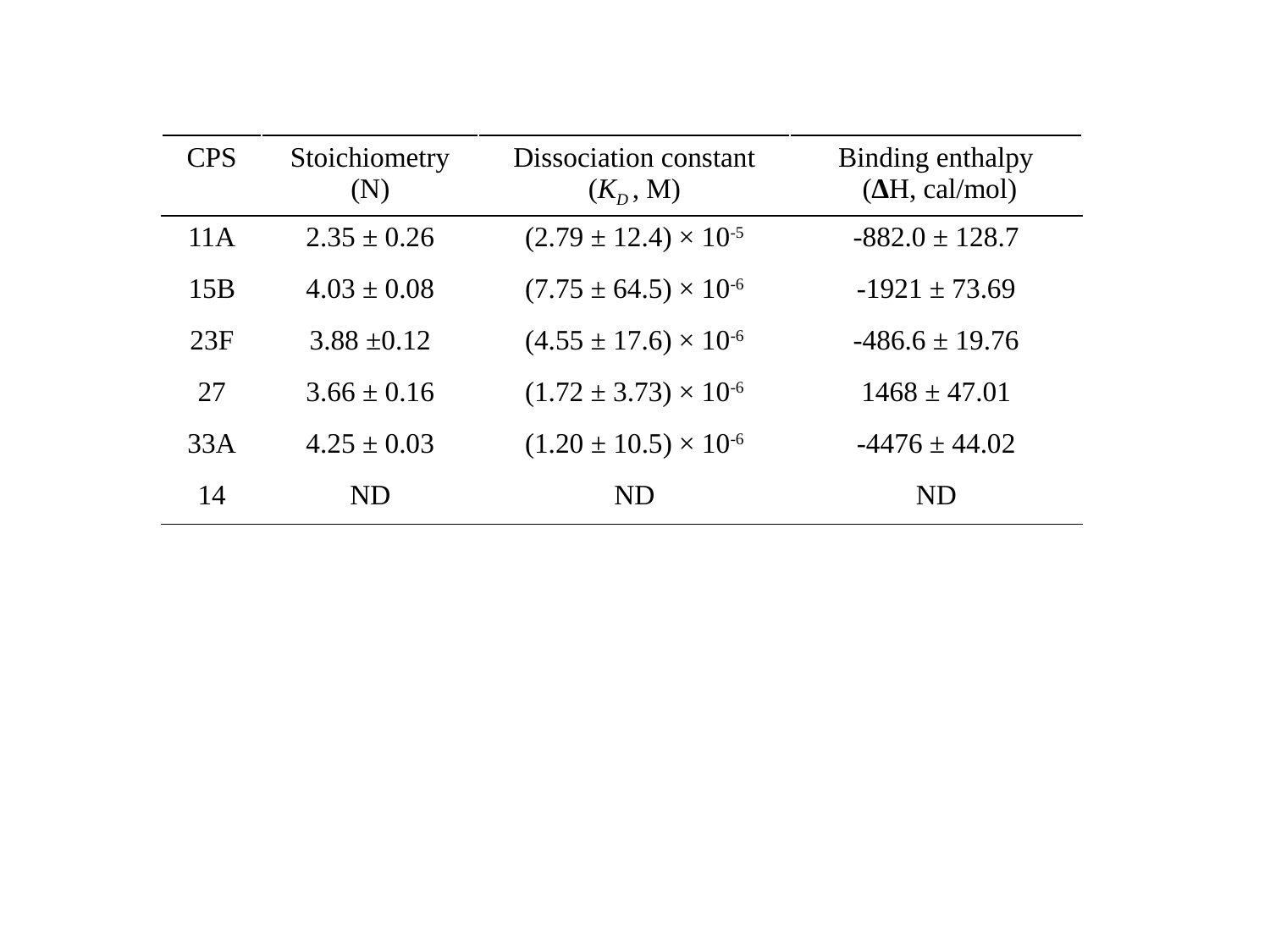

| CPS | Stoichiometry (N) | Dissociation constant (KD , M) | Binding enthalpy (ΔH, cal/mol) |
| --- | --- | --- | --- |
| 11A | 2.35 ± 0.26 | (2.79 ± 12.4) × 10-5 | -882.0 ± 128.7 |
| 15B | 4.03 ± 0.08 | (7.75 ± 64.5) × 10-6 | -1921 ± 73.69 |
| 23F | 3.88 ±0.12 | (4.55 ± 17.6) × 10-6 | -486.6 ± 19.76 |
| 27 | 3.66 ± 0.16 | (1.72 ± 3.73) × 10-6 | 1468 ± 47.01 |
| 33A | 4.25 ± 0.03 | (1.20 ± 10.5) × 10-6 | -4476 ± 44.02 |
| 14 | ND | ND | ND |

Supplement: Supplementary file 21 — Figure EV and appendix source Data [file 44318_2025_623_MOESM21_ESM.zip › Source data/Appendix Figure S6/Figure S6J/Figure S8J.pptx]

Time (min)

0 10 20 30 40 50 60 70 80 90 100

0.10

0.05

0.00

$\mu\text{cal/sec}$

-0.05

-0.10

$\text{kcal mol}^{-1}$  of injectant

0.30

0.00

-0.30

-0.60

-0.90

0

2

4

6

8

10

12

Molar Ratio

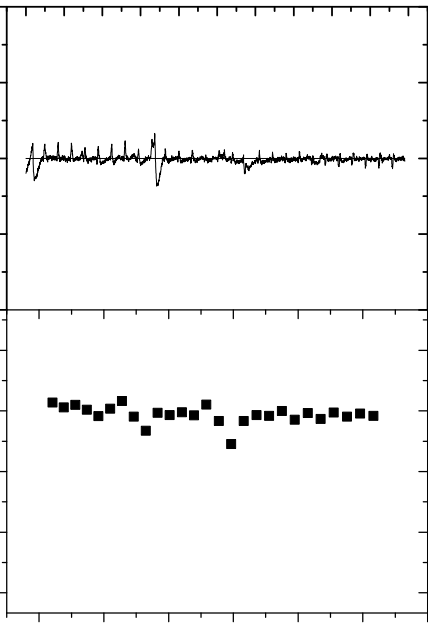

Supplement: Supplementary file 21 — Figure EV and appendix source Data [file 44318_2025_623_MOESM21_ESM.zip › Source data/Appendix Figure S6/Figure S6H/CPS14-hCRP.PDF]

Time (min)

0 10 20 30 40 50 60 70 80 90 100 110 120 130 140 150

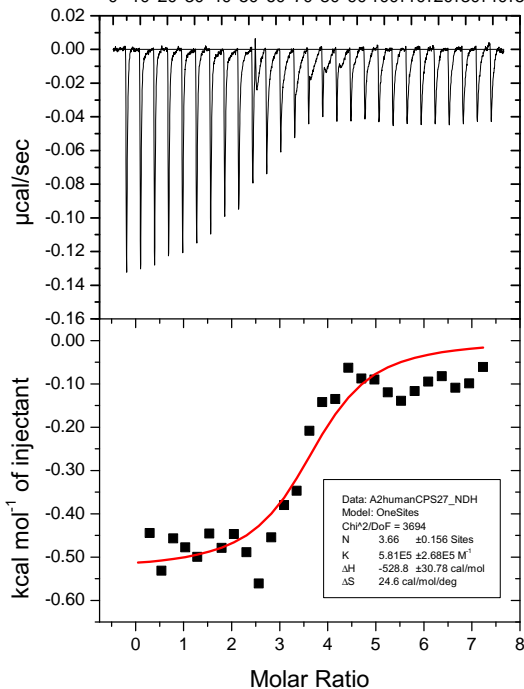

Supplement: Supplementary file 21 — Figure EV and appendix source Data [file 44318_2025_623_MOESM21_ESM.zip › Source data/Appendix Figure S6/Figure S6F/CPS27-hCRP.PDF]

Time (min)

0 10 20 30 40 50 60 70 80 90 100

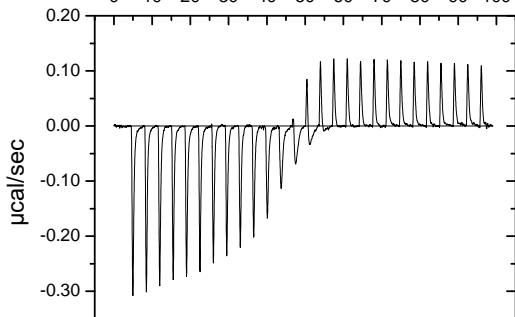

$\text{kcal mol}^{-1}$  of injectant

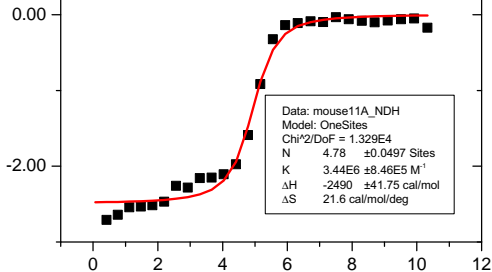

Molar Ratio

Supplement: Supplementary file 21 — Figure EV and appendix source Data [file 44318_2025_623_MOESM21_ESM.zip › Source data/Appendix Figure S6/Figure S6A/CPS11A-mCRP.PDF]

Time (min)

0 10 20 30 40 50 60 70 80 90 100

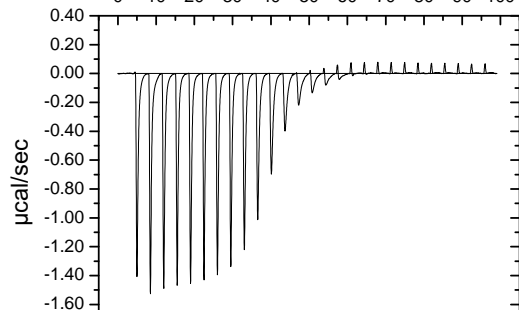

kcal mol<sup>-1</sup> of injectant

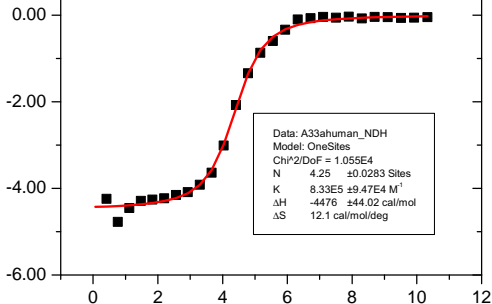

Molar Ratio

Supplement: Supplementary file 21 — Figure EV and appendix source Data [file 44318_2025_623_MOESM21_ESM.zip › Source data/Appendix Figure S6/Figure S6G/CPS33A-hCRP.PDF]

## Slide 1
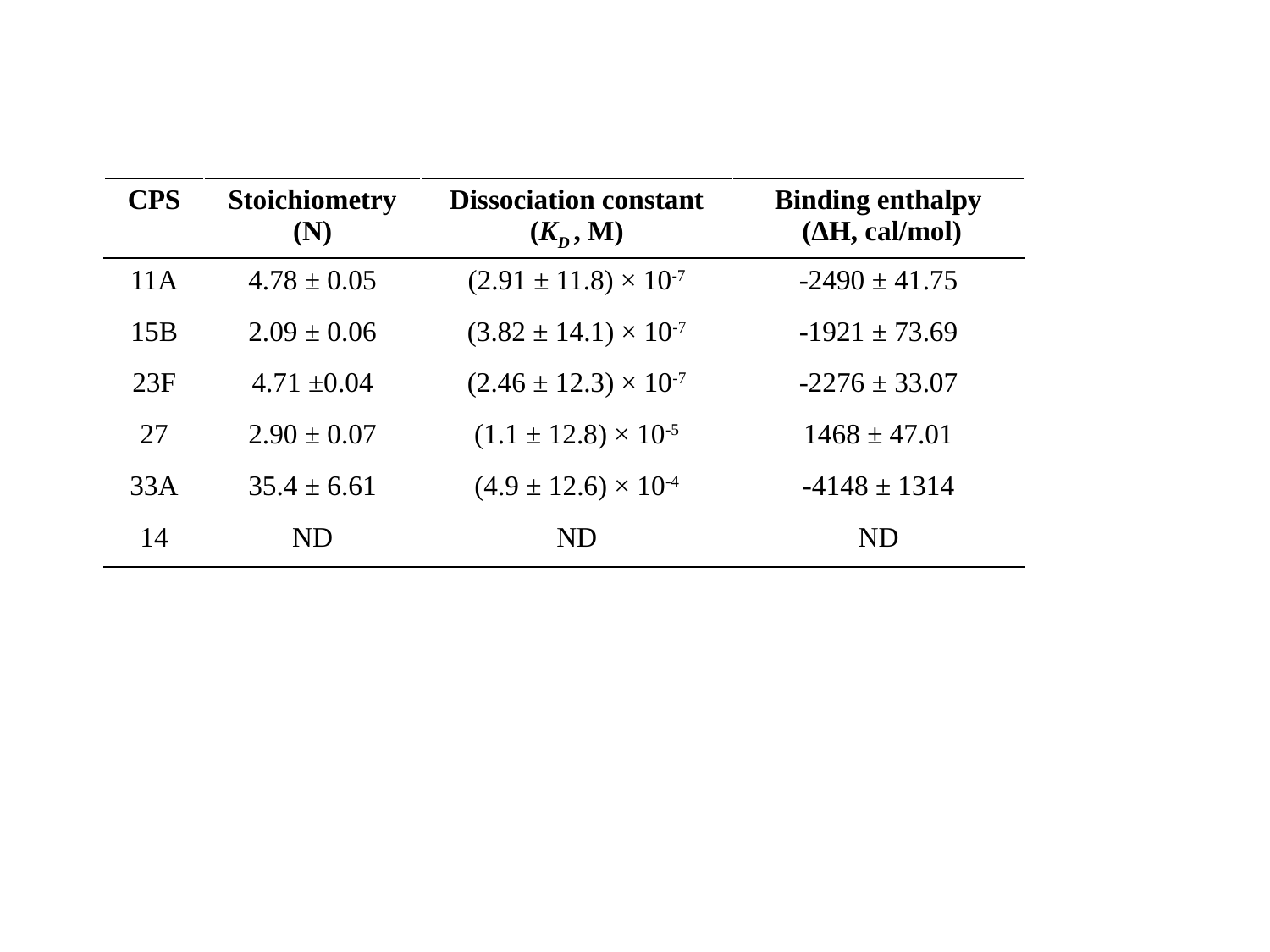

| CPS | Stoichiometry (N) | Dissociation constant (KD , M) | Binding enthalpy (ΔH, cal/mol) |
| --- | --- | --- | --- |
| 11A | 4.78 ± 0.05 | (2.91 ± 11.8) × 10-7 | -2490 ± 41.75 |
| 15B | 2.09 ± 0.06 | (3.82 ± 14.1) × 10-7 | -1921 ± 73.69 |
| 23F | 4.71 ±0.04 | (2.46 ± 12.3) × 10-7 | -2276 ± 33.07 |
| 27 | 2.90 ± 0.07 | (1.1 ± 12.8) × 10-5 | 1468 ± 47.01 |
| 33A | 35.4 ± 6.61 | (4.9 ± 12.6) × 10-4 | -4148 ± 1314 |
| 14 | ND | ND | ND |

Supplement: Supplementary file 21 — Figure EV and appendix source Data [file 44318_2025_623_MOESM21_ESM.zip › Source data/Appendix Figure S6/Figure S6I/Figure S8I.pptx]

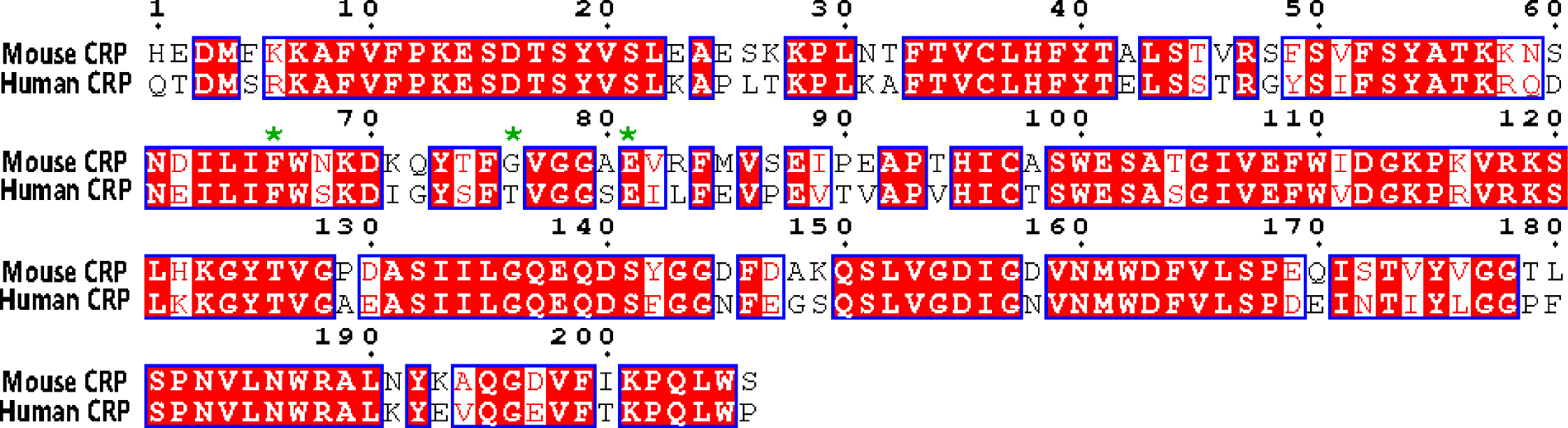

Supplement: Supplementary file 21 — Figure EV and appendix source Data [file 44318_2025_623_MOESM21_ESM.zip › Source data/Figure EV2/Figure EV2A/Figure EV2A.gif]
