# Supplementary material for: C-reactive protein is a broad-spectrum capsule-binding receptor for hepatic capture of blood-borne bacteria
Source: EMBO J. 2025 Nov 10;44(24):7364–94. doi: 10.1038/s44318-025-00623-w (PMC12705745; doi:10.1038/s44318-025-00623-w)
Supplement: Supplementary file 18 — Source data Fig. 6 [file 44318_2025_623_MOESM18_ESM.zip › SD figure 6/Figure 6B/CRP-CPS33A.PDF]

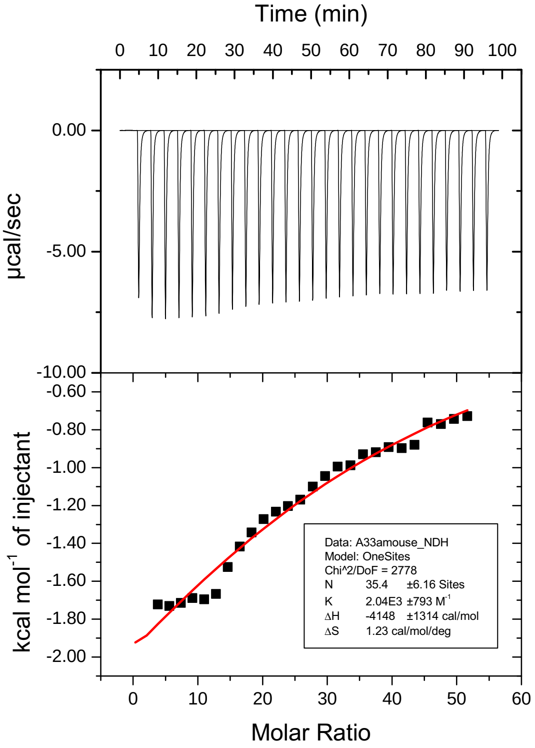

Time (min)
0 10 20 30 40 50 60 70 80 90 100
µcal/sec
0.00
-5.00
-10.00
kcal mol-1 of injectant
-0.60
-0.80
-1.00
-1.20
-1.40
-1.60
-1.80
-2.00
Data: A33amouse_NDH
Model: OneSites
Chi^2/DoF = 2778
N 35.4 ±6.16 Sites
K 2.04E3 ±793 M-1
ΔH -4148 ±1314 cal/mol
ΔS 1.23 cal/mol/deg
0 10 20 30 40 50 60
Molar Ratio
