# Supplementary material for: C-reactive protein is a broad-spectrum capsule-binding receptor for hepatic capture of blood-borne bacteria
Source: EMBO J. 2025 Nov 10;44(24):7364–94. doi: 10.1038/s44318-025-00623-w (PMC12705745; doi:10.1038/s44318-025-00623-w)
Supplement: Supplementary file 19 — Source data Fig. 7 [file 44318_2025_623_MOESM19_ESM.zip › SD figure 7/Figure 7F-H/Figure 7F-H.pptx]

## Slide 1
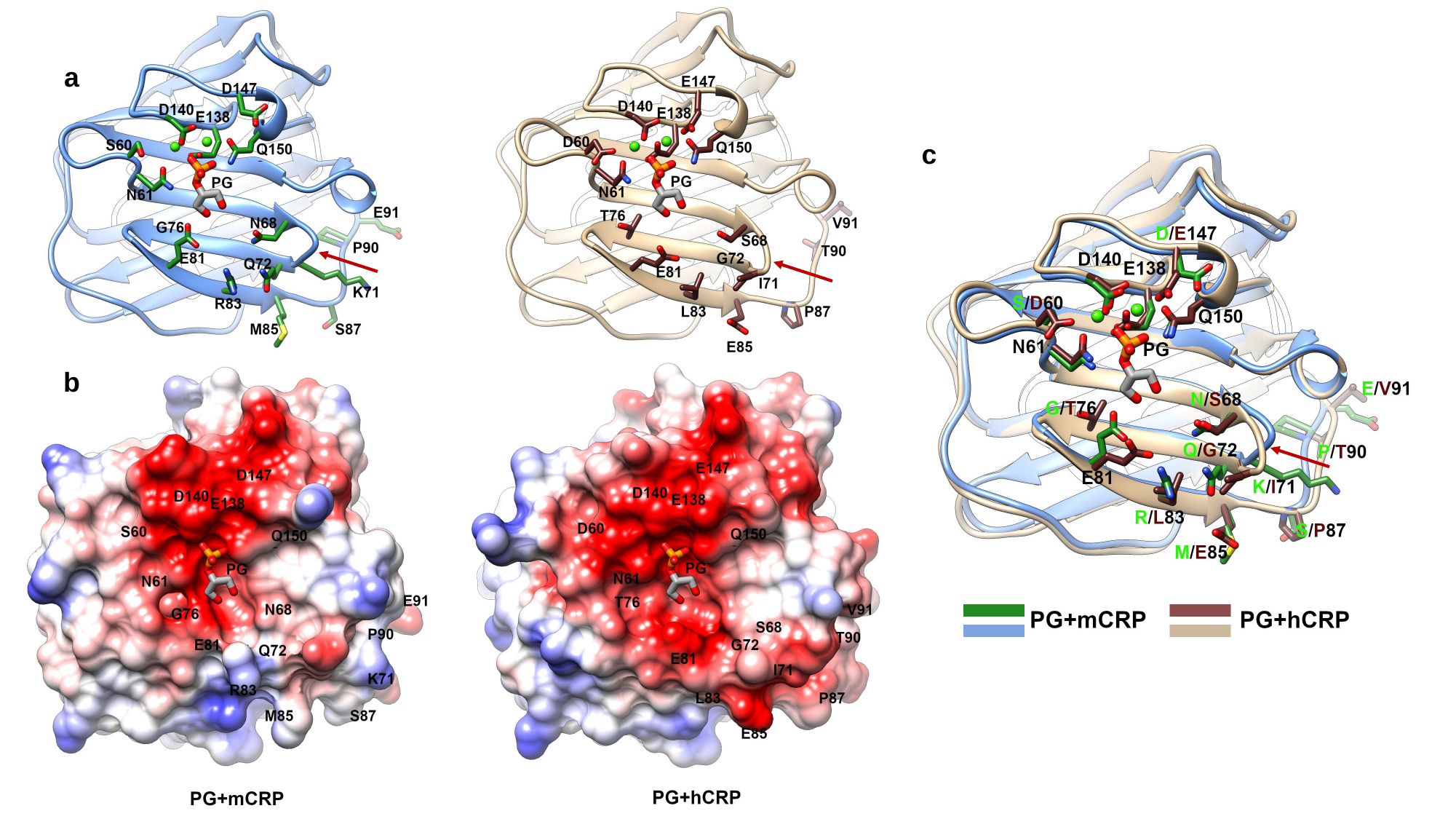

a
c
b

## Slide 2
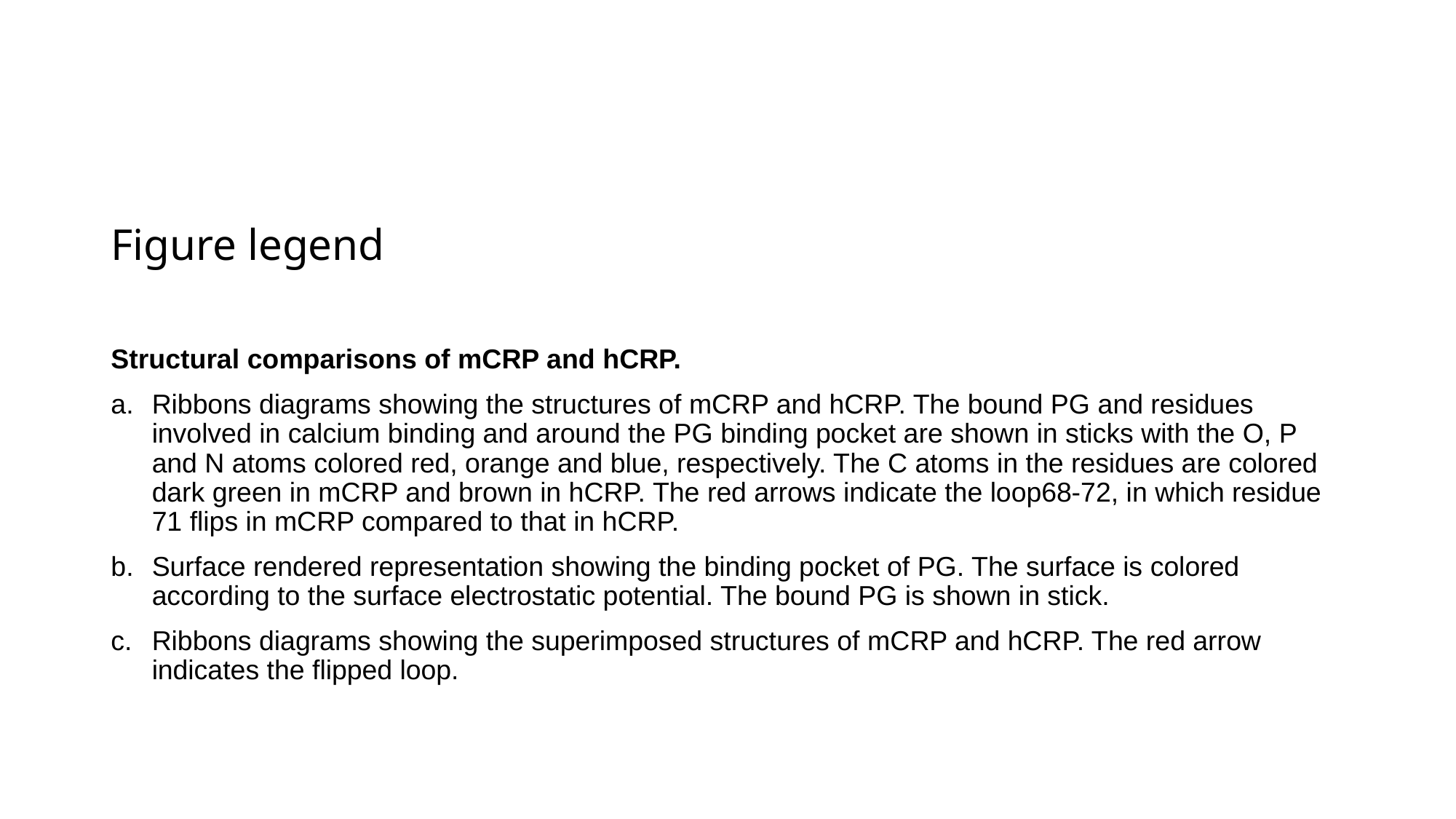

#
Figure legend
Structural comparisons of mCRP and hCRP.
Ribbons diagrams showing the structures of mCRP and hCRP. The bound PG and residues involved in calcium binding and around the PG binding pocket are shown in sticks with the O, P and N atoms colored red, orange and blue, respectively. The C atoms in the residues are colored dark green in mCRP and brown in hCRP. The red arrows indicate the loop68-72, in which residue 71 flips in mCRP compared to that in hCRP.
Surface rendered representation showing the binding pocket of PG. The surface is colored according to the surface electrostatic potential. The bound PG is shown in stick.
Ribbons diagrams showing the superimposed structures of mCRP and hCRP. The red arrow indicates the flipped loop.
