# Supplementary material for: C-reactive protein is a broad-spectrum capsule-binding receptor for hepatic capture of blood-borne bacteria
Source: EMBO J. 2025 Nov 10;44(24):7364–94. doi: 10.1038/s44318-025-00623-w (PMC12705745; doi:10.1038/s44318-025-00623-w)
Supplement: Supplementary file 23 — Expanded View Figures [file 44318_2025_623_MOESM23_ESM.pdf]

Expanded View Figures

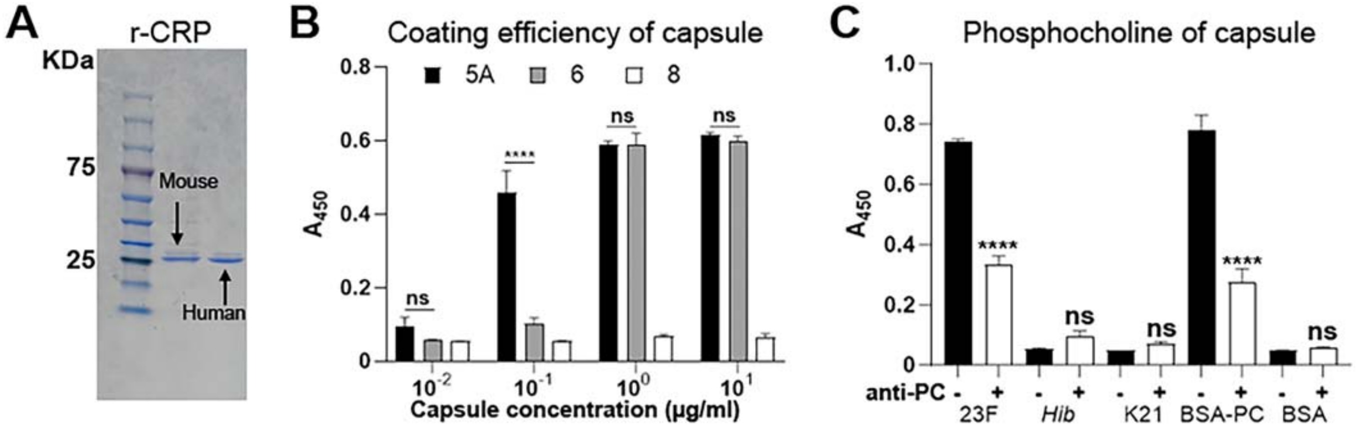

Figure EV1. Characterization of capsule coating efficiency and phosphocholine levels by ELISA.

(**A**) Construction of r-mCRP and r-hCRP. Purified r-mCRP and r-hCRP were detected by SDS-PAGE electrophoresis. $n = 2$. (**B**) Validation of capsule coating efficiency for ELISA. CPSs were coated onto plates at increasing concentrations (0.01, 0.1, 1, and 10 µg/ml, 100 µl/well) and detected using the serum from PCV13-immunized mice diluted at 1:300. Antibody binding was quantified by OD_{450} measurements. $A_{450\ nm}$ were presented as mean ± SEM of three biological replicates ($n = 3$). *P* value was calculated by Two-way ANOVA with Sidak's multiple comparisons (5 A vs 6) (*****P* < 0.0001, ns *P* > 0.05, no significance). *P* value: 10^{-2}, 0.5298; 10^{-1}, 1.14e−010; 10°, 0.9999; 10^{1}, 0.9291. (**C**) Detection of phosphorylcholine (PC) in capsule by ELISA. The black bars represent the level of PC detected in the capsule, while the white bars show the PC levels after treatment with anti-PC-IgG antibody. The capsules of *S. pneumoniae* serotype 23F, *H. influenzae* type b (*Hib*), and *K. pneumoniae* serotype K21, along with BSA-conjugated PC (BSA-PC) and BSA, were coated onto 96-well plates. PC were detected using a monoclonal anti-PC IgM antibody (1:3000), followed by an HRP-conjugated anti-mouse IgM secondary antibody (1:2000). Absorbance was measured at 450 nm (OD_{450}). $A_{450\ nm}$ were presented as mean ± SEM of three biological replicates ($n = 3$). *P* value was calculated by Two-way ANOVA with Sidak's multiple comparisons (anti-PC+ vs anti-PC-) (*****P* < 0.0001, ns *P* > 0.05, no significance). *P* value: 23F, 1.12e−009; *Hib*, 0.8068; K21, 0.9834; BSA-PC, 2.37e−011; BSA, 0.9997. Source data are available online for this figure.

 © The Author(s)

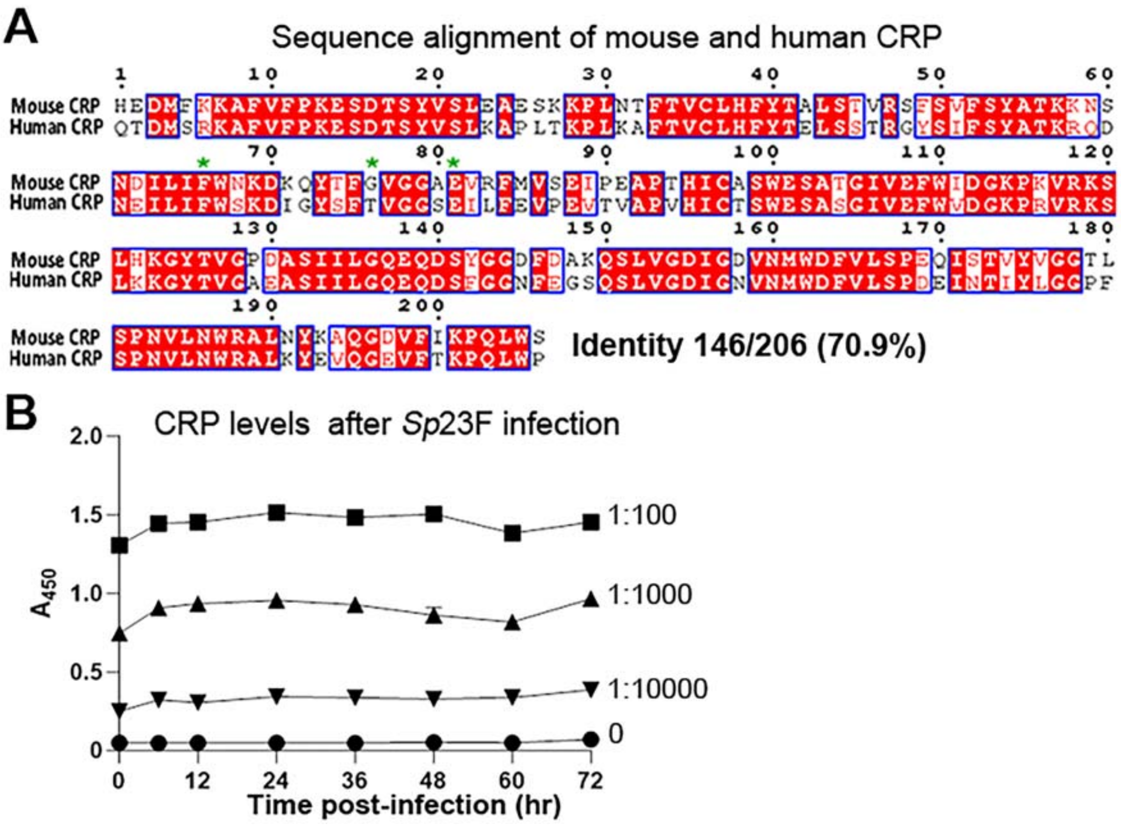

Figure EV2. Comparative analysis of mouse and human CRP.

(**A**) Sequence comparison between hCRP and mCRP. The amino acid sequences (without signal peptide) from mouse and human CRP were aligned by ClustalW software, and then the alignment results were imported into ENDscript/ESPript website to create the alignment figure. The conserved amino acids were indicted with red highlights. (**B**) ELISA measurement of serum CRP during the course of pneumococcal blood infection. Endogenous CRP levels in mouse serum were measured at the indicated time points post-*Sp*23F infection (0, 1, 6, 12, 24, 36, 48, 60, and 72 h). Serum samples were diluted at different concentrations (0, 1:100, 1:1000, 1:10,000) and analyzed using CRP-specific antibodies. $n = 3$. Source data are available online for this figure.

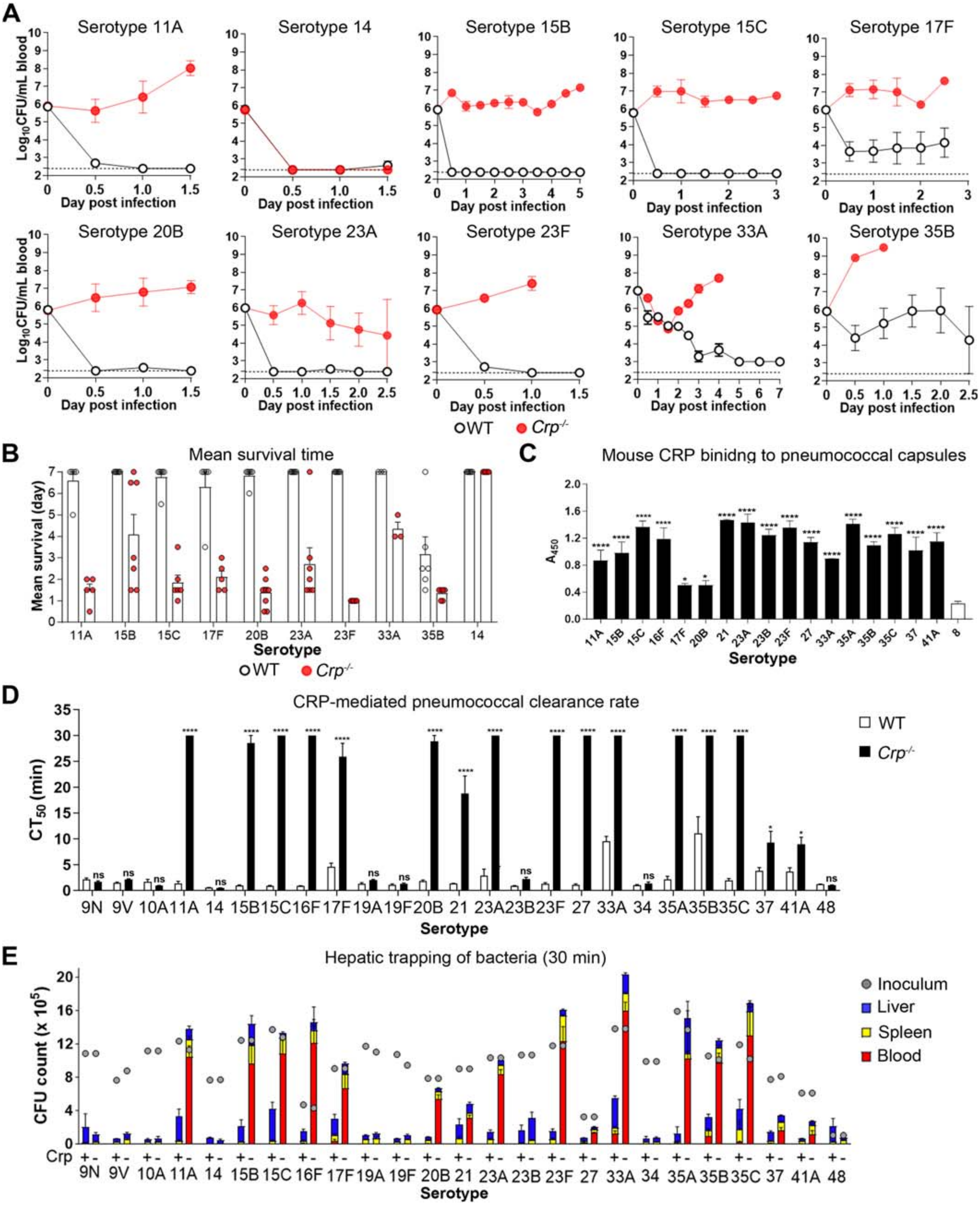
A
Serotype 11A
Serotype 14
Serotype 15B
Serotype 15C
Serotype 17F
Serotype 20B
Serotype 23A
Serotype 23F
Serotype 33A
Serotype 35B
Log10CFU/mL blood
Day post infection
WT
Crp-/-
B
Mean survival time
Mean survival (day)
Serotype
C
Mouse CRP binidng to pneumococcal capsules
A450
Serotype
D
CRP-mediated pneumococcal clearance rate
CT50 (min)
Serotype
WT
Crp-/-
E
Hepatic trapping of bacteria (30 min)
CFU count (x 10^5)
Inoculum
Liver
Spleen
Blood
Crp
Serotype

Figure EV3. The requirement of CRP for broad serotype-specific shuffling of many pneumococcal serotypes from the blood circulation to the liver.

(**A**) The bacteremia kinetics of WT and *Crp*$^{-/-}$ mice i.v. infected with 10 different serotypes. The blood CFU in individual mice were presented as mean ± SEM of three to eight biological replicates ($n = 3$–8). (**B**) Broad protection of CRP against septic infection of CRP-sensitive serotypes. WT and *Crp*$^{-/-}$ mice were i.v. infected with 10^6 CFU of *Sp*23F, *Sp*14, *Sp*11A, *Sp*15B, *Sp*15C, *Sp*17F, *Sp*20B, *Sp*23A, and *Sp*35B. Additionally, mice were i.v. infected with 10^7 CFU of *Sp*33A. The survival of WT and *Crp*$^{-/-}$ mice were assessed. Survival time were presented as mean ± SEM of three to eight biological replicates ($n = 3$–8). (**C**) mCRP binding to multiple pneumococcal capsules. The 96-well plates were individually pre-coated with purified pneumococcal capsules, and then incubated with 5 μg/ml r-mCRP. The CPS-bound CRP was then detected and represented by the absorbance at $A_{450\ nm}$ as in Fig. 1D. $A_{450\ nm}$ were presented as mean ± SEM of three to six biological replicates ($n = 3$–6). *P* value was calculated by One-way ANOVA with Sidak's multiple comparisons vs *S. pneumoniae* 8 (****$P < 0.0001$, *$P < 0.05$). *P* value: 11A, 3.36e−009; 15B, 3.27e−011; 15C, 2.06e−017; 16F, 3.75e−016; 17F, 0.0350; 20B, 0.04167; 21, 1.71e−017; 23A, 2.90e−018; 23F, 1.01e−018; 27, 7.66e−013; 33A, 1.10e−008; 35A, 1.01e−016; 35B, 3.41e−013; 35C, 1.04e−014; 37, 8.11e−011; 41 A, 5.34e−013. (**D**) The clearance rates of 25 low-virulence pneumococcal serotypes from the bloodstream in WT and *Crp*$^{-/-}$ mice infected i.v. with 10^6 CFU of each serotype. CT_{50} showed that *Crp*$^{-/-}$ mice significant delayed in clearing 16 of the 25 serotypes. CT_{50} were presented as mean ± SEM of three to five biological replicates ($n = 3$–5). *P* value was calculated by Two-way ANOVA with Sidak's multiple comparisons (WT vs *Crp*$^{-/-}$) (****$P < 0.0001$, ns $P > 0.05$, no significance). *P* value: 9N, 0.9999; 9V, 0.9999;10A, 0.9999; 11A, 1.98e−059; 14, 0.9999; 15B, 2.23e−050; 15C, 1.27e−055; 16F, 2.42e−040; 17F, 9.86e−038; 19A, 0.9999; 19F, 0.9999; 20B, 9.02e−044; 21, 2.52e−020; 23A, 1.65e−049; 23B, 0.9996; 23F, 1.75e−052; 27, 5.79e−040; 33 A, 4.01e−028; 34, 0.9999; 35A, 2.91e−038; 35B, 1.39e−032; 35C, 1.45e−038; 37, 0.0142; 41A, 0.02155; 48, 0.9999. (**E**) Serotype-specific distribution of viable bacteria in the blood, liver and spleen of WT and *Crp*$^{-/-}$ mice at 30 min post i.v. infection. The mice used to determine bacteremia kinetics in (**A**) were sacrificed at 30 min to quantify viable bacteria in the blood, liver and spleen by CFU plating. The inoculum of each group is indicated with a filled circle. Organ CFU in individual mice were presented as mean ± SEM of three to seven biological replicates ($n = 3$–7). Source data are available online for this figure.

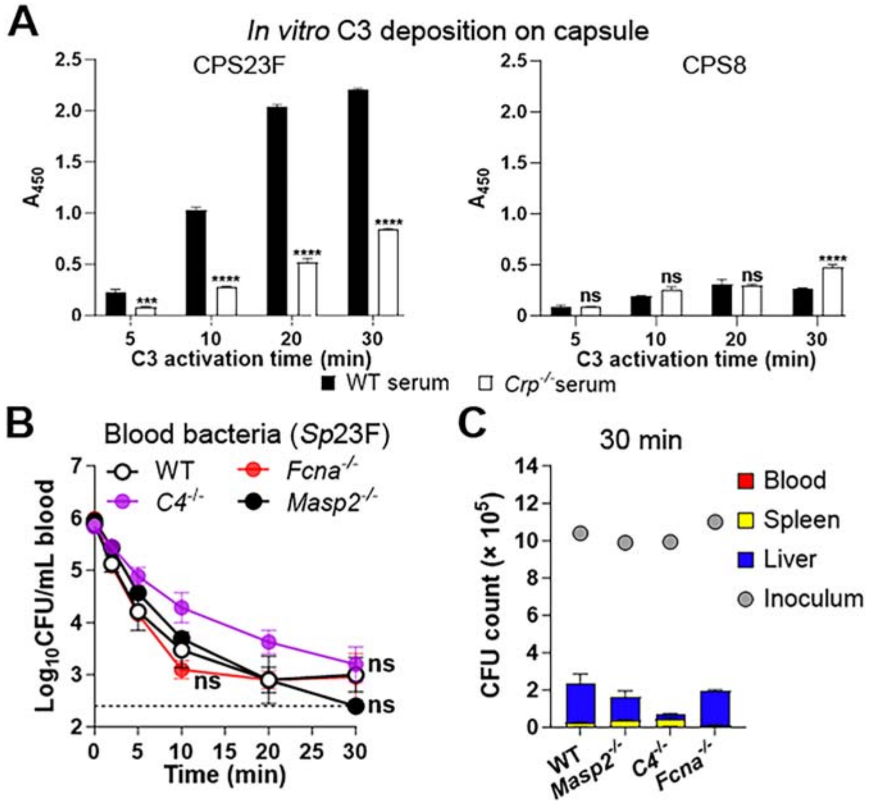

Figure EV4. Evaluation of CRP-activated C3 activation.

(**A**) CRP-activated C3 deposition on free capsular polysaccharide of serotype-23F *S. pneumoniae* was detected by ELISA. The 96-well plates were pre-coated with 10 μg/ml CPS23F or CPS8, and incubated with 100 μl of 10% serum at 37 °C for various durations. The abundance of C3 bound to capsular polysaccharides was detected with anti-C3 antibody. $A_{450\ nm}$ were presented as mean ± SEM of three to four biological replicates ($n = 4$). *P* value was calculated by Two-way ANOVA with Sidak's multiple comparisons (WT serum vs *Crp*$^{-/-}$ serum) (*****P* < 0.0001, ****P* < 0.001, ns *P* > 0.05, no significance). *P* value of CPS23F: 5 min, 0.0009; 10 min, 1.95e−017; 20 min, 1.43e−024; 30 min, 2.00e−023. *P* value of CPS8: 5 min, 0.9985; 10 min, 0.1811; 20 min, 0.9988; 30 min, 6.95e−006. (**B**, **C**) The role of the C3 pathway in *Sp*23F infection. WT, *Fcna*$^{-/-}$, *MASP2*$^{-/-}$, and *C4*$^{-/-}$ mice were infected i.v. with *Sp*23F of 10^6 CFU. The bacterial burden in the blood and major organs at 30 min was measured. The blood and organ CFU in individual mice were presented as mean ± SEM of three to six biological replicates ($n = 3$–6). *P* value was calculated by One-way ANOVA with Sidak's multiple comparisons vs WT (ns *P* > 0.05, no significance). *P* value: *C4*$^{-/-}$, 0.9015; *Masp2*$^{-/-}$, 0.9997; *Fcna*$^{-/-}$, 0.9996. Source data are available online for this figure.

 © The Author(s)

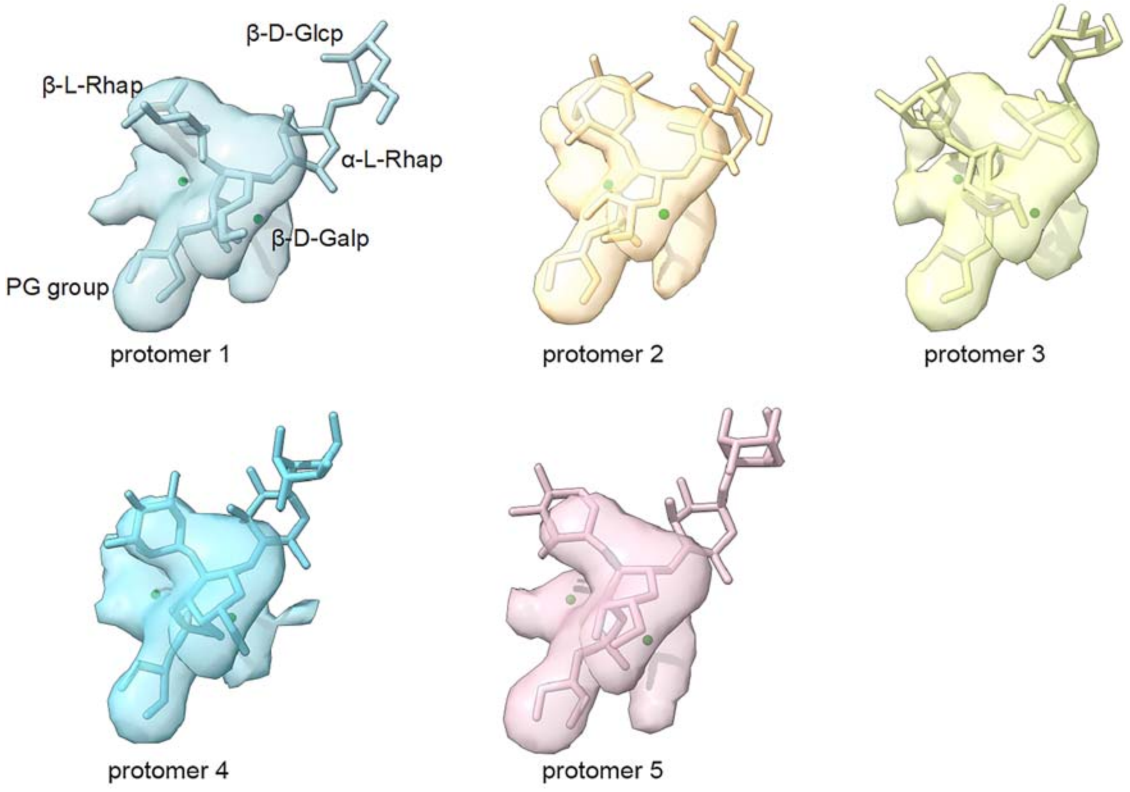

Figure EV5. Cryo-EM densities and atomic models of glycan ligand and associated calcium ions across five protomers in CRP.

Cryo-EM densities (contoured at 3σ) are identical for protomer 1-5 due to imposed C5 symmetry. Atomic models reveal conserved binding features: two sugar rings (β-L-Rhap and β-D-Galp) and the PG group of a repeat unit fit well within the density, while two remaining sugar rings (α-L-Rhap and β-D-Glcp) extend beyond the density, consistent with the ligand's intrinsic flexibility. Source data are available online for this figure.

© The Author(s)
